# Synthesis of 2-Cyanobenzothiazoles via Pd-Catalyzed/Cu-Assisted C-H Functionalization/Intramolecular C-S Bond Formation from *N*-Arylcyanothioformamides †

**DOI:** 10.3390/molecules27238426

**Published:** 2022-12-01

**Authors:** Nathan Broudic, Alexandra Pacheco-Benichou, Corinne Fruit, Thierry Besson

**Affiliations:** Univ Rouen Normandie, INSA Rouen, CNRS, COBRA UMR 6014, 76000 Rouen, France

**Keywords:** *N*-arylcyanothioformamides, 4,5-dichloro-1,2,3-dithiazolium chloride, *N*-arylimino-1,2,3-dithiazoles, 2-cyanobenzothiazoles, C-S bond formation

## Abstract

We report herein on a catalytic system involving palladium and copper to achieve the cyclization of *N*-arylcyanothioformamides and the synthesis of 2-cyanobenzothiazoles. The C-H functionalization/intramolecular C-S bond formation reaction was achieved in the presence of air, using 2.0 equiv of an inorganic additive (KI). In many cases, the reaction led to a sole product regioselectively obtained in good yields, allowing the synthesis of a wide range of substituted 2-cyanobenzothiazole derivatives, providing valuable building blocks for the design of more complex heterocyclic or molecular labeling systems.

## 1. Introduction

Benzothiazole is a heterocyclic system originally discovered in marine natural molecules and also present in terrestrial specimens. Its numerous applications in therapeutics are regularly patented and published in comprehensive reviews describing their role against metabolic, inflammatory, neurodegenerative, viral and bacterial diseases [1,2,3,4,5,6]. In recent years, researchers have focused their efforts on the anti-cancer potential of benzothiazoles or their derivatives [7,8,9,10,11,12,13].

All these studies have shown that the biological activities of benzothiazoles are highly dependent on the nature and position of their substituents. The most favorable positions are carbon C2, C5 and C6 of the benzothiazole skeleton, and the number of functional groups can vary from 1 to 3 and range from a simple chemical function to more complex aliphatic or heterocyclic systems [1,2,3,4,5,6,7,8,9,10,11,12,13]. It is important to note that the benzothiazoles showing significant biological activity are mainly substituted at the C2 position of the thiazole ring. In terms of antiproliferative activity, the most remarkable compounds are benzothiazole derivatives substituted by a nitrogen atom (e.g., amine, urea, hydrazone and semicarbazone), sulfur atom (e.g., sulfanyl derivatives), or substituted aromatic group or a hetero-aromatic group (e.g., thiazole, pyridine, imidazole, pyrazole and oxazole). All these efforts led to numerous innovative synthetic routes for preparing such compounds [14,15,16].

Among this important heterocyclic family, 2-cyanobenzothiazoles (also called 1,3-benzothiazole-2-carbonitriles) are of particular interest. Despite some studies on their potential antiproliferative activity on cancer cells [17,18,19], research interest lies mainly in the ability of the carbonitrile function to react under nucleophilic attacks, thus allowing easy access to various functions such as amides, imidates, amidines, carboxylic acids and esters [20,21,22,23]. Moreover, it has been shown that the nitrile function can also be easily eliminated in acidic conditions (HCl or HBr) via a hydrolysis-decarboxylation sequence, allowing further arylation reactions at C2 via CH-activation methods [24,25]. In the last decade, 2-cyanobenzothiazoles substituted at the C6 position by a primary amine or hydroxyl group have become tools of choice in the development of click chemistry methods targeting cysteine residues under physiological and biocompatible conditions. This strategy was applied for the in situ assembly or self-assembly of biomolecules and nanostructures for various applications in drug targeting and delivery, specific labeling of peptides and biorthogonal ligation reactions [26,27,28,29,30,31,32,33].

There are several synthetic routes for preparing 2-cyanobenzothiazoles in the literature [34,35,36,37,38,39,40,41,42,43,44,45]. The seminal work of White and colleagues outlined their synthesis, mainly focusing on luciferin-based derivatives that are mono-substituted at the C6 position with electron-donating groups (OMe, OH or NH_2_) [34,35]. Most of the time, the multi-step syntheses included in the final step the Rosemund-von Braun and Sandmeyer reactions from 2-iodo-, 2-chloro- and 2-amino-6-substituted benzothiazoles [36,37,38], respectively, involving toxic cyanide as a reactant. Alternatively, 6-methoxy-1,3-benzothiazole-2-carboxamide was used as a precursor to produce the expected cyanated products [39]. Less conventional synthetic routes have also been described in studies on the cyanation of heterocyclic compounds. In most cases, only unsubstituted 2-cyanobenzothiazole was reported as an example of the application of the studied methodology [40,41,42,43,44,45].

**Figure 1:**
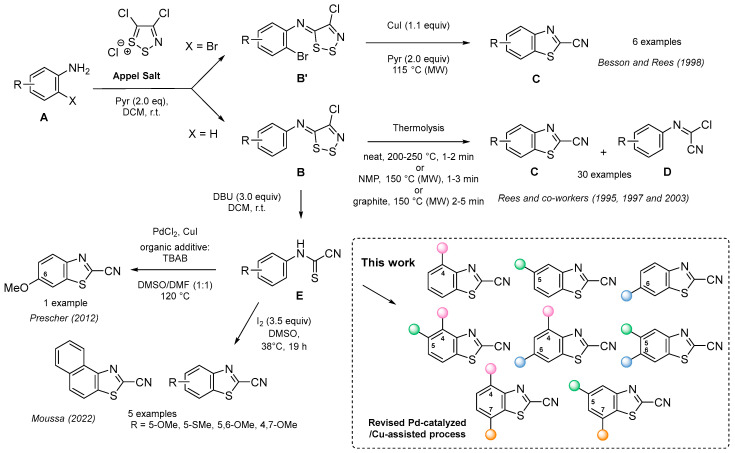
Synthesis of 2-cyanated benzothiazoles from 5-*N*-arylimino-4-chloro-1,2,3-dithiazoles and *N*-arylcyanothioformamides and description of the present work. References involved in these studies: [47,48,51,52,55,57].

One of the most popular methods described in the last 10 years for the synthesis of 2-cyanobenzothiazoles was inspired by the work of Rees and colleagues [46,47,48]. In the early 1990s, they studied the chemistry of 4,5-dichloro-1,2,3-dithiazolium chloride (Appel salt) [49] and described the synthesis of 2-cyanated benzothiazoles (C) by thermolysis of 5-*N*-arylimino-4-chloro-1,2,3-dithiazoles (B) [50,51], obtained by the reaction of aromatic amines (A) with Appel salt (Figure 1).

These studies demonstrated that strong electron-withdrawing groups on the starting anilines can modify the cyclization process and lead to a large number of undesired products such as cyanoimidoyl chloride (D in Figure 1). Seeking to overcome the influence of substituents, the same group described the copper(I)-mediated and regioselective cyclization of imino-1,2,3-dithiazoles (B’) resulting from the condensation of substituted *o*-bromoanilines with Appel salt [52]. These efficient reactions were rapidly performed at atmospheric pressure with a focused microwave reactor or under traditional heating, albeit for a longer time. All these studies have outlined the difficulties in obtaining the relevant *o*-brominated reagents to provide the target 2-cyanobenzothiazoles.

Some years later, drawing on the preceding work by Doi on the synthesis of 2-arylbenzothiazoles by the cyclization of thiobenzanilides [53,54], Prescher and colleagues, described a convenient method for preparing 6-methoxybenzothiazole-2-carbonitrile (Figure 1), allowing three-step access to bioluminescent luciferin derivatives [55,56]. In these studies, the condensation of *p*-anisidine with Appel salt led to the corresponding 4-chloro-*N*-(4-methoxyphenyl)-5*H*-1,2,3-dithiazol-5-imine, which was converted to its cyanothioformamide analogue (also called cyanothioformanilide) (E in Figure 1). Benzothiazole ring closure was performed with palladium chloride (PdCl_2_) and copper iodide (CuI) as catalysts with tetrabutylammonium bromide (TBAB) as an organic additive, in a mixture of DMSO/DMF (1:1, *v*/*v*) as the solvent [55,56]. Recently, Moussa et al. investigated the efficiency of I_2_-DMSO as an oxidative system and described the unexpected conversion of some *N*-arylcyanothioformamides into 2-cyanobenzothiazoles (five examples, Figure 1) [57].

For the last 10 years, our group investigated the chemical application of Appel Salt and its 5-*N*-arylimino-4-chloro-1,2,3-dithiazole derivatives for fusing the 2-cyanobenzothiazole motif on pyrimidine or pyrimidinone systems, and synthesizing bioactive thiazoloquinazolines and quinazolinones, which are able to affect the activity of kinases involved in neurodegenerative diseases (Alzheimer’s disease, Down’s syndrome) and cancers [58,59,60,61]. Recently a new strategy in our molecular and biological targets incited us to develop more practical and efficient general synthetic protocols. It appeared relevant and useful to allow easy access to diversely substituted and functionalized 2-cyanobenzothiazole derivatives. The present study thoroughly investigates a convenient palladium-catalyzed and copper-assisted method for the synthesis of a large array of these compounds and improves upon the existing literature. It also aims at exploring the regioselectivity of the thiazole ring closure under the steric or electronic influence of substituents present on the starting anilines. For the first time, a reaction mechanism is suggested in adequation with the data obtained (Figure 1).

## 2. Results and Discussion

In the present study, preliminary experiments were carried out to explore the synthetic route to the 6-methylbenzo[*d*]thiazole-2-carbonitrile **4a**. *p*-Toluidine **1a** was condensed with Appel salt (1.1 equiv) in the presence of pyridine (2.0 equiv) in dichloromethane (DCM), at room temperature (r.t.) for 1 h, to give the imino-1,2,3-dithiazole **2a**. The compound **2a** was then treated by 3 equiv of 1,8-diazabicyclo [5.4.0] undec-7-ene (DBU) [62] to produce the corresponding *N*-(4-methylphenyl)cyanothioformamide **3a** in convenient yields (46%) (see Table 1).

In a first attempt, based on the experimental conditions described by Doi [53,54] and Prescher [55,56]; **3a** was solubilized in DMSO/DMF (1:1, *v*/*v*, [0.025 or 0.050 M]) and heated at 120 °C for 4 h in the presence of 10 or 20 mol% of PdCl_2_, 50 mol% of CuI and 2 equiv of tetrabutylammonium bromide (TBAB), as depicted in Table 1.

The best result was obtained with 20 mol% of PdCl_2_ and a starting molar concentration of 0.025 M in the solvent mixture. Under these conditions, the expected benzothiazole **4a** was obtained with a 49% yield (entry 4). Expecting improvement, TBAB was replaced by tetrabutylammonium iodide (TBAI) and produced **4a** a similar yield (51%).

In the preceding work [53,54], Doi and colleagues discovered that the addition of an inorganic additive such as CsF led to a significant improvement in the C-H functionalization/intramolecular C-S bond formation reaction from thiobenzanilides. We, therefore, replaced TBAB by 2 equiv of CsF, but this provided **4a** in only a 15 % yield (entry 1 in Table 2). Based on these preliminary results, inorganic additives in place of TBAB or CsF were screened. Table 2 reports our results with various inorganic salts (2.0 equiv) added to the reaction mixture. The other reactants and solvent proportions remained unchanged.

Among the salts tested, cesium derivatives (CsF and CsI) were found to be the least effective additives (entries 1 and 2) while sodium, potassium and lithium salts produced good results, producing the desired 6-methyl-2-cyanobenzothiazole **4a** in moderate to good yields (53–70%) (entries 3-5, 7 and 9), except in the case of KF and LiCl, which led to yields of 16 and 33%, respectively (entries 6 and 8). In our case, KI gave the best results leading to **4a** with a 70% yield (entry 5). Atmospheric oxygen plays a crucial role since an inert atmosphere (argon) gave a lower yield of 23% (see footnote 2 for entry 5). Increasing the quantity of KI (3.0 equiv) also had a negative effect on the yield of the reaction, which fell to 39% (see footnote 3 for entry 5). It is noteworthy that in the optimizing experiments described by Doi et al. [53], LiBr gave the expected product in the same yield as that obtained with TBAB. In our case, LiBr allowed the synthesis of **4a** in only a 53% yield (entry 7). Entry 10 confirms that the additive is required to obtain **4a** in a good yield.

To complete the optimization of the reaction conditions, various sources of palladium and copper were also tested. Solvent and co-solvent were also investigated, as depicted in Table 3.

None of the new conditions tested improved the reaction efficiency except PdBr_2_, which led to a similar yield to PdCl_2_ (entry 2). Using Pd(OAc)_2_ and Pd_2_dba_3_ drastically decreased the quantity of **4a** obtained (22 and 7%, respectively) (entries 3 and 4). The absence of a palladium source in the reaction mixture gave no reaction (entry 5) while the lack of copper led to a lower yield (41% instead of 70%) (entry 9), confirming the need for these components in the chemical equation. Out of all the solvents tested, the initial mixture of DMSO/DMF in equal proportions remained the best for this reaction (entry 1 compared to entries 10, 11 and 12).

Table 4 also reports the results obtained when initial amounts of palladium chloride (PdCl_2_) and copper iodide (CuI) were optimized, as well as the starting molar concentration [c] in the solvent mixture. It confirms that heating the starting cyanothioformamide **3a** at 120 °C for 4 h in DMSO/DMF (1:1, *v*/*v*; [0.025 M]), in the presence of 20 mol% PdCl_2_, 50 mol% CuI and 2 equiv of potassium iodide (KI), was the most efficient method for the synthesis of **4a** (entry 1). In all cases, changing the initial concentration of PdCl2 or CuI, or the amount of solvent, led to lower yields (entries 2, 3, 4 and 5).

Considering our experience investigating the role of microwaves in the thermal activation of chemical reactions [63], a series of tests were performed in a microwave reactor operating at atmospheric pressure. Compound **4a** was synthesized by applying the same operating parameters (quantities of reagents, solvent, temperature) as those described above. The programmed temperature was controlled by an external infrared pyrometer, which allowed feedback control of the power input in the cavity. A TLC control showed the disappearance of the reagents after 1 h of irradiation, and no change was observed on prolonged heating. Compound **4a** was isolated in a lower yield (57%) than under the standard thermal conditions (70%).

**Scheme 1:**
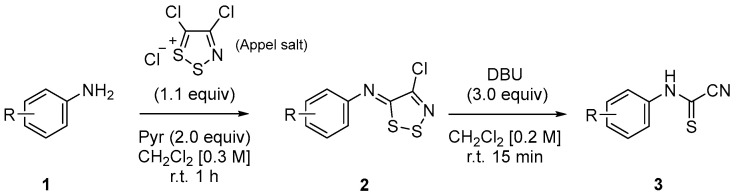
Synthesis of dithiazoles **2** and cyanothioformanilides **3** from anilines **1**.

With the optimized conditions identified, the scope of *N*-arylcyanothioformamides **3** was explored to generate a valuable array of variously substituted 2-cyanobenzothiazoles. As described above for **3a**, all *N*-arylcyanothioformanilides **3** were obtained using a two-step procedure in which starting anilines **1** were stirred with Appel salt (1.1 equiv) and pyridine (2.0 equiv) in dichloromethane (DCM), at r.t. for 1 h, to give the corresponding imino-1,2,3-dithiazoles **2**, which were then treated by 3 equiv of 1,8-diazabicyclo [5.4.0] undéc-7-ene (DBU) in DCM at r.t. for 15 min (Scheme 1) [62].

Yields are reported in Table 5; for detailed procedures and physicochemical characterization see the Supplementary Materials. Note that the sequential one-pot process previously described by Prescher et al. for the preparation of **3f** (4-OMe) and **3j** (4-NO_2_) [56] was not applicable to this range of anilines.

**Scheme 2:**
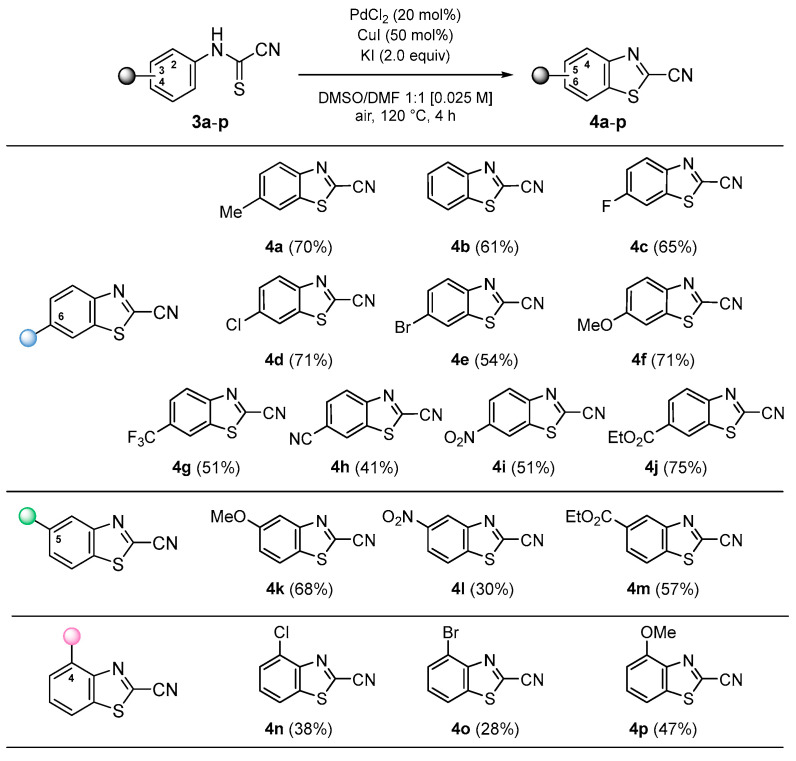
Synthesis of 6, 5 and 4-mono-substituted 2-cyanobenzothiazoles (**4a-p**) from corresponding 4, 3 and 2-mono-substituted anilines (**1a-p**) (isolated yields).

Firstly, 2-, 3- or 4-mono-substituted cyanothioformamides **3a-p** were transformed into the corresponding 4-, 5- and 6-mono-substituted 2-cyanobenzothiazoles **4a-p** according to the already optimized C-H functionalization/intramolecular C-S bond formation reaction (Scheme 2).

The yields obtained with substituents positioned at C6 were quite good, ranging from 41% for the bis-cyanated derivative **4h** to 71% for **4f** with an electron donor group (e.g., OMe). The electronic effect of the substituents at the C3 position of the starting cyanothioformamide slightly affected the yields in the resulting C5-substituted 2-cyanobenzothiazoles such as **4k**, **4l** and **4m**. Nevertheless, the 5-methoxybenzothiazole-2-carbonitrile **4k** was then obtained in a similar yield as that of its 6-substituted isomer **4f** (68 and 71%, respectively). Notably, whatever the *N*-cyanothioformamide reagent, no C4-substituted 2-cyanobenzothiazole regioisomer was obtained, suggesting a regiospecific cyclization process. Moreover, despite a deactivating effect on the aromatic ring, the ethyl carboxylate group was found to be compatible and 2-cyanobenzothiazoles **4j** and **4m** were isolated in good yields (75 and 57%, respectively). The yields obtained for the 5- and 6-nitrobenzothiazole-2-carbonitriles **4l** and **4i** produced a more significant difference of 30 and 51%, respectively.

**Scheme 3:**
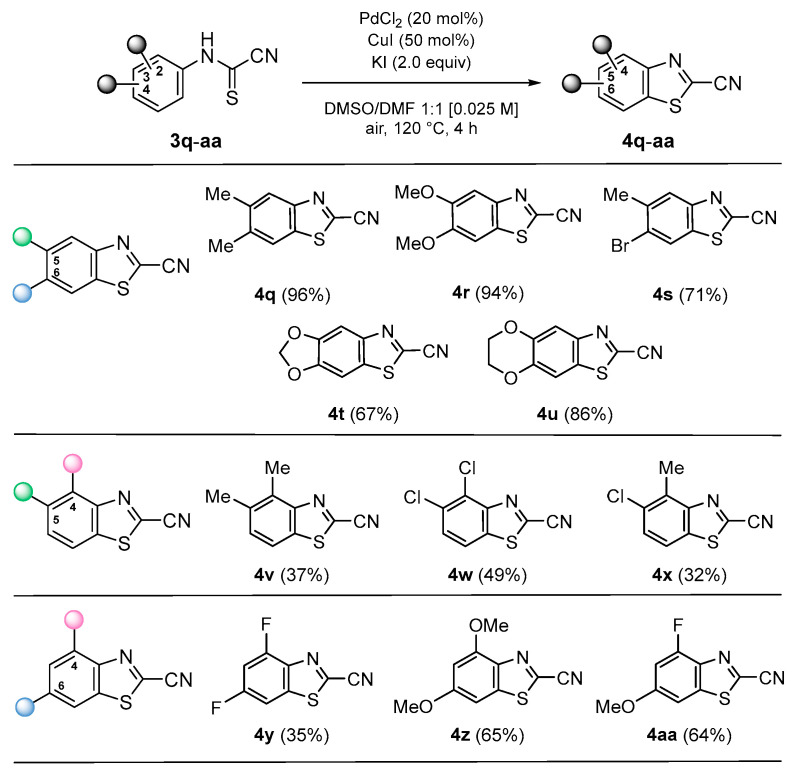
Synthesis of 5,6-, 4,5- and 4,6-disubstituted 2-cyanobenzothiazoles (**4q-4aa**) from corresponding 3,4-, 2,3 and 2,4-disubstituted anilines (**1q-1aa**) (isolated yields).

To increase the range of 2-cyanobenzothiazoles, derivatives di-substituted in the 5,6—, 4,5- and 4,6-positions (compounds **4q-4x**) were prepared from the corresponding *N*-arylcyanothioformamides **3q-3x**, difunctionalized in the 3,4-, 2,3- and 2,4 -positions (Scheme 3).

Di-substituted benzothiazoles in positions 5 and 6 were obtained in good (67% for **4t**) to excellent yields (e.g., 96 and 94% for **4q** and **4r**, respectively). In these cases, the substituents were mainly activating groups while a bromide in *p*-position for the nitrogen atom led to a decrease in the yield (71% for **4s** compared with 96% for **4q**). This result is in accordance with those described in Scheme 1 for **4a** and **4e** with yields of 70 and 54%, respectively. Microwave-assisted synthesis of **4q** and **4s** was also tested and confirmed the results previously obtained for **4a**.

The synthesis of 4,5-di-substituted 2-cyanobenzothiazoles (**4v**, **4w** and **4x**) proved to be more difficult and yields were lower than those obtained with the 5,6-disubstituted compounds. However, these results are close to those obtained in the synthesis of benzothiazoles **4n-4p** from cyanothioformanilides **3n-3p**, which are substituted in position 2. These results suggest a significant steric effect when substituents are in the C2 position of the reagent. This effect is apparently counterbalanced by the electron-donor effect of the substituents in the C4 position, as shown by the data obtained for the synthesis of 2-cynobenzothiazoles **4z** and **4aa**.

**Scheme 4:**
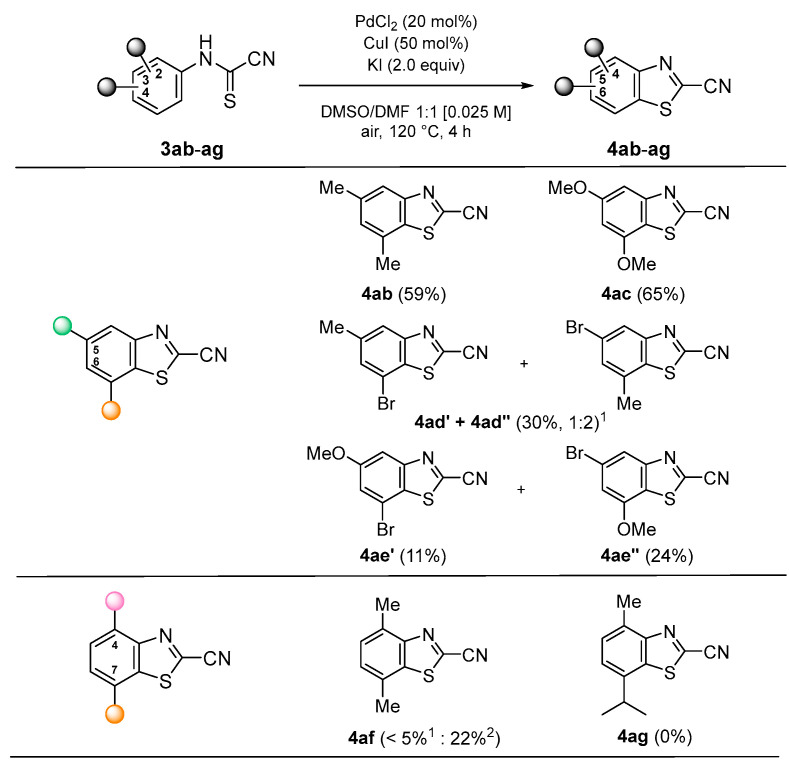
Synthesis of 5,7- and 4,7-di-substituted 2-cyanobenzothiazoles (**4q-4aa**) from corresponding 3,5-, and 2,5-di-substituted cyanothioformamides (**3ab-3ag**) (isolated yields). ^1^ Yields calculated from ^1^H-NMR. ^2^ KI was replaced by LiBr.

To complete our study, access to 5,7- and 4,7-substituted 2-cyanobenzothiazoles (**4ab-4ag**) was studied from the corresponding cyanothioformanilides (**3ab-3ag**) (Scheme 4).

Compounds **4ab** and **4ac** were obtained in good yields of 59 and 65%, respectively. In contrast, when the reaction was produced using dissymmetric cyanothioformamides on the C3 and C5 positions (**3ad** and **3ae**), a mixture of regioisomers was obtained in an about 30% yield in both cases. The benzothiazoles **4ae’** and **4ae’’** were separated by flash column chromatography and isolated in 11 and 24% yields. However, compounds **4ad’** and **4ad’’** could not be separated regardless of the techniques used. The intramolecular C-S bond formation sequence predominantly occurred on the side of the electron-donor substituent with a ratio of 2:1 to the other partner. Unfortunately, the developed method failed to cyclize the cyanothioformamides disubstituted in C2 and C5 (**3af** and **3ag** in Scheme 4).

Cyanobenzothiazole-2-carbonitrile **4af** was obtained in only a 22% yield when KI was replaced by 2.0 equiv of LiBr.

This result suggests that depending on the reagents, the size of the inorganic additive may influence the yield of this regiospecific reaction. In the case of **4a** (Table 2), no steric constraints were present and KI was more efficient than LiBr. In contrast, for the cyanothioformamide **3af**, steric hindrance prevented KI from playing its role in the reaction. Nevertheless, this yield was still lower than the one previously obtained by our group (58%) when compound **2af** was subjected to microwave-assisted thermolysis at 150 °C in *N*-methylpyrrolidinone (NMP) [51]. Notably, Prescher also observed the same drawback and finally heated 2,5-disubstituted cyanothioformamides at 170 °C in sulfolane to obtain the attempted 4-bromo-7-methyl-benzothiazole-2-carbonitrile derivatives, also in low yields (10–20%) [32].

**Scheme 5:**
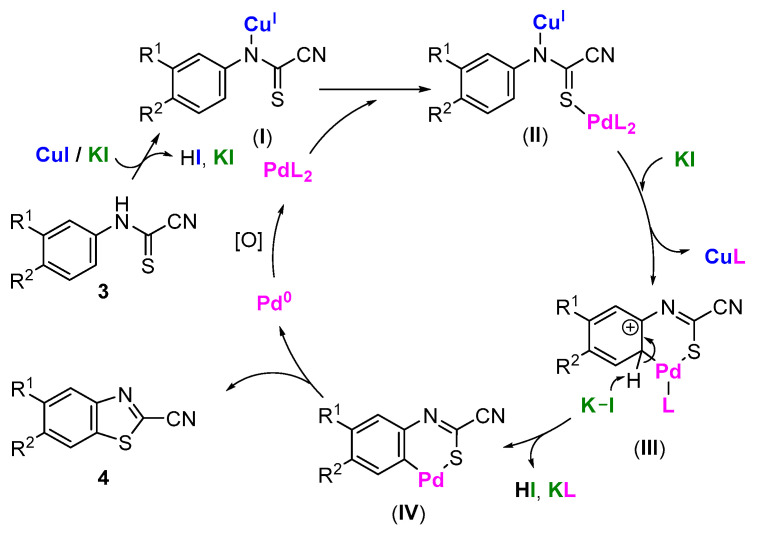
Suggested mechanism for synthesis of 2-cyanobenzothiazoles (**4**) from *N*-arylcyanothioformamides (**3**).

Scheme 5 depicts the suggested mechanism, based on these results and the literature data [63,68].

The reaction is most likely initiated by the base-assisted formation of the Cu^(I)^ thioamidate (**I**), favoring the coordination of Pd^(II)^ with the sulfur atom to form the intermediate (**II**). Then, a deprotonative metalation step occurs, forming a sterically hindered transition state driving the regioselectivity (**III**), to obtain the palladacycle (**IV**), which undergoes reductive elimination, leading to the desired 2-cyanobenzothiazole **4** and Pd^(0)^, which can be reoxidized by atmospheric oxygen.

## 3. Materials and Methods

### 3.1. General Information

All reagents were purchased from commercial suppliers and used without further purification. All reactions were monitored by thin-layer chromatography with aluminum plates (0.25 mm) precoated with silica gel 60 F254 (Merck KGaA, Darmstadt, Germany). Visualization was performed with UV light at a wavelength of 254 nm. Purifications were conducted with a flash column chromatography system (PuriFlash, Interchim, Montluçon, France) using stepwise gradients of petroleum ether (also called light petroleum) (PE) and dichloromethane (DCM) as the eluent. Melting points were measured with an SMP3 Melting Point instrument (STUART, Bibby Scientific Ltd., Roissy, France) with a precision of 1.5 °C. IR spectra were recorded with a Spectrum 100 Series FTIR spectrometer (PerkinElmer, Villebon S/Yvette, France). Liquids and solids were investigated with a single-reflection attenuated total reflectance (ATR) accessory; the absorption bands are given in cm^−1^. NMR spectra (^1^H, ^13^C and ^19^F) were acquired at 295 K using an AVANCE 300 MHz spectrometer (Bruker, Wissembourg, France) at 300, 75 and 282 MHz. Coupling constant J was in Hz and chemical shifts were given in ppm. Mass (ESI, EI and field desorption (FD) were recorded with an LCP 1er XR spectrometer (WATERS, Guyancourt, France). Mass spectrometry was performed by the Mass Spectrometry Laboratory of the University of Rouen.

### 3.2. Chemistry

#### 3.2.1. Synthesis of *N*-Arylimino-1,2,3-dithiazoles (**2**) and *N*-Arylcyanothioformamides (**3**)

All *N*-arylcyanothioformanilides **3** were obtained using a two-step procedure as described in the main text (Scheme 1). Detailed procedures and physicochemical characterization of products are available in Supplementary Materials (Sections S2–S6 for **2** series and S6–S11 for **3** series).

#### 3.2.2. Synthesis of 2-Cyanobenzothiazoles (**4**)

General procedure: To a stirred solution of *N*-arylcyanothioformamide (**3**, 0.5 mmol) in an anhydrous mixture of DMF/DMSO (1:1, *v*/*v*) (20 mL, 0.025M) were successively added PdCl_2_ (20 mol %, 17.7 mg, 0.05 mmol), CuI (50 mol %, 47.6 mg, 0.25 mmol) and KI (2.0 equiv, 166 mg, 1.0 mmol). The resulting mixture was stirred at 120 °C for 4 h after which it was diluted with AcOEt and washed with water (3 times) and brine (1 time), dried over MgSO_4_ and concentrated under reduced pressure. The crude product was purified on silica gel with PE/CH_2_Cl_2_ (50:50 to 0:100, *v*/*v*) as eluent to produce the desired product.

Some compounds of the **4** series (**4a**, **4b**, **4f**, **4o**, **4i**, **4k**, **4l**, **4n**, **4p**, **4r**, **4u** and **4af**) were randomly described in studies cited in this paper [44,47,48,51,57,69]. To complete data sometimes uneasy to find in the literature, all compounds **4** were fully characterized. The general procedure of their synthesis and physicochemical characterization are described below. ^1^H NMR and ^13^C NMR spectra of these products are available in the Supplementary Materials (Sections S12–S47).

6-Methylbenzo[*d*]thiazole-2-carbonitrile (**4a**) [51]. Brown powder (0.061 g, 70%), m.p. 92–93 °C. IR (neat) ν_max_: 2916, 2225 (CN), 1597, 1505, 1481, 1258, 1232, 1119, 1017, 821, 489, 432 cm^−1^. ^1^H NMR (300 MHz, CDCl_3_) δ 8.09 (d, *J* = 8.5 Hz, 1H), 7.76 (dt, *J* = 1.7, 0.8 Hz, 1H), 7.46 (ddd, *J* = 8.5, 1.7, 0.8 Hz, 1H), 2.56 (s, 3H). ^13^C NMR (75 MHz, CDCl_3_) δ 150.55, 139.55, 135.71, 135.32, 129.82, 124.74, 121.23, 113.17, 21.84. HRMS (EI^+^) *m*/*z*, calcd for C_9_H_7_N_2_ [M]^+^: 175.0330, found: 175.0341.

Benzo[*d*]thiazole-2-carbonitrile (**4b**) [50]. Pale yellow powder (0.051 g, 61%), m.p. 76–77 °C. IR (neat) ν_max_: 2917, 2228 (CN), 1466, 1421, 1317, 1147, 1132,759, 727, 408 cm^−1^. ^1^H NMR (300 MHz, CDCl_3_) δ 8.28–8.18 (m, 1H), 8.03–7.94 (m, 1H), 7.70–7.59 (m, 2H). ^13^C NMR (75 MHz, CDCl_3_) δ 152.38, 136.66, 135.45, 128.77, 128.07, 125.42, 121.92, 113.11. HRMS (EI^+^) *m*/*z*, calcd for C_8_H_5_N_2_S [M]^+^: 161.0185, found: 161.0173.

6-Fluorobenzo[*d*]thiazole-2-carbonitrile (**4c**) [47]. White solid (0.058 g, 65%), m.p. 108–109 °C. IR (neat) ν_max_: 3048, 2231 (CN), 1597, 1559, 1493, 1203, 1134, 820 cm^−1^. ^1^H NMR (300 MHz, CDCl_3_) δ 8.20 (ddd, *J* = 9.2, 4.8, 0.5 Hz, 1H), 7.66 (ddd, *J* = 7.7, 2.5, 0.5 Hz, 1H), 7.41 (ddd, *J* = 9.1, 8.6, 2.5 Hz, 1H). ^13^C NMR (75 MHz, CDCl_3_) δ 162.67 (d, *J* = 252.6 Hz), 149.10 (d, *J* = 1.7 Hz), 136.77 (d, *J* = 11.7 Hz), 136.38 (d, *J* = 3.8 Hz), 126.83 (d, *J* = 9.8 Hz), 117.55 (d, *J* = 25.3 Hz), 112.76, 108.05 (d, *J* = 27.4 Hz). ^19^F NMR (282 MHz, CDCl_3_) δ –109.30 (s). HRMS (EI^+^) *m*/*z*, calcd for C_8_H_4_N_2_FS [M]^+^: 179.0079, found: 179.0080.

6-Chlorobenzo[*d*]thiazole-2-carbonitrile (**4d**) [44]. White solid (0.069 g, 71%), m.p. 123–124 °C. IR (neat) ν_max_: 2228 (CN), 1467, 1311, 1147, 832, 419 cm^−1^. ^1^H NMR (300 MHz, CDCl_3_) δ 8.14 (dd, *J* = 8.8, 0.5 Hz, 1H), 7.98 (dd, *J* = 2.1, 0.5 Hz, 1H), 7.62 (dd, *J* = 8.8, 2.1 Hz, 1H). ^13^C NMR (75 MHz, CDCl_3_) δ 150.91, 137.06, 136.58, 135.41, 129.25, 126.18, 121.51, 112.73. HRMS (EI^+^) *m*/*z*, calcd for C_8_H_4_N_2_S^35^Cl [M]^+^: 194.9784, found: 197.9775.

6-Bromobenzo[*d*]thiazole-2-carbonitrile (**4e**) [69]. White solid (0.064 g, 54%), m.p. 141–142 °C. IR (neat) ν_max_: 2231 (CN), 1581, 1467, 1388, 1307, 1146, 1076, 85, 414 cm^−1^. ^1^H NMR (300 MHz, CDCl_3_) δ 8.15 (dd, *J* = 1.9, 0.5 Hz, 1H), 8.08 (dd, *J* = 8.9, 0.5 Hz, 1H), 7.76 (dd, *J* = 8.9, 1.9 Hz, 1H). ^13^C NMR (75 MHz, CDCl_3_) δ 151.13, 137.00, 136.88, 131.85, 126.35, 124.45, 123.18, 112.67. HRMS (EI^+^) *m*/*z*, calcd for C_8_H_4_N_2_S^79^Br [M]^+^: 238.9279, found: 238.9283.

6-Methoxybenzo[*d*]thiazole-2-carbonitrile (**4f**) [51]. Brownish powder (0.068 g, 71%), m.p. 123–124 °C. IR (neat) ν_max_: 2844, 2225 (CN), 1597, 1505, 1446, 1016, 821 cm^−1^. ^1^H NMR (300 MHz, CDCl_3_) δ 8.09 (dd, *J* = 9.1, 0.5 Hz, 1H), 7.36 (d, *J* = 2.5 Hz, 1H), 7.26–7.22 (dd, *J* = 9.1, 2.5 Hz, 1H), 3.93 (s, 3H). ^13^C NMR (75 MHz, CDCl_3_) δ 160.60, 147.03, 137.58, 133.46, 125.98, 118.67, 113.35, 103.10, 56.11. HRMS (EI^+^) *m*/*z*, calcd for C_9_H_7_N_2_OS [M]^+^: 191.0279, found: 191.0287.

6-Trifluoromethylbenzo[*d*]thiazole-2-carbonitrile (**4g**). Pale orange solid (0.058 g, 51%), m.p. 48–49 °C. IR (neat) ν_max_: 2228 (CN), 1475, 1318, 1160, 1124, 1077, 882, 836, 705, 650 cm^−1^. ^1^H NMR (300 MHz, CDCl_3_) δ 8.35 (dt, *J* = 8.7, 0.8 Hz, 1H), 8.32 (dt, *J* = 1.8, 0.8 Hz, 1H), 7.89 (dd, *J* = 8.7, 1.8 Hz, 1H). ^13^C NMR (75 MHz, CDCl_3_) δ 154.07, 139.76, 135.44, 130.88 (q, *J* = 33.2 Hz), 124.99 (q, *J* = 3.3 Hz), 119.87 (q, *J* = 4.3 Hz), 123.64 (q, *J* = 273.0 Hz), 112.47. ^19^F NMR (282 MHz, CDCl_3_) δ -60.43 (s). HRMS (EI^+^) *m*/*z*, calcd for C_9_H_4_N_2_F_3_S [M]^+^: 229.0047, found: 229.0057.

6-Cyanobenzo[*d*]thiazole-2-carbonitrile (**4h**). White solid (0.038 g, 41%), m.p. 178–179 °C. IR (neat) ν_max_: 3090, 2227 (CN), 1466, 1398, 1316, 1248, 1141, 834, 615, 487 cm^−1^. ^1^H NMR (300 MHz, CDCl_3_) δ 8.38–8.32 (m, 2H), 7.90 (dd, *J* = 8.6, 1.6 Hz, 1H). ^13^C NMR (75 MHz, CDCl_3_) δ 154.26, 140.71, 135.72, 130.79, 127.01, 126.41, 117.65, 112.60, 112.22. HRMS (EI^+^) *m*/*z*, calcd for C_9_H_3_N_3_S [M]^+^: 185.0048, found: 185.0045.

6-Nitrobenzo[*d*]thiazole-2-carbonitrile (**4i**). White solid (0.050 g, 51%), m.p. 166–167 °C. IR (neat) ν_max_: 3096, 2236 (CN), 1565, 1509, 1342, 1328, 1144, 1107, 902, 832, 754 cm^−1^. ^1^H NMR (300 MHz, CDCl_3_) δ 8.96 (dd, *J* = 2.2, 0.6 Hz, 1H), 8.52 (dd, *J* = 9.1, 2.2 Hz, 1H), 8.38 (dd, *J* = 9.1, 0.6 Hz, 1H). ^13^C NMR (75 MHz, CDCl_3_) δ 155.44, 147.39, 141.90, 135.71, 126.19, 123.23, 118.70, 112.13. HRMS (EI^+^) *m*/*z*, calcd for C_8_H_3_N_3_O_2_S [M]^+^: 204.9946, found: 204.9938.

Ethyl 2-cyanobenzo[*d*]thiazole-6-carboxylate (**4j**). Pale brown solid (0.065 g, 75%), m.p. 139–140 °C. IR (neat) ν_max_: 2219 (CN), 1709, 1273, 1131, 1022, 833, 768, 725, 479, 415, 389 cm^−1^. ^1^H NMR (300 MHz, CDCl_3_) δ 8.72 (dd, *J* = 1.5, 0.8 Hz, 1H), 8.31 (dd, *J* = 8.7, 1.5 Hz, 1H), 8.27 (dd, *J* = 8.7, 0.8 Hz, 1H), 4.46 (q, *J* = 7.1 Hz, 2H), 1.45 (t, *J* = 7.1 Hz, 3H). ^13^C NMR (75 MHz, CDCl_3_) δ 165.35, 154.80, 139.67, 135.32, 130.77, 128.89, 125.22, 124.11, 112.69, 62.01, 14.44. HRMS (EI^+^) *m*/*z*, calcd for C_9_H_7_N_2_OS_2_ [M]^+^: 233.0385, found: 233.0380.

5-Methoxybenzo[*d*]thiazole-2-carbonitrile (**4k**) [48]. Pale yellow powder (0.065 g, 68%), m.p. 93–94 °C. IR (neat) ν_max_: 2230 (CN), 1603, 1473, 1413, 1338, 1276, 1168, 1019, 832, 814, 470, 413 cm^−1^. ^1^H NMR (300 MHz, CDCl_3_) δ 7.82 (d, *J* = 9.0 Hz, 1H), 7.63 (d, *J* = 2.5 Hz, 1H), 7.28 (dd, *J* = 9.0, 2.5 Hz, 1H), 3.92 (s, 3H). ^13^C NMR (75 MHz, CDCl_3_) δ 160.38, 154.03, 127.45, 122.04, 120.29, 113.30, 106.24, 55.91. HRMS (EI^+^) *m*/*z*, calcd for C_9_H_7_N_2_OS_2_ [M]^+^: 191.0279, found: 191.0289.

5-Nitrobenzo[*d*]thiazole-2-carbonitrile (**4l**) [48]. White solid (0.031 g, 30%), m.p. 196–197°C. IR (neat) ν_max_: 3095, 2244 (CN), 1599, 1572, 1512, 1339, 1137, 1077, 1056, 908, 827, 738, 710, 529, 494, 411 cm^−1^. ^1^H NMR (300 MHz, DMSO-d6) δ 9.11–9.02 (m, 1H), 8.64–8.58 (m, 1H), 8.50 (dt, *J* = 9.1, 2.2 Hz, 1H). ^13^C NMR (75 MHz, DMSO-d6) δ 151.01, 147.51, 141.70, 141.67, 124.81, 122.34, 119.82, 112.88. HRMS (EI^+^) *m*/*z*, calcd for C_8_H_4_N_3_O_2_S [M]^+^: 206.0024, found: 206.0038.

Ethyl 2-cyanobenzo[*d*]thiazole-5-carboxylate (**4m**). White solid (0.066 g, 57%), m.p.: 113–114 °C. IR (neat) ν_max_: 3099, 2999, 2981, 2924, 2231 (CN), 1704, 1602, 1541, 1449, 1360, 1323, 1281, 1228, 1137, 1088, 1016, 757 cm^−1^. ^1^H NMR (300 MHz, CDCl_3_) δ 8.90 (dd, *J* = 1.6, 0.7 Hz, 1H), 8.32–8.27 (m, 1H), 8.05 (dd, *J* = 8.6, 0.6 Hz, 1H), 4.46 (q, *J* = 7.1 Hz, 2H), 1.45 (t, *J* = 7.1 Hz, 3H). ^13^C NMR (75 MHz, CDCl_3_) δ 165.56, 152.26, 139.49, 138.14, 130.94, 129.08, 126.98, 121.91, 112.71, 61.90, 14.44. HRMS (EI^+^) *m*/*z*, calcd for C_11_H_9_N_2_O_2_S [M]^+^: 233.0385, found: 233.0390.

4-Chlorobenzo[*d*]thiazole-2-carbonitrile (**4n**). White powder (0.037 g, 38%), m.p. 171–172 °C. IR (neat) ν_max_: 2227 (CN), 1579, 1543, 1457, 1314, 1148, 1095, 818, 780, 739, 646 cm^−1^. ^1^H NMR (300 MHz, CDCl_3_) δ 7.90 (dd, *J* = 8.0, 1.1 Hz, 1H), 7.69 (dd, *J* = 8.0, 1.1 Hz, 1H), 7.58 (t, *J* = 8.0 Hz, 1H). ^13^C NMR (75 MHz, CDCl_3_) δ 149.68, 137.44, 136.86, 130.64, 129.42, 128.34, 120.47, 112.64. HRMS (EI^+^) *m*/*z*, calcd for C_8_H_4_N_2_S^35^Cl [M]^+^: 194.9794, found: 197.9786.

4-Bromobenzo[*d*]thiazole-2-carbonitrile (**4o**). White powder (0.034 g, 28%), m.p. 178–179 °C. IR (neat) ν_max_: 2229 (CN), 1537, 1456, 1312, 1147, 1077, 871, 778, 738, 640 cm^−1^. ^1^H NMR (300 MHz, CDCl_3_) δ 7.94 (dd, *J* = 8.2, 1.0 Hz, 1H), 7.87 (dd, *J* = 7.7, 1.0 Hz, 1H), 7.50 (dd, *J* = 8.2, 7.7 Hz, 1H). ^13^C NMR (75 MHz, CDCl_3_) δ 150.87, 137.21, 136.29, 131.66, 129.63, 121.11, 119.25, 112.65. HRMS (EI^+^) *m*/*z*, calcd for C_8_H_4_N_2_S^79^Br [M]^+^: 238.9279, found: 238.9286.

4-Methoxybenzo[*d*]thiazole-2-carbonitrile (**4p**). Pale brown powder (0.045 g, 47%), m.p. 124–125 °C. IR (neat) ν_max_: 2225 (CN), 1559, 1475, 1457, 1330, 1270, 1193, 1129, 1035, 776, 744, 666 cm^−1^.^1^H NMR (300 MHz, CDCl_3_) δ 7.64–7.50 (m, 2H), 7.03 (dd, *J* = 7.6, 1.4 Hz, 1H). ^13^C NMR (75 MHz, CDCl_3_) δ 154.99, 143.18, 137.33, 134.93, 130.29, 113.48, 113.06, 107.84, 56.47. HRMS (EI^+^) *m*/*z*, calcd for C_9_H_7_N_2_OS [M]^+^: 191.0279, found: 191.0277.

5,6-Dimethylbenzo[*d*]thiazole-2-carbonitrile (**4q**). Pale brown solid (0.090 g, 96%), m.p. 136–137 °C. IR (neat) ν_max_: 3045, 2980, 2949, 2924, 2228 (CN), 1610, 1432, 1261, 1149, 865, 429 cm^−1^. ^1^H NMR (300 MHz, CDCl_3_) δ 7.96 (d, *J* = 1.0 Hz, 1H), 7.71 (d, *J* = 1.0 Hz, 1H), 2.44 (s, 6H). ^13^C NMR (75 MHz, CDCl_3_) δ 151.28, 139.26, 137.93, 135.07, 133.14, 124.98, 121.38, 113.41, 77.58, 77.16, 76.74, 20.63, 20.37. HRMS (EI^+^) *m*/*z*, calcd for C_10_H_9_N_2_S [M]^+^: 189.0486, found: 189.0485.

5,6-Dimethoxybenzo[*d*]thiazole-2-carbonitrile (**4r**) [57]. Pale brown solid (0.104 g, 94%), m.p. 160–161 °C. IR (neat) ν_max_: 2222 (CN), 1494, 1439, 1418, 1282, 1230, 1207, 1170, 1059, 846, 813 cm^−1^. ^1^H NMR (300 MHz, CDCl_3_) δ 7.56 (s, 1H), 7.29 (s, 1H), 3.99 (s, 3H), 3.98 (s, 3H) ^13^C NMR (75 MHz, CDCl_3_) δ 151.84, 151.10, 147.29, 133.47, 128.84, 113.49, 105.31, 101.37, 56.59, 56.39. HRMS (EI^+^) *m*/*z*, calcd for C_10_H_9_N_2_O_2_S [M]^+^: 221.0385, found: 221.0395.

6-Bromo-5-methylbenzo[*d*]thiazole-2-carbonitrile (**4s**). White solid (0.090 g, 71%), m.p. 182–183 °C. IR (neat) ν_max_: 3055, 2957, 2920, 2229 (CN), 2116, 1770, 1689, 1519, 1420, 1297, 1168, 1138, 887, 834, 419 cm^−1^. ^1^H NMR (300 MHz, CDCl_3_) δ 8.18 (s, 1H), 8.08 (d, *J* = 1.1 Hz, 1H), 2.59 (d, *J* = 0.9 Hz, 3H). ^13^C NMR (75 MHz, CDCl_3_) δ 151.94, 138.68, 136.94, 134.03, 126.59, 126.09, 124.80, 112.90, 23.63. HRMS (EI^+^) *m*/*z*, calcd for C_9_H_6_N_2_S^79^Br [M]^+^: 252.9435, found: 252.9446.

[1,3]Dioxolo [4′,5′:4,5]benzo [1,2-*d*]thiazole-6-carbonitrile (**4t**). White solid (0.068 g, 67%), m.p. 190–191 °C. IR (neat) ν_max_: 3038, 2917, 2225 (CN), 1472, 1427, 1274, 1144, 1031, 937, 869, 797, 485, 414 cm^−1^. ^1^H NMR (300 MHz, CDCl_3_) δ 7.53 (s, 1H), 7.28 (s, 1H), 6.15 (s, 2H). ^13^C NMR (75 MHz, CDCl_3_) δ 150.40, 149.97, 148.20, 134.01, 130.24, 113.31, 104.72, 102.88, 98.32. HRMS (EI^+^) *m*/*z*, calcd for C_9_H_5_N_2_O_2_S [M]^+^: 205.0072, found: 205.0067.

6,7-Dihydro-[1,4]dioxino [2′,3′:4,5]benzo [1,2-*d*]thiazole-2-carbonitrile (**4u**). Pale brown solid (0.094 g, 86%), m.p. 179–180 °C. IR (neat) ν_max_: 2230 (CN), 1481, 1302, 1176, 1063, 928, 873, 809, 709 cm^−1^.^1^H NMR (300 MHz, CDCl_3_) δ 7.66 (s, 1H), 7.37 (s, 1H), 4.36 (m, 4H). ^13^C NMR (75 MHz, CDCl_3_) δ 147.53, 146.42, 145.27, 134.60, 129.03, 113.35, 111.93, 108.15, 64.62, 64.18. HRMS (EI^+^) *m*/*z*, calcd for C_10_H_7_N_2_O_2_S [M]^+^: 219.0228, found: 219.0220.

4,5-dimethylbenzo[*d*]thiazole-2-carbonitrile (**4v**). White solid (0.035 g, 37%), m.p. 93–94 °C. IR (neat) ν_max_: 3667, 2988, 2230 (CN), 1551, 1461, 1067, 880, 808, 565 cm^−1^. ^1^H NMR (300 MHz, CDCl_3_) δ 7.68 (d, *J* = 8.3 Hz, 1H), 7.42 (d, *J* = 8.3 Hz, 1H), 2.72 (s, 3H), 2.46 (s, 3H). ^13^C NMR (75 MHz, CDCl_3_) δ 152.56, 136.11, 135.01, 133.86, 132.86, 131.28, 118.28, 113.54, 19.71, 15.02. HRMS (EI^+^) *m*/*z*, calcd for C_10_H_9_N_2_S [M]^+^: 189.0486, found: 189.0499.

4,5-Dichlorobenzo[*d*]thiazole-2-carbonitrile (**4w**). White powder (0.056 g, 49%), m.p. 171–172 °C. IR (neat) ν_max_: 3070, 2232 (CN), 1527, 1461, 1435, 1384, 1303, 1232, 1186, 1155, 1101, 927, 814, 676, 611, 573 cm^−1^. ^1^H NMR (300 MHz, CDCl_3_) δ 7.83 (d, *J* = 8.7 Hz, 1H), 7.72 (d, *J* = 8.7 Hz, 1H). ^13^C NMR (75 MHz, CDCl_3_) δ 150.87, 138.74, 134.70, 133.22, 130.56, 129.25, 120.29, 112.34. HRMS (EI^+^) *m*/*z*, calcd for C_8_H_3_N_2_S^35^Cl_2_ [M]^+^: 228.9394, found: 228.9393.

5-Chloro-4-methylbenzo[*d*]thiazole-2-carbonitrile (**4x**). White powder (0.033 g, 32%), m.p. 129–130 °C. IR (neat) ν_max_: 2232 (CN), 1553, 1450, 1377, 1308, 1197, 1155, 1119, 1017, 807 cm^−1^. ^1^H NMR (300 MHz, CDCl_3_) δ 7.74 (dq, *J* = 8.7, 0.7 Hz, 1H), 7.61 (dd, *J* = 8.7, 0.7 Hz, 1H), 2.84 (s, 3H). ^13^C NMR (75 MHz, CDCl_3_) δ 152.99, 136.76, 134.13, 133.98, 133.67, 129.93, 119.43, 113.00, 15.96. HRMS (EI^+^) *m*/*z*, calcd for C_9_H_6_N_2_S^35^Cl [M]^+^: 208.9940, found: 208.9943.

4,6-Difluorobenzo[*d*]thiazole-2-carbonitrile (**4y**) [47]. White powder (0.034 g, 35%), m.p. 100–101°C. IR (neat) ν_max_: 1620, 1519, 1471, 1422, 1288, 1258, 1118, 855, 839, 539 cm^−1^. ^1^H NMR (300 MHz, CDCl_3_) δ 7.50 (ddd, *J* = 7.4, 2.3, 1.3 Hz, 1H), 7.17 (ddd, *J* = 9.7, 9.0, 2.3 Hz, 1H). ^19^F NMR (282 MHz, CDCl_3_) δ -104.97 (d, *J* = 8.2 Hz), -113.01 (d, *J* = 8.2 Hz). ^13^C NMR (75 MHz, CDCl_3_) δ 165.23–153.86 (m), 139.12–137.94 (m), 136.60 (d, *J* = 3.6 Hz), 126.77, 117.73, 112.25, 108.06 (d, *J* = 27.1 Hz), 105.72–102.58 (m). HRMS (EI^+^) *m*/*z*, calcd for C_8_H_3_N_2_F_2_S [M]^+^: 196.9985, found: 196.9992.

4,6-Dimethoxybenzo[*d*]thiazole-2-carbonitrile (**4z**). White powder (0.072 g, 65%), m.p. 140–141 °C. IR (neat) ν_max_: 2979, 2223 (CN), 1598, 1572, 1476, 1452, 1290, 1218, 1165, 1039, 819, 813, 799 cm^−1^. ^1^H NMR (300 MHz, CDCl_3_) δ 6.92 (d, *J* = 2.1 Hz, 1H), 6.61 (d, *J* = 2.1 Hz, 1H), 4.04 (s, 3H), 3.91 (s, 3H). ^13^C NMR (75 MHz, CDCl_3_) δ 162.30, 155.18, 138.88, 138.47, 131.59, 113.28, 99.51, 94.42, 56.47, 56.17. HRMS (EI^+^) *m/z*, calcd for C_10_H_9_N_2_O_2_S [M]^+^: 221.0385, found: 221.0390.

4-Fluoro-6-methoxybenzo[*d*]thiazole-2-carbonitrile (**4aa**). Pale brown solid (0.067 g, 64%), m.p.: 153–154 °C. IR (neat) ν_max_: 2916, 2847, 2226 (CN), 1615, 1566, 1476, 1443, 1290, 1132, 1027, 858, 826, 572 cm^−1^. ^1^H NMR (300 MHz, CDCl_3_) δ 7.16 (dd, *J* = 2.3, 0.8 Hz, 1H), 6.96 (dd, *J* = 11.4, 2.3 Hz, 1H), 3.92 (s, 3H). ^13^C NMR (75 MHz, CDCl_3_) δ 161.72 (d, *J* = 10.2 Hz), 156.83 (d, *J* = 261.5 Hz), 139.19 (d, *J* = 4.2 Hz), 136.77 (d, *J* = 15.0 Hz), 133.73, 112.82, 104.37 (d, *J* = 20.3 Hz), 99.35 (d, *J* = 4.0 Hz), 56.51. ^19^F NMR (282 MHz, CDCl_3_) δ −116.73. HRMS (EI^+^) *m*/*z*, calcd for C_9_H_6_N_2_OFS [M]^+^: 209.0185, found: 209.0193.

5,7-Dimethylbenzo[*d*]thiazole-2-carbonitrile (**4ab**). White powder (0.056 g, 59%), m.p. 90–91 °C. IR (neat) ν_max_: 2921, 2232 (CN), 1556, 1459, 1378, 1289, 1145, 1124, 1037, 872, 689, 611, 563, 481 cm^−1^.^1^H NMR (300 MHz, CDCl_3_) δ 7.82 (dt, *J* = 1.6, 0.8 Hz, 1H), 7.24 (dt, *J* = 1.6, 0.8 Hz, 1H), 2.56 (d, *J* = 0.8 Hz, 3H), 2.51 (s, 3H). ^13^C NMR (75 MHz, CDCl_3_) δ 152.74, 138.71, 135.90, 133.39, 131.51, 130.62, 122.43, 113.36, 21.51, 21.30. HRMS (EI^+^) *m*/*z*, calcd for C_10_H_9_N_2_S [M]^+^: 189.0486, found: 189.0471.

5,7-Dimethoxybenzo[*d*]thiazole-2-carbonitrile (**4ac**). White powder (0.072 g, 65%), m.p. 179–180 °C. IR (neat) ν_max_: 3085, 2979, 2947, 2224 (CN), 1601, 1571, 1407, 1303, 1158, 1125, 9354, 833, 819, 498 cm^−1^. ^1^H NMR (300 MHz, CDCl_3_) δ 7.23 (d, *J* = 2.0 Hz, 1H), 6.65 (d, *J* = 2.0 Hz, 1H), 3.98 (s, 3H), 3.91 (s, 3H). ^13^C NMR (75 MHz, CDCl_3_) δ 161.80, 154.46, 154.25, 137.37, 117.97, 113.34, 100.19, 98.18, 56.37, 56.09. HRMS (EI^+^) *m*/*z*, calcd for C_10_H_9_N_2_O_2_S [M]^+^: 221.0385, found: 221.0394.

5-methyl-7-bromobenzo[*d*]thiazole-2-carbonitrile and 5-bromo-7-methylbenzo[*d*]thiazole-2-carbonitrile (**4ad’** + **4ad’’**) **4ad’**: ^1^H NMR (300 MHz, CDCl_3_) δ 8.25–8.19 (m, 1H), 7.55 (dd, *J* = 1.8, 0.9 Hz, 1H), 2.61 (t, *J* = 0.8 Hz, 3H). **4ad’’**: ^1^H NMR (300 MHz, CDCl_3_) δ 7.95 (dd, *J* = 1.4, 0.6 Hz, 1H), 7.60 (dd, *J* = 1.4, 0.6 Hz, 1H), 2.55 (t, *J* = 0.6 Hz, 3H).

5-Methoxy-7-bromobenzo[*d*]thiazole-2-carbonitrile (**4ae’**). White powder (0.014 g, 11%), m.p. 170–171°C. IR (neat) ν_max_: 2235 (CN), 1590, 1537, 1464, 1448, 1394, 1274, 1158, 1086, 1024, 983, 841, 722, 633, 480, 423 cm^−1^. ^1^H NMR (300 MHz, CDCl_3_) δ 7.57 (d, *J* = 2.2 Hz, 1H), 7.42 (d, *J* = 2.2 Hz, 1H), 3.91 (s, 3H). ^13^C NMR (75 MHz, CDCl_3_) δ 160.86, 153.01, 137.57, 130.83, 122.42, 113.75, 112.87, 105.85, 56.27. HRMS (EI^+^) *m*/*z*, calcd for C_9_H_5_N_2_OS^79^Br [M]^+^: 267.9306, found: 267.9309.

5-Bromo-7-methoxybenzo[*d*]thiazole-2-carbonitrile (**4ae’’**). White powder (0.029 g, 24%), m.p. 170–171°C. IR (neat) ν_max_: 3071, 2931, 2233 (CN), 1554, 1451, 1278, 1121, 972, 863, 836, 565, 385 cm^−1^. ^1^H NMR (300 MHz, CDCl_3_) δ 7.99 (d, *J* = 1.5 Hz, 1H), 7.11 (d, *J* = 1.5 Hz, 1H), 4.03 (s, 3H). ^13^C NMR (75 MHz, CDCl_3_) δ 154.33, 154.15, 138.43, 123.91, 122.48, 120.40, 112.77, 111.79, 56.76. HRMS (EI^+^) *m*/*z*, calcd for C_9_H_5_N_2_OS^79^Br [M]^+^: 267.9306, found: 267.9304.

## 4. Conclusions

We have investigated reaction conditions involving palladium and copper to achieve the successful cyclization of cyanothioformamides (**3**), leading to benzothiazoles **4** substituted in various positions and bearing in position C2 the versatile carbonitrile function. In this process, the presence of 2.0 equiv of an inorganic additive such as KI proved to be essential for a better conversion. The presence of air was also found to be crucial to the reaction, allowing reoxidation of Pd^0^ at the end of the process. In many cases, the selective C-H functionalization/C-S bond formation reactions were performed in good to very good yields, allowing a wide range of benzothiazole derivatives. In comparison with previous work, this synthetic route produced only one regioisomer, except in the case of unsymmetrical 3,5-disubstituted thioformanilides wherein steric effects due to substituents may influence the reaction outcome. Moreover, this work allowed the formation of an array of polyfunctionalized 2-cyanobenthiazoles, as building blocks for the construction of more complex heterocyclic systems or potent applications in molecular labeling.

## Figures and Tables

**Table 1:** Preliminary exploration of reaction conditions.

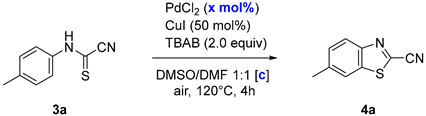			
Entry	PdCl_2_ (x mol%)	Solvent [c] (M)	Yield (%) ^1^
1	10	0.050	40
2	10	0.025	44
3	20	0.050	46
4	20	0.025	49 ^2^

^1^ Isolated yields. ^2^ 51% with TBAI in place of TBAB.

**Table 2:** Effect of inorganic additives and ambient atmosphere.

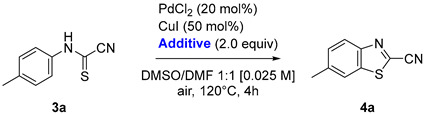					
Entry	Additive	Yield (%) ^1^	Entry	Additive	Yield (%) ^1^
1	CsF	15	6	KF	16
2	CsI	32	7	LiBr	53
3	NaI	53	8	LiCl	33
4	KBr	66	9	LiI	55
5	KI	70 ^2,3,4,5^	10	none	37

^1^ Isolated yields. ^2^ 23% when performed under inert atmosphere (argon). ^3^ 39% with 3.0 equiv of KI. ^4^ Replacing KI with a base of K_2_CO_3_ or LiOtBu did not provide successful results. ^5^ Adding a ligand (50 mol%) including phenantroline or L-proline gave 0% and 7% yields of **4a**, respectively.

**Table 3:** Optimization of the palladium and copper sources as well as the solvent.

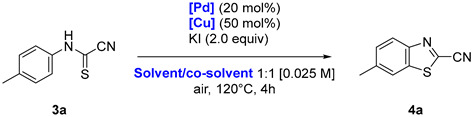				
Entry	[Pd]	[Cu]	Solvent/Co-Solvent	Yield (%) ^1^
1	PdCl_2_	CuI	DMSO/DMF	70
2	PdBr_2_	CuI	DMSO/DMF	67
3	Pd(OAc)_2_	CuI	DMSO/DMF	22
4	Pd_2_dba_3_	CuI	DMSO/DMF	7
5	none	CuI	DMSO/DMF	0
6	PdCl_2_	CuBr	DMSO/DMF	53
7	PdCl_2_	CuCl_2_	DMSO/DMF	57
8	PdCl_2_	Cu(OAc)_2_	DMSO/DMF	39
9	PdCl_2_	none	DMSO/DMF	41
10	PdCl_2_	CuI	DMSO/NMP	18
11	PdCl_2_	CuI	DMSO/-	34
12	PdCl_2_	CuI	DMF/-	14

^1^ Isolated yields.

**Table 4:** Optimization of PdCl_2_ and CuI sources as well as starting molar concentration.

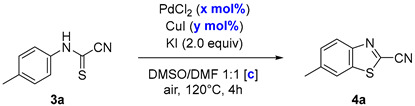				
Entry	PdCl_2_ (x mol%)	CuI (y mol%)	[c] M	Yield (%) ^1^
1	20	50	0.025	70
2	20	50	0.050	53
3	20	20	0.025	51
4	10	50	0.025	21
5	10	20	0.025	14

^1^ Isolated yields.

**Table 5:** Synthesis of dithiazoles **2** and cyanothioformanilides **3** from anilines **1**.

Aniline	R	Yield of 2 (%) ^1^	Yield of 3 (%) ^1^	Aniline	R	Yield of 2 (%) ^1^	Yield of 3 (%) ^1^
**1a**	4-Me	67 ^2^	46 ^6^	**1r**	3,4-diOMe	61 ^8^	71 ^6^
**1b**	H	59 ^2^	41 ^6^	**1s**	3-Me, 4-Br	84	63
**1c**	4-F	86 ^3^	47 ^6^	**1t**	3,4(-OCH_2_O-)	26 ^9^	55
**1d**	4-Cl	82 ^2^	45 ^6^	**1u**	3,4(-OCH_2_CH_2_O-)	65 ^10^	71
**1e**	4-Br	86 ^4^	47	**1v**	2,3-diMe	64	79
**1f**	4-OMe	48 ^2^	33 ^6^	**1w**	2,3-diCl	87	78 ^6^
**1g**	4-CF_3_	72	56 ^6^	**1x**	2-Me, 3-Cl	77	85
**1h**	4-CN	69 ^5^	71	**1y**	2,4-diF	94 ^3^	64 ^6^
**1i**	4-NO_2_	85	32 ^6^	**1z**	2,4-diOMe	75 ^5^	75 ^6^
**1j**	4-CO_2_Et	78	82 ^6^	**1aa**	2-F, 4-OMe	92	75
**1k**	3-OMe	74 ^2^	68 ^6^	**1ab**	3,5-diMe	91	77
**1l**	3-NO_2_	90	81 ^6^	**1ac**	3,5-diOMe	45	65
**1m**	3-CO_2_Et	85	75	**1ad**	3-Br, 5-Me	63	44
**1n**	2-Cl	89	83	**1ae**	3-Br, 5-OMe	95	85
**1o**	2-Br	85 ^2^	80 ^6^	**1af**	2,5-diMe	69 ^4^	77
**1p**	2-OMe	90 ^7^	79	**1ag**	2-Me, 5-iPr	76 ^5^	78
**1q**	3,4-diMe	88	52	**-**	-	-	-

^1^ Isolated yield. Compounds described in ^2^ [48]; ^3^ [47]; ^4^ [50]; ^5^ [64]; ^6^ [57]; ^7^ [17]; ^8^ [65]; ^9^ [66]; ^10^ [67].

## Data Availability

Not applicable.

## Supplementary Materials

The following supporting information can be downloaded at: https://www.mdpi.com/article/10.3390/molecules27238426/s1: Synthesis of detailed procedures and physicochemical characterization of products *N*-arylimino-1,2,3-dithiazoles (**2**) and *N*-arylcyanothioformamides (**3**) (Sections S2–S11). ^1^H NMR and ^13^C NMR spectra of compounds **4a**–**z** and **4aa**-**4ag** (Sections S12–S47).

## Sample Availability

Samples of the compounds are not available from the authors.
