# Supplementary material for: Synthesis of 2-Cyanobenzothiazoles via Pd-Catalyzed/Cu-Assisted C-H Functionalization/Intramolecular C-S Bond Formation from N-Arylcyanothioformamides
Source: Molecules. 2022 Dec 1;27(23):8426. doi: 10.3390/molecules27238426 (PMC9738468; doi:10.3390/molecules27238426)
Supplement: Supplementary file 1 [file molecules-27-08426-s001.zip › molecules-2057792-supplementary.pdf]

molecules

Supplementary Materials

Article

Synthesis of 2-cyanobenzothiazoles via Pd-catalyzed/ Cu-assisted C-H functionalization/intramolecular C-S bond formation from *N*-arylcyanothioformamides

Nathan Broudic [1], Alexandra Pacheco-Benichou [1], Corinne Fruit [1,*] and Thierry Besson [1,*]

Univ Rouen Normandie, INSA Rouen, CNRS UMR 6014 COBRA, F-76000 Rouen, France

* Correspondence: corinne.fruit@univ-rouen.fr (CF); thierry.besson@univ-rouen.fr (TB)

Table of contents

Pages

1. General information S2
2. Synthesis of *N*-arylimino-1,2,3-dithiazoles (**2**) and *N*-arylcyanothioformamides (**3**). S2

   2.1 Synthesis of N-(4-chloro-5*H*-1,2,3-dithiazol-5-ylidene) anilines (**2**). S2-S6

   62.2 General procedure for the synthesis of *N*-arylcyanothioformamides (**3**) S6-S11
3. ^{1}H, ^{13}C and ^{19}F NMR Spectra of 2-cyanobenzothiazoles (**4a-z** and **4aa-ag**) S12-47

1. **General information**

All reagents were purchased from commercial suppliers and were used without further purification.

All reactions were monitored by thin-layer chromatography with aluminium plates (0.25 mm) precoated with silica gel 60 F254 (Merck KGaA, Darmstadt, Germany). Visualization was performed with UV light at a wavelength of 254 nm.

Purifications were conducted with a flash column chromatography system (PuriFlash, Interchim, Montluçon, France) using stepwise gradients of petroleum ether (also called light petroleum) (PE), and dichloromethane (DCM) as the eluent.

Melting points were measured with an SMP3 Melting Point instrument (STUART, Bibby Scientific Ltd., Roissy, France) with a precision of 1.5 °C.

IR spectra were recorded with a Spectrum 100 Series FTIR spectrometer (PerkinElmer, Villebon S/Yvette, France). Liquids and solids were investigated with a single-reflection attenuated total reflectance (ATR) accessory; the absorption bands are given in cm^{-1}.

NMR spectra (^1H, ^{13}C and ^{19}F) were acquired at 295 K using an AVANCE 300 MHz spectrometer (Bruker, Wissembourg, France) at 300, 75 and 282 MHz. Coupling constant J was in Hz and chemical shifts were given in ppm.

Mass (ESI, EI and field desorption (FD) were recorded with an LCP 1er XR spectrometer (WATERS, Guyancourt, France). Mass spectrometry was performed by the Mass Spectrometry Laboratory of the University of Rouen.

2. **Synthesis of *N*-arylimino-1,2,3-dithiazoles (2) and N-arylcyanothioformamides (3).**

All *N*-arylcyanothioformanilides **3** were obtained using a two-step procedure. Anilines **1** were stirred with Appel salt (1.1 equiv) and pyridine (2.0 equiv) in dichloromethane (DCM) at r.t. for 1 h to give the corresponding imino-1,2,3-dithiazoles 2. Compounds **2** were then treated by 3 equiv of 1,8-diazabicyclo[5.4.0]undéc-7-ene (DBU) in DCM at r.t. for 15 min. Detailed procedures and physicochemical characterization of products are described below.

2.1 Synthesis of *N*-(4-chloro-5*H*-1,2,3-dithiazol-5-ylidene) anilines (**2**).

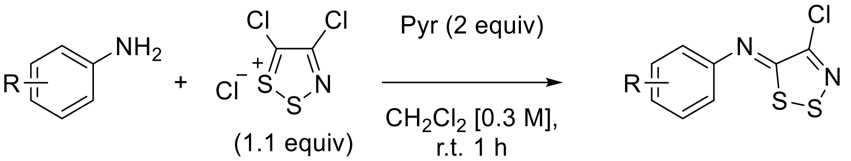

1 **Appel salt** **2**

To a stirred solution of aniline (**1**, 4.0 mmol) in CH_2Cl_2 (13.3 mL) were successively added Appel Salt (0.917 g, 4.4 mmol) and pyridine (0.644 mL, 8.0 mmol). The reaction mixture was stirred at room temperature for 1h after which water (10 mL) was added. The resulting emulsion was extracted with CH_2Cl_2. The organic phase was washed with brine, dried over $MgSO_4$ and concentrated under reduced pressure. The crude product was purified on silica gel with petroleum ether (PE)/CH_2Cl_2 (100:0 to 0:100, v/v) as eluent to afford the desired product.

Some compounds of the **2** and **3** series were randomly described in academic works cited in the main text [17,47,48,50,57, 67, 68 and 69], in these cases physicochemical characterization was limited to m.p. (°C), IR (cm^{-1}) and ^1H NMR (300 MHz, $CDCl_3$). All new compounds are fully described below.

N-(4-Chloro-5*H*-1,2,3-dithiazol-5-ylidene)-4-methyl-aniline (**2a**) [48]. Dark orange solid (0.249 g, 67%). m.p. 75-76 °C. IR (neat) ν_{max}: 2912, 2337, 2116, 1891, 2089, 1497, 1134, 858 cm^{-1}.^{1}H NMR (300 MHz, $CDCl_3$) δ 7.32 – 7.25 (m, 2H), 7.21 – 7.14 (m, 2H), 2.41 (s, 3H).

N-(4-Chloro-5*H*-1,2,3-dithiazol-5-ylidene)aniline (**2b**) [48]. Dark brown oil (0.546 g, 59%). IR (neat) ν_{max}: 3057, 1690, 1573, 1481, 1446, 1216, 1136, 905, 855, 757, 690 cm^{-1}.^{1}H NMR (300 MHz, $CDCl_3$) δ 7.42 – 7.33 (m, 2H), 7.21 – 7.09 (m, 3H).

N-(4-Chloro-5*H*-1,2,3-dithiazol-5-ylidene)-4-fluoro-aniline (**2c**) [47]. Dark orange solid (0.697 g, 71%), m.p. 71-72 °C. IR (neat) νmax: 3058, 2357, 1578, 1494, 1207, 1130, 855, 793 cm^{-1}.^{1}H NMR (300 MHz, $CDCl_3$) δ 7.31 – 7.12 (m, 4H). ^{19}F NMR (282 MHz, $CDCl_3$) δ -114.76 (s).

N-(4-Chloro-5*H*-1,2,3-dithiazol-5-ylidene)-4-chloro-aniline (**2d**). Orange solid (0.867 g, 82%), m.p. 110-111 °C. IR (neat) ν_{max}: 1593, 1577, 1556, 1484, 1399, 1277, 1218, 1134, 1091, 1010, 858, 826, 779, 695, 607, 537, 450 cm^{-1}. ^{1}H NMR (300 MHz, $CDCl_3$) δ 7.47 – 7.39 (m, 2H), 7.20 – 7.13 (m, 2H). ^{13}C NMR (75 MHz, $CDCl_3$) δ 159.15, 149.53, 148.09, 131.89, 130.14 (2C), 121.14 (2C). HRMS (EI^{+}) m/z, calcd for $C_8H_5N_2S_2{}^{35}Cl_2$ [M]$^{+}$: 262.9271, found: 262.9271.

N-(4-Chloro-5*H*-1,2,3-dithiazol-5-ylidene)-4-bromo-aniline (**2e**) [50]. Brown solid (1.05 g, 86%), m.p. 109-110°C. IR (neat) ν_{max}: 1594, 1478, 1216, 1069, 860, 825, 778, 520, 444, 407 cm^{-1}. ^{1}H NMR (300 MHz, $CDCl_3$) δ 7.63 – 7.53 (m, 2H), 7.16 – 7.05 (m, 2H).

N-(4-Chloro-5*H*-1,2,3-dithiazol-5-ylidene)-4-methoxy-aniline (**2f**) [48]. Yellow powder (0.492 g, 48%), m.p. 96-97 °C. IR (neat) ν_{max}: 2967, 2836, 2115, 2089, 1903, 1603, 1502, 1250, 1126, 1019, 826 cm^{-1}. ^{1}H NMR (300 MHz, $CDCl_3$) δ 7.27 – 7.15 (m, 2H), 6.97 – 6.86 (m, 2H), 3.78 (s, 3H).

N-(4-Chloro-5*H*-1,2,3-dithiazol-5-ylidene)-4-trifluoromethyl-aniline (**2g**). Dark yellow solid (0.850 g, 72%), m.p. 59-60 °C. IR (neat) ν_{max}: 1588, 1323, 1119, 1066, 863, 755, 523, 399, 387 cm^{-1}. ^{1}H NMR (300 MHz, $CDCl_3$) δ 7.76 – 7.68 (m, 2H), 7.31 – 7.23 (m, 2H). ^{13}C NMR (75 MHz, $CDCl_3$) δ 160.54, 154.28, 147.78, 127.99 (q, *J* = 33.0 Hz), 127.31 (q, *J* = 3.8 Hz), 125.76, 122.15, 119.56. ^{19}F NMR (282 MHz, $CDCl_3$) δ –62.24 (s). HRMS (EI^{+}) m/z, calcd for $C_9H_5N_2F_3S_2{}^{35}Cl$ [M]$^{+}$: 296.9535, found: 296.9561.

N-4-[(4-Chloro-5*H*-1,2,3-dithiazol-5-ylidene)amino]benzonitrile (**2h**) [66]. Yellow solid (0.704 g, 69%), m.p. 155-156 °C. IR (neat) ν_{max}: 2229 (CN), 1571, 1493, 1237, 1179, 873, 833, 189, 738, 560 cm^{-1}. ^{1}H NMR (300 MHz, $CDCl_3$) δ 7.80 – 7.70 (m, 2H), 7.31 – 7.20 (m, 2H).

N-(4-Chloro-5*H*-1,2,3-dithiazol-5-ylidene)-4-nitro-aniline (**2i**). Orange powder (0.930 g, 85%), m.p. 169-170 °C. IR (neat) ν_{max}: 1574, 1554, 1497, 1334, 1226, 1143, 1137, 1104, 867, 783, 753, 609, 538, 511, 436 cm^{-1}. ^{1}H NMR (300 MHz, $CDCl_3$) δ 8.47 – 8.23 (m, 2H), 7.32 – 7.26 (m, 2H). ^{13}C NMR (75 MHz, $CDCl_3$) δ 161.42, 156.86, 147.85, 145.53, 126.14, 120.09. HRMS (EI^{+}) m/z, calcd for $C_8H_5N_3O_2S_2{}^{35}Cl$ [M]$^{+}$: 273.9512, found: 273.9514.

Ethyl 4-[(4-chloro-5*H*-1,2,3-dithiazol-5-ylidene)amino]benzoate (**2j**). Orange powder (0.939 g, 78%), m.p. 127-128°C. IR (neat) ν_{max}: 2991, 1698, 1585, 1289, 871, 769, 699, 399, 390 cm^{-1}. ^{1}H NMR (300 MHz, $CDCl_3$) δ 8.20 – 8.09 (m, 2H), 7.25 – 7.17 (m, 2H), 4.39 (q, *J* = 7.1 Hz, 2H), 1.41 (t, *J* = 7.1 Hz, 3H). ^{13}C NMR (75 MHz, $CDCl_3$) δ 166.05, 160.24, 155.31, 147.95, 131.81 (2C), 128.43, 119.26 (2C), 61.23, 14.50. HRMS (EI^{+}) m/z, calcd for $C_{11}H_{10}N_2OS_2{}^{35}Cl$ [M]$^{+}$: 300.9872, found: 300.9880.

N-(4-Chloro-5*H*-1,2,3-dithiazol-5-ylidene)-3-methoxy-aniline (**2k**) [48]. Dark orange oil (0.768 g, 74%). IR (neat) ν_{max}: 3065, 3000, 2936, 2832, 2742, 1570, 1477, 1262, 1192, 1136, 857 cm^{-1}. ^{1}H NMR (300 MHz, $CDCl_3$) δ 7.42 – 7.31 (m, 1H), 6.85 – 6.77 (m, 2H), 6.77 – 6.72 (m, 1H), 3.83 (s, 3H).

N-(4-Chloro-5*H*-1,2,3-dithiazol-5-ylidene)-3-nitro-aniline (**2l**). Yellow powder (0.990 g, 90%), m.p. 137-138 °C. IR (neat) ν_{max}: 3065, 1575, 1514, 1500, 1314, 1224, 1152, 895, 865, 827, 797, 781, 773, 702, 677, 502, 441 cm^{-1}. ^{1}H NMR (300 MHz, $CDCl_3$) δ 8.16 – 8.04 (m, 2H), 7.65 (td, *J* = 7.9, 0.7 Hz, 1H), 7.52 (ddd, *J* = 8.0, 2.0, 1.1 Hz, 1H). ^{13}C NMR (75 MHz, $CDCl_3$) δ 161.26, 152.19, 149.48, 147.97, 131.06, 126.17, 121.09, 114.63. HRMS (EI^+) m/z, calcd for $C_8H_5N_3O_2S_2{}^{35}Cl$ $[M]^+$: 273.9512, found: 273.9510.

Ethyl 3-[(4-chloro-5*H*-1,2,3-dithiazol-5-ylidene)amino]benzoate (**2m**). Yellow powder (1.02 g, 85%), m.p. 62-63 °C. IR (neat) ν_{max}: 2978, 2902, 1713, 1586, 1474, 1367, 1292, 1286, 1203, 1144, 1099, 1075, 1016, 853, 772, 677, 660 cm^{-1}. ^{1}H NMR (300 MHz, $CDCl_3$) δ 7.96 – 7.88 (m, 2H), 7.54 (t, J = 8.0 Hz, 1H), 7.38 (ddd, J = 8.0, 2.2, 1.1 Hz, 1H), 4.39 (q, J = 7.1 Hz, 2H), 1.40 (t, J = 7.1 Hz, 3H). ^{13}C NMR (75 MHz, $CDCl_3$) δ 166.03, 159.88, 151.46, 148.27, 132.47, 130.16, 127.63, 124.26, 120.43, 77.58, 77.16, 76.74, 61.43, 14.46. HRMS (EI^+) m/z, calcd for $C_{11}H_{10}N_2O_2S_2{}^{35}Cl$ $[M]^+$: 300.9872, found: 300.9863.

N-(4-Chloro-5*H*-1,2,3-dithiazol-5-ylidene)-2-chloro-aniline (**2n**). Orange solid (0.940 g, 89%), m.p. 78-79°C. IR (neat) ν_{max}: 1598, 1464, 1143, 862, 780, 749 cm^{-1}. ^{1}H NMR (300 MHz, $CDCl_3$) δ 7.50 (dd, *J* = 8.0, 1.4 Hz, 1H), 7.34 (td, *J* = 7.6, 1.4 Hz, 1H), 7.17 (td, *J* = 7.6, 1.6 Hz, 2H), 7.12 (dd, *J* = 8.0, 1.6 Hz, 1H). ^{13}C NMR (75 MHz, $CDCl_3$) δ 161.47, 149.01, 147.62, 130.91, 128.38, 127.16, 125.52, 118.65. HRMS (EI^+) m/z, calcd for $C_8H_5N_2S_2{}^{35}Cl_2$ $[M]^+$: 262.9271, found: 262.9274.

N-(4-Chloro-5*H*-1,2,3-dithiazol-5-ylidene)-2-bromo-aniline (**2o**) [48]. Dark orange solid (1.04 g, 85%), m.p. 79-80 °C. IR (neat) ν_{max}: 1599, 1463, 1223, 1151, 1025, 875, 856, 752 cm^{-1}. ^{1}H NMR (300 MHz, $CDCl_3$) δ 7.69 (ddd, *J* = 7.8, 1.4, 0.6 Hz, 1H), 7.39 (m, 1H), 7.14 – 7.07 (m, 2H).

N-(4-Chloro-5*H*-1,2,3-dithiazol-5-ylidene)-2-methoxy-aniline (**2p**) [17]. Dark orange solid (0.935 g, 90%), m.p. 69-70°C. IR (neat) ν_{max}: 2961, 2835, 1603, 1484, 1249, 1157, 1112, 1021, 753 cm^{-1}. ^{1}H NMR (300 MHz, $CDCl_3$) δ 7.22 (ddd, *J* = 8.1, 7.5, 1.8 Hz, 1H), 7.14 – 7.09 (m, 1H), 7.04 – 6.97 (m, 2H), 3.87 (s, 3H).

N-(4-Chloro-5*H*-1,2,3-dithiazol-5-ylidene)-3,4-dimethylaniline (**2q**). Brown solid (0.900 g, 88%), m.p. 75-76 °C. IR (neat) ν_{max}: 2967, 2115, 1843, 1772, 1612, 1440, 1166, 1137, 1109, 847, 787, 754, 738, 702, 587, 443 cm^{-1}. ^{1}H NMR (300 MHz, $CDCl_3$) δ 7.21 (d, *J* = 7.9 Hz, 1H), 7.05 (d, *J* = 2.3 Hz, 1H), 7.01 (dd, *J* = 7.9, 2.3 Hz, 1H), 2.30 (s, 3H), 2.29 (s, 3H). ^{13}C NMR (75 MHz, $CDCl_3$) δ 157.25, 148.68, 148.33, 138.33, 135.55, 130.87, 121.44, 116.62, 77.58, 77.16, 76.74, 20.08, 19.67. HRMS (EI^+) m/z, calcd for $C_{10}H_{10}N_2S_2{}^{35}Cl$ $[M]^+$: 256.9974, found: 256.9975.

N-(4-Chloro-5*H*-1,2,3-dithiazol-5-ylidene)-3,4-dimethoxyaniline (**2**r). Red powder (0.707 g, 61%), m.p. 115-116°C. IR (neat) ν_{max}: 1574, 1508, 1463, 1416, 1329, 1264, 1239, 1122, 1018, 868, 742 cm^{-1}. ^{1}H NMR (300 MHz, $CDCl_3$) δ 6.94 – 6.91 (m, 2H), 6.88 – 6.85 (m, 1H), 3.91 (s, 3H), 3.90 (s, 3H). ^{13}C NMR (75 MHz, $CDCl_3$) δ 156.21, 149.85, 148.41, 147.93, 143.80, 111.36, 110.88, 105.84, 56.19, 56.12. HRMS (EI^+) m/z, calcd for $C_{10}H_{10}N_2O_2S_2{}^{35}Cl$ $[M]^+$: 288.9872, found: 288.9879.

4-Bromo-*N*-(4-chloro-5*H*-1,2,3-dithiazol-5-ylidene)-3-methylaniline (**2s**). Yellow powder (1.08 g, 84%) m.p. 86-87 °C .IR (neat) ν_{max}: 2913, 2361, 2117, 1876, 1761, 1597, 1468, 1167, 1026, 880, 852, 809, 793, 527 cm^{-1}. ^{1}H NMR (300 MHz, $CDCl_3$) δ 7.60 (d, *J* = 8.4 Hz, 1H), 7.10 (dd, *J* = 2.6, 0.8 Hz, 1H), 6.93 (dd, *J* = 8.4, 2.6 Hz, 1H), 2.43 (s, 3H).^{13}C NMR (75 MHz, $CDCl_3$) δ 158.93, 150.30, 148.13, 139.85, 133.76, 122.28, 122.24, 118.22, 77.58, 77.16, 76.74, 23.24. HRMS (EI^+) m/z, calcd for $C_9H_7N_2S_2{}^{35}Cl^{79}Br$ $[M]^+$: 320.8912, found: 320.8928.

N-(4-Chloro-5*H*-1,2,3-dithiazol-5-ylidene)benzo[*d*][1,3]dioxol-5-amine (**2t**) [68]. Red powder (0.287 g, 26%), m.p. 109-110 °C. IR (neat) ν_{max}: 1553, 1475, 1343, 1251, 1031, 930, 859, 766, 649 cm^{-1}. ^{1}H NMR (300 MHz, $CDCl_3$) δ 6.88 (dd, *J* = 8.2, 0.5 Hz, 1H), 6.84 – 6.77 (m, 2H), 6.03 (s, 2H).

N-(4-Chloro-5*H*-1,2,3-dithiazol-5-ylidene)-2,3-dihydrobenzo[*b*][1,4]dioxin-6-amine (**2u**) [69]. Yellow powder (0.750 g, 65%), m.p. 129-130 °C. IR (neat) ν_{max}: 1606, 1565, 1500, 1312, 1287, 1244, 1210, 1165, 1063, 1039, 915, 854, 824, 584 cm^{-1}. ^1H NMR (300 MHz, $CDCl_3$) δ 6.94 (d, J = 8.5 Hz, 1H), 6.87 (d, J = 2.5 Hz, 2H), 6.82 (dd, J = 8.5, 2.5 Hz, 1H), 4.29 (s, 4H).

N-(4-Chloro-5*H*-1,2,3-dithiazol-5-ylidene)-2,3-dimethylaniline (**2v**). Orange solid (0.661 g, 64%), m.p. 97-98°C. IR (neat) ν_{max}: 2908, 1569, 1463, 1146, 854, 787, 771, 707 cm^{-1}. ^1H NMR (300 MHz, $CDCl_3$) δ 7.17 (t, *J* = 7.8 Hz, 1H), 7.06 (d, *J* = 7.8 Hz, 1H), 6.94 (dd, *J* = 7.8, 1.3 Hz, 1H), 2.33 (s, 3H), 2.18 (s, 3H). ^{13}C NMR (75 MHz, $CDCl_3$) δ 158.36, 150.52, 147.94, 138.74, 128.67, 128.12, 126.76, 113.55, 20.43, 13.96. HRMS (EI$^+$) m/z, calcd for $C_{10}H_{10}N_2S_2{}^{35}Cl$ [M]$^+$: 256.9974, found: 256.9984.

N-(4-Chloro-5*H*-1,2,3-dithiazol-5-ylidene)-2,3-dichloroaniline (**2w**). Orange solid (1.03 g, 87%), m.p. 91-92°C. IR (neat) ν_{max}: 1600, 1552, 1507, 1445, 1416, 1191, 1136, 1055, 913, 852, 786, 774, 708, 669 cm^{-1}. ^1H NMR (300 MHz, $CDCl_3$) δ 7.34 (dd, *J* = 7.9, 1.7 Hz, 1H), 7.27 (t, *J* = 7.9 Hz, 1H), 7.02 (dd, *J* = 7.9, 1.7 Hz, 1H). ^{13}C NMR (75 MHz, $CDCl_3$) δ 162.21, 150.76, 147.49, 134.65, 128.57, 127.60, 124.19, 116.78. HRMS (EI$^+$) m/z, calcd for $C_8H_4N_2S_2{}^{35}Cl_3$ [M]$^+$: 296.8881, found: 296.8893.

3-Chloro-*N*-(4-chloro-5*H*-1,2,3-dithiazol-5-ylidene)-2-methyl-aniline (**2x**). Orange solid (0.859 g, 77%), m.p. 120-121°C ^1H NMR (300 MHz, $CDCl_3$) δ 7.26 (dd, *J* = 8.0, 1.7 Hz, 2H), 7.24 – 7.17 (m, 1H), 7.02 – 6.96 (m, 1H), 2.30 (s, 3H). ^{13}C NMR (75 MHz, $CDCl_3$) δ 159.79, 151.78, 147.77, 136.16, 128.48, 127.92, 127.10, 114.63, 14.86. IR (neat) ν_{max}: 2161, 1597, 1553, 1441, 1377, 1160, 1130, 1015, 852, 783, 772, 704, 679 cm^{-1} HRMS (EI$^+$) m/z, calcd for $C_9H_7N_2S_2{}^{35}Cl_2$ [M]$^+$: 276.9428, found: 276.9433.

N-(4-Chloro-5*H*-1,2,3-dithiazol-5-ylidene)-2,4-difluoroaniline (**2y**) [47]. Yellow solid (0.990 g, 94%), m.p. 94-95°C. IR (neat) ν_{max}: 1608, 1589, 1494, 1475, 1441, 1279, 1151, 1104, 966, 861, 831, 813, 774, 598 cm^{-1}. ^1H NMR (300 MHz, $CDCl_3$) δ 7.20 (m, 1H), 7.01 – 6.91 (m, 2H).

N-(4-Chloro-5*H*-1,2,3-dithiazol-5-ylidene)-2,4-dimethoxyaniline (**2z**) [66]. Dark red oil (0.867 g, 75%). IR (neat) ν_{max}: 3001, 2936, 2384, 1600, 1578, 1495, 1454, 1306, 1282, 1205, 1157, 1119, 1025, 853, 823, 764 cm^{-1}. ^1H NMR (300 MHz, $CDCl_3$) δ 7.15 (dd, J = 8.3, 0.6 Hz, 1H), 6.57 – 6.51 (m, 2H), 3.86 (s, 3H), 3.84 (s, 3H).

N-(4-Chloro-5*H*-1,2,3-dithiazol-5-ylidene)-2-fluoro-4-methoxyaniline (**2aa**). Yellow powder (0.960 g, 92%), m.p. 112-113 °C. IR (neat) ν_{max}: 3062, 3009, 2982, 2948, 2841, 1616, 1576, 1486, 1435, 1300, 1164, 1112, 1034, 854, 783, 768, 587 cm^{-1}. ^1H NMR (300 MHz, $CDCl_3$) δ 7.25 – 7.17 (m, 1H), 6.81 – 6.76 (m, 1H), 6.75 (q, J = 1.6 Hz, 1H), 3.83 (s, 3H). ^{13}C NMR (75 MHz, $CDCl_3$) δ 159.28 (d, *J* = 10.1 Hz), 159.05, 154.01 (d, *J* = 170.3 Hz), 148.27, 131.65 (d, *J* = 11.5 Hz), 120.31 (d, *J* = 2.7 Hz), 110.20 (d, *J* = 3.2 Hz), 103.21 (d, *J* = 23.2 Hz), 55.92. ^{19}F NMR (282 MHz, $CDCl_3$) δ -117.86. HRMS (EI$^+$) m/z, calcd for $C_9H_7N_2OFS_2{}^{35}Cl$ [M]$^+$: 276.9672, found: 276.9683.

N-(4-Chloro-5*H*-1,2,3-dithiazol-5-ylidene)-3,5-dimethylaniline (**2ab**). Yellow solid (0.931 g, 91%), m.p. 77-78 °C. IR (neat) ν_{max}: 1577, 1512, 1378, 1257, 1158, 860, 778, 690 cm^{-1}. ^1H NMR (300 MHz, $CDCl_3$) δ 6.89 (s, 1H), 6.84 (s, 2H), 2.35 (s, 6H). ^{13}C NMR (75 MHz, $CDCl_3$) δ 158.12, 151.31, 148.15, 139.80 (2C), 128.44, 117.05 (2C), 21.53 (2C). HRMS (EI$^+$) m/z, calcd for $C_{10}H_{10}N_2S_2{}^{35}Cl$ [M]$^+$: 256.9974, found: 256.9976.

N-(4-Chloro-5*H*-1,2,3-dithiazol-5-ylidene)-3,5-dimethoxyaniline (**2ac**). Brown oil (0.515 g, 45%). IR (neat) ν_{max}: 3088, 3001, 2937, 2835, 1575, 1454, 1422, 1203, 1150, 1053, 861, 689 cm^{-1}. ^1H NMR (300 MHz, $CDCl_3$) δ 6.43 – 6.28 (m, 3H), 3.81 (s, 6H). ^{13}C NMR (75 MHz, $CDCl_3$) δ 179.25, 174.88, 162.07, 158.43, 153.55, 147.91, 98.97, 97.43, 55.68 (2C). HRMS (EI$^+$) m/z, calcd for $C_{10}H_{10}N_2O_2S_2{}^{35}Cl$ [M]$^+$: 288.9872, found: 288.9885.

3-Bromo-*N*-(4-chloro-5*H*-1,2,3-dithiazol-5-ylidene)-5-methylaniline (**2ad**). Brown amorphous solid (0.812 g, 63%). IR (neat) ν_{max}: 1685, 1656, 1610, 1586, 1557, 1497, 1434, 1113, 830, 775, 670 cm^{-1}. ^1H NMR

(300 MHz, $CDCl_3$) δ 7.21 (s, 1H), 7.17 (d, J = 2.0 Hz, 1H), 6.93 (s, 1H), 2.36 (s, 3H). ^{13}C NMR (75 MHz, $CDCl_3$) δ 159.82, 152.50, 148.02, 141.99, 130.15, 123.30, 119.36, 119.03, 21.41. HRMS (EI^+) m/z, calcd for $C_9H_7N_2S_2{}^{35}Cl^{79}Br$ $[M]^+$: 320.8923, found: 320.8935.

3-Bromo-*N*-(4-chloro-5*H*-1,2,3-dithiazol-5-ylidene)-5-methoxyaniline (**2ae**). Yellow powder (1.28 g, 95%), m.p. 81-82°C. IR (neat) ν_{max}: 1582, 1567, 1421, 1272, 1165, 1138, 1046, 969, 869, 827, 775, 684 cm^{-1}. ^1H NMR (300 MHz, $CDCl_3$) δ 6.94 – 6.91 (m, 2H), 6.88 – 6.85 (m, 1H), 3.91 (s, 3H), 3.90 (s, 3H). ^{13}C NMR (75 MHz, $CDCl_3$) δ 161.65, 160.32, 153.57, 147.88, 123.96, 115.53, 114.48, 104.40, 55.87. HRMS (EI^+) m/z, calcd for $C_9H_7N_2OS_2{}^{35}Cl^{79}Br$ $[M]^+$: 336.8872, found: 336.8881.

N-(4-Chloro-5*H*-1,2,3-dithiazol-5-ylidene)-2,5-dimethylaniline (**2af**) [50]. Brown solid (0.710 g, 69%), m.p. 69-70 °C. IR (neat) ν_{max}: 2917, 1888, 1594, 1497, 1377, 1250, 1159, 1144, 865, 845, 808, 657, 611, 563, 250, 434 cm^{-1}. ^1H NMR (300 MHz, $CDCl_3$) δ 7.18 (d, J = 7.9 Hz, 1H), 6.96 (dd, J = 7.9, 1.7 Hz, 1H), 6.88 (d, J = 1.7 Hz, 1H), 2.34 (s, 3H), 2.21 (s, 3H).

N-(4-Chloro-5*H*-1,2,3-dithiazol-5-ylidene)-5-isopropyl-2-methylaniline (**2ag**) [65]. Brown oil (0.870 g, 76%). IR (neat) ν_{max}: 2957, 2921, 2867, 1890, 1594, 1564, 1494, 1145, 882, 851, 819, 757, 644, 515 cm^{-1}. ^1H NMR (300 MHz, $CDCl_3$) δ 7.21 (d, *J* = 7.7 Hz, 1H), 7.02 (dd, *J* = 7.7, 1.8 Hz, 1H), 6.97 (d, *J* = 1.8 Hz, 1H), 2.90 (hept, *J* = 6.9 Hz, 1H), 2.22 (s, 3H), 1.24 (d, *J* = 6.9 Hz, 6H). ^{13}C NMR (75 MHz, $CDCl_3$) δ 158.21, 150.46, 148.37, 131.25, 127.40, 124.78, 113.84, 33.96, 24.13, 17.29. HRMS (EI^+) m/z, calcd for $C_{12}H_{14}N2S_2{}^{35}Cl$ $[M]^+$: 285.0287, found: 285.0281.

2.2 General procedure for the synthesis of *N*-arylcyanothioformamides (arylcarbamothioyl cyanides) (**3**).

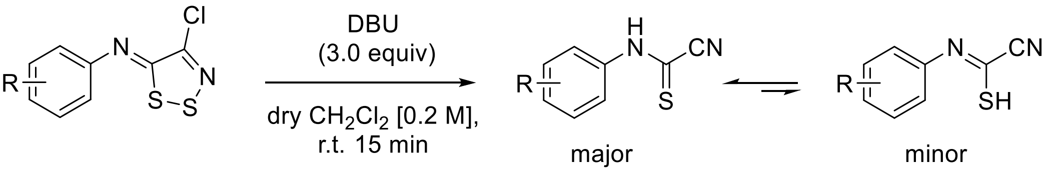

2 **3**

To a stirred solution of *N*-(4-chloro-5*H*-1,2,3-dithiazol-5-ylidene)-aniline **2** (2.0 mmol) in dry CH_2Cl_2 (10 mL) was added DBU (0.896 mL, 6.0 mmol). The reaction mixture was stirred under argon at room temperature for 15 min after which $NaHSO_4$ 1M (10 mL) was added. The resulting emulsion was extracted with CH_2Cl_2. The organic phase was washed with brine, dried over $MgSO_4$ and concentrated under reduced pressure. The crude product was purified on silica gel with PE/CH_2Cl_2 (50:50 to 0:100, v/v) as eluent to afford the desired product.

N-(4-Tolyl)cyanothioformamide (**3a**) [57]. Yellow powder (0.166 g, 46%, ratio major/minor 85:15), m.p. 133-134 °C. IR (neat) ν_{max}: 3262, 3121, 2225 (CN), 1888, 1608, 1504, 1397, 1081, 806, 729, 501 cm^{-1}. ^1H NMR (300 MHz, DMSO-d6) δ 13.40 (s, 1H, NH), 7.84 – 7.73 (m, 1.7H, major), 7.37 (d, J = 8.5 Hz, 0.3H), 7.29 (dd, *J* = 9.4, 2.8 Hz, 2H), 2.33 (s, 0.6H, minor), 2.32 (s, 2.4H, major).

N-Phenylcyanothioformamide (**3b**) [57]. Dark orange powder (0.133 g, 41%, ratio major/minor 90:10), m.p. 89-90 °C. IR (neat) ν_{max}: 3264, 3133, 3087, 2229 (CN), 1791, 1733, 1550, 1486, 1095, 757, 681 cm^{-1}. ^1H NMR (300 MHz, DMSO-d6) δ 13.47 (s, 1H, NH), 7.92 – 7.85 (m, 1.8H, major), 7.53 – 7.45 (m, 2.6H, major and minor), 7.39 – 7.33 (m, 0.9H, minor).

N-(4-Fluorophenyl)cyanothioformamide (**3c**) [57]. Orange powder (0.156 g, 43%, ratio major/minor 85:15), m.p. 108-109 °C. IR (neat) ν_{max}: 3258, 3080, 2240 (CN), 1502, 1418, 1224, 830, 514 cm^{-1}. ^1H NMR (300 MHz, DMSO-d6) δ 13.48 (s, 1H), 7.95 – 7.88 (m, 1.7H, major), 7.59 – 7.53 (m, 0.3H, minor), 7.39 – 7.29 (m, 2H) .^{19}F NMR (282 MHz, DMSO-d6) δ -112.38 (s, major), -112.85 (s, minor).

N-(4-Chlorophenyl)cyanothioformamide (**3d**) [57]. Orange powder (0.179 g, 45%, ratio major/minor 90:10), m.p. 125-126 °C. IR (neat) ν_{max}: 3271, 3059, 2229 (CN), 1602, 1539, 1487, 1412, 1374, 1284, 1103, 109, 1008, 822, 744, 503 cm^{-1}. ^{1}H NMR (300 MHz, DMSO-d6) δ 13.54 (s, 1H), 7.96 – 7.88 (m, 1.8H), 7.61 – 7.51 (m, 2.2H).

N-(4-Bromophenyl)cyanothioformamide (**3e**). Orange powder (0.225 g, 47%, ratio major/minor 85:15), m.p. 135-136 °C. IR (neat) ν_{max}: 3271, 3121, 2229 (CN), 1543, 1485, 1391, 1097, 1485, 1391, 1097, 1075, 1010, 825, 807, 730, 502 cm^{-1}. ^{1}H NMR (300 MHz, DMSO-d6) δ 13.53 (s, 1H), 7.90 – 7.82 (m, 1.7H, minor), 7.73 – 7.66 (m, 2H), 7.51 – 7.43 (m, 0.3H, minor). ^{13}C NMR (75 MHz, DMSO-d6) δ 161.59, 136.92, 132.45, 132.04, 125.30, 124.60, 119.91, 113.74. HRMS (EI$^-$) m/z, calcd for $C_8H_4N_2S^{79}Br$ [M]$^-$: 240.9258, found: 240.9261.

N-(4-Methoxyphenyl)cyanothioformamide (**3f**) [57]. Orange powder (0.125 g, 33%, ratio major/minor 85:15), m.p. 122-123 °C. IR (neat) ν_{max}: 3248, 3064, 2238 (CN), 1507, 1390, 1248, 1021, 824, 724, 578, 514 cm^{-1}. ^{1}H NMR (300 MHz, DMSO-d6) δ 13.36 (s, 1H), 7.91 - 7.83 (m, 1.7H, major), 7.43-7.40 (m, 0.3H, minor), 7.08 – 7.00 (m, 2H), 3.79 (s, 0.5 H, minor), 3.78 (s, 2.5H, major).

N-[(4-Trifluoromethyl)phenyl]cyanothioformamide (**3g**) [57]. Brown powder (0.259 g, 56%, ratio major/minor 85:15), m.p. 108-109 °C. IR (neat) ν_{max}: 3269, 3075, 2234 (CN), 1611, 1548, 1513, 1373, 1322, 1172, 1116, 1132, 1110, 1012, 838, 730, 615, 589, 508, 459 cm^{-1}. ^{1}H NMR (300 MHz, DMSO-d6) δ 13.67 (s, 1H), 8.10 (d, *J* = 8.4 Hz, 2H, major), 7.87 (d, *J* = 8.7 Hz, 2.3H, major and minor), 7.74 (d, *J* = 8.4 Hz, 0.3H, minor). ^{19}F NMR (282 MHz, DMSO-d6) δ -60.99 (minor), -61.00 (major).

N-(4-Cyanophenyl)cyanothioformamide (**3h**). Orange powder (0.261 g, 71%, ratio major/minor 85:15), m.p. 181-182 °C. IR (neat) ν_{max}: 3249, 3013, 2236 (CN), 1603, 1544, 1498, 1418, 1376, 1108, 835, 546 cm^{-1}. ^{1}H NMR (300 MHz, DMSO-d6) δ 13.87 (s, 1H), 8.13 – 8.07 (m, 1.9H, minor), 8.01 – 7.92 (m, 2.2H, major), 7.78 – 7.50 (m, 0.3H, minor). ^{13}C NMR (75 MHz, DMSO-d6) δ 162.58, 141.53, 133.77 (minor), 133.43 (major), 123.40 (minor), 122.94 (major), 118.34, 113.67, 109.53. HRMS (EI$^-$) m/z, calcd for $C_9H_4N_3S$ [M]$^-$: 186.0126, found: 186.0120.

N-(4-Nitrophenyl)cyanothioformamide (**3i**) [57]. Orange solid (0.134 g, 32%, ratio major/minor 80:20), m.p. 130-131 °C. IR (neat) ν_{max}: 3269,3100, 2238 (CN), 1567, 1514, 1384, 1342, 1109, 849, 749, 681, 495 cm^{-1}. ^{1}H NMR (300 MHz, DMSO-d6) δ 13.81 (s, 0.8H, major), 8.39 – 8.29 (m, 2H, major and minor), 8.17 (d, *J* = 8.7 Hz, 1.6H, major), 7.77 (m, 0.4H, minor).

Ethyl 4-[(cyanocarbomothioyl)amino]benzoate (**3j**) [57]. Red powder (0.386 g, 82%, ratio major/minor 90:10), m.p. 177-178 °C. IR (neat) ν_{max}: 3264, 3118, 3070, 2230 (CN), 1680, 1604, 1551, 1389, 1366, 1309, 1289, 1177, 1015, 852, 771, 690 cm^{-1}. ^{1}H NMR (300 MHz, DMSO-d6) δ 13.69 (s, 1H), 8.05 (s, 3.8H, major), 7.65 (d, *J* = 8.7 Hz, 0.2H, minor), 4.33 (qd, *J* = 7.1, 2.4 Hz, 2H, major and minor), 1.32 (t, *J* = 7.1 Hz, 3H, major and minor).

N-(3-Methoxyphenyl)cyanothioformamide (**3k**) [57]. Orange powder (0.260 g, 68%, ratio major/minor 90:10), m.p. 91-92 °C. IR (neat) ν_{max}: 3267, 3221, 3152, 3094, 2228 (CN), 1617, 1558, 1492, 1451, 1396, 1265, 1172, 1159, 846, 779, 678 cm^{-1}. ^{1}H NMR (300 MHz, DMSO-d6) δ 13.44 (s, 1H), 7.61 (t, *J* = 2.2 Hz, 0.8H, major), 7.47 – 7.35 (m, 1.8H, major and minor), 7.11 – 6.91 (m, 1.4H, major and minor), 3.79 (s, 0.6H, minor), 3.76 (s, 2.4H, major).

N-(3-Nitrophenyl)cyanothioformamide (**3l**) [57]. Orange solid (0.334 g, 81%, ratio major/minor 90:10), m.p. 137-138°C. IR (neat) ν_{max}: 3275, 3095, 2237 (CN), 1524, 1382, 1347, 811, 739, 668, 398 cm^{-1}. ^{1}H NMR (300 MHz, DMSO-d6) δ 13.83 (s, 0.8H, major), 8.91 (t, *J* = 2.2 Hz, 0.8H, major), 8.39 (s, 0.2H, minor), 8.25 – 8.17 (m, 1.6H, major), 8.00 (d, *J* = 8.0 Hz, 0.2H, minor), 7.79 (t, *J* = 8.2 Hz, 1H, major and minor).

Ethyl 3-[(cyanocarbonothioyl)amino]benzoate (**3m**). Orange powder (0.352 g, 75%, ratio major/minor 85:15), m.p. 137-138 °C. IR (neat) ν_{max}: 3219, 2988, 2934, 2901, 2234 (CN), 1687, 1520, 1365, 1285, 1211, 1125, 1014, 731, 674 cm^{-1}. ^{1}H NMR (300 MHz, DMSO-d6) δ 13.63 (s, 0.85H, major), 8.52 (t, *J* = 1.9 Hz, 0.85H, major), 8.13 (ddd, *J* = 8.0, 2.3, 1.1 Hz, 0.85H, major), 8.04 (t, *J* = 1.9 Hz, 0.15H, minor), 7.98 (dt, *J* = 7.8, 1.4 Hz, 0.15H, minor), 7.92 (dt, *J* = 7.8, 1.4 Hz, 0.85H, major), 7.83 (ddd, *J* = 8.0, 2.3, 1.1 Hz, 0.15H, minor), 7.64 (t, *J* = 8.0 Hz, 1H, major and minor), 4.34 (qd, *J* = 7.1, 3.2 Hz, 2H, major and minor), 1.32 (t, *J* = 7.1 Hz, 3H, major and minor). ^{13}C NMR (75 MHz, DMSO-d6) δ 164.85, 162.12, 138.35 (minor), 137.95 (major), 130.69, 129.71, 128.13, 127.83 (minor), 127.17, 123.19, 113.70, 61.21 (minor), 61.15 (major), 14.10. HRMS (EI$^-$) m/z, calcd for $C_{11}H_9N_2O_2S$ [M]$^-$: 233.0385, found: 233.0388.

N-(2-Chlorophenyl)cyanothioformamide (**3n**). Red powder (0.327 g, 83%), m.p. 109-110 °C. IR (neat) ν_{max}: 3230, 2220 (CN), 1585, 1525, 1379, 1105, 1032, 734 cm^{-1}. ^{1}H NMR (300 MHz, DMSO-d6) δ 13.46 (s, 1H, NH), 7.74 – 7.61 (m, 1H, major and minor), 7.56 – 7.41 (m, 3H, major and minor). ^{13}C NMR (75 MHz, DMSO-d6) δ 167.71 (minor), 165.02 (major), 135.58 (minor), 133.88 (major), 131.08 (minor), 130.40 (minor), 130.31 (major), 130.25 (major), 129.54 (minor), 129.50 (major), 128.65 (minor), 128.57 (major and minor), 128.27 (major), 113.62 (major), 112.15 (minor). HRMS (EI$^-$) m/z, calcd for $C_8H_4N_2S^{35}Cl$ [M]$^+$: 194.9784, found: 194.9777.

N-(2-Bromophenyl)cyanothioformamide (**3o**) [57]. Brown powder (0.384 g, 80%, ratio major/minor 80:20), m.p. 102-103 °C. IR (neat) ν_{max}: 3229, 2237 (CN), 1579, 1520, 1439, 1375, 1104, 1025, 733 cm^{-1}. ^{1}H NMR (300 MHz, DMSO-d6) δ 13.45 (s, 1H, NH), 7.84 (dd, *J* = 8.0, 1.2 Hz, 0.2H, minor), 7.79 (d, *J* = 8.0 Hz, 0.8H, major), 7.70 (dd, *J* = 8.0, 1.2 Hz, 0.2H, minor), 7.55 (td, *J* = 8.0, 1.2 Hz, 0.2H, minor), 7.52-7.48 (m, 1.6H, major), 7.44 (td, *J* = 8.0, 1.6 Hz, 0.2H, minor), 7.40-7.33 (m, 0.8H, major).

N-(2-Methoxyphenyl)cyanothioformamide (**3p**). Orange powder (0.303 g, 79%, ratio major/minor 75:25), m.p. 110-111 °C. IR (neat) ν_{max}: 3244, 2240 (CN), 1604, 1532, 1396, 1259, 1114, 1022, 739 cm^{-1}. ^{1}H NMR (300 MHz, DMSO-d6) δ 13.05 (s, 1H, NH), 7.55 (dd, *J* = 8.0, 1.7 Hz, 0.75H, major), 7.48 – 7.34 (m, 1.25H, major and minor), 7.20 (ddd, *J* = 11.8, 8.0, 1.3 Hz, 1H, major and minor), 7.03 (qd, *J* = 8.0, 1.3 Hz, 1H, major and minor), 3.86 (s, 1H, minor), 3.83 (s, 2H, major). ^{13}C NMR (75 MHz, DMSO-d6) δ 167.51 (minor), 163.65 (major), 153.48 (minor), 152.76 (major), 130.53 (minor), 129.77 (major), 126.78 (minor), 126.60 (minor), 126.45 (major), 124.84 (minor), 120.79 (minor), 120.34 (major), 113.78 (major), 112.76 (major), 112.63 (minor), 112.53 (major), 55.83 (minor), 55.78 (major). HRMS (EI$^+$) m/z, calcd for $C_9H_7N_2OS^{35}Cl$ [M]$^+$: 191.0279, found: 191.0272.

N-(3,4-Dimethylphenyl)cyanothioformamide (**3q**). Orange powder (0.198 g, 52%, ratio major/minor 85:15), m.p. 131-132 °C. IR (neat) ν_{max}: 3262, 3124, 2996, 2223 (CN), 1600, 1498, 1415, 1383, 1090, 858, 739, 605 cm^{-1}. ^{1}H NMR (300 MHz, DMSO-d6) δ 13.36 (s, 1H, NH), 7.68 – 7.60 (m, 1.6H, major), 7.28 – 7.17 (m, 1.4H, minor), 2.23 (d, J = 2.2 Hz, 6H, major and minor). ^{13}C NMR (75 MHz, DMSO-d6) δ 164.35 (minor), 160.60 (major), 137.74 (minor), 137.10 (major), 136.93 (minor), 135.46, 130.29 (minor), 129.83 (major), 124.09 (minor), 123.33 (major), 120.73 (minor), 120.01 (major), 113.81, 19.50 (major), 19.33 (minor), 19.15 (major), 19.00 (minor). HRMS (EI$^-$) m/z, calcd for $C_{10}H_9N_2S$ [M]$^-$: 189.0486, found: 189.0483.

N-(3,4-Dimethoxyphenyl)cyanothioformamide (**3r**) [57]. Orange powder (0.314 g, 71%, ratio major/minor 90:10), m.p. 109-110 °C. ^{1}H NMR (300 MHz, DMSO-d6) δ 13.37 (s, 1H, NH), 7.65 (d, *J* = 2.5 Hz, 0.8H, major), 7.54 (dd, *J* = 8.8, 2.5 Hz, 0.8H, major), 7.15 – 7.11 (m, 0.2H, minor), 7.05 (d, *J* = 8.6 Hz, 1.2H, major and minor), 3.79 (d, *J* = 1.3 Hz, 3H, major), 3.78 (s, 0.6H, minor), 3.74 (s, 2.4H, major). IR (neat) νmax: 3264, 3086, 2228 (CN), 1509, 1407, 1269, 1163, 1143, 1019, 799, 610 cm^{-1}.

N-(4-Bromo-3-methylphenyl)cyanothioformamide (**3s**). Orange powder (0.509 g, 63%, ratio major/minor 90:10). m.p. 142-143 °C. IR (neat) ν_{max}: 3262, 3072, 2227 (CN), 1610, 1579, 1472, 1388, 1096, 1025, 810, 746, 431 cm^{-1}. ^{1}H NMR (300 MHz, DMSO-d6) δ 13.51 (s, 1H, NH), 7.86 (dd, J = 1.9, 1.0 Hz, 0.8 H, major), 7.77 – 7.63 (m, 2.2H, major and minor), 7.50 (d, J = 2.8 Hz, 0.2H, minor), 7.30 (dd, J = 8.5, 2.8 Hz, 0.2H, minor), 2.38 (2 s, 3H, major and minor). ^{13}C NMR (75 MHz, DMSO-d6) δ 164.98 (minor), 161.58 (major), 138.85 (minor), 138.29 (major), 137.45 (minor), 136.97 (major), 133.06 (minor), 132.68 (major), 125.62 (minor), 124.81 (major), 123.59 (minor), 122.71 (minor), 122.46 (major), 122.05 (major), 113.73 (major), 112.52 (minor), 22.59 (major), 22.44 (minor). HRMS (EI^-) m/z, calcd for $C_9H_6N_2S^{79}Br$ $[M]^-$: 252.9435, found: 252.9431.

N-(Benzo[*d*][1,3]dioxol-5-yl)cyanothioformamide (**3t**). Red powder (0.266 g, 55%, ratio major/minor 90:10), m.p. 128-129 °C. IR (neat) ν_{max}: 3280, 3098, 2917, 2218 (CN), 1498, 1486, 1394, 1363, 1256, 1040, 929, 840, 745, 732, 601, 422 cm^{-1}. ^{1}H NMR (300 MHz, DMSO-d6) δ 13.36 (s, 1H, NH), 7.58 (d, *J* = 2.1 Hz, 0.8H, major), 7.36 (dd, *J* = 8.8, 1.9 Hz, 0.8H, major), 7.13 (s, 0.2H, minor), 7.02 (d, *J* = 8.5 Hz, 1H, major and minor), 6.98 – 6.93 (m, 0.2H, minor), 6.12 (s, 0.4H, minor), 6.10 (s, 1.6H, major). ^{13}C NMR (75 MHz, DMSO-d6) δ 164.96 (minor), 160.14 (major), 147.79 (minor), 147.11 (major), 146.37, 132.18 (minor), 131.79 (major), 117.59 (minor), 116.71 (major), 113.82, 108.40 (minor), 108.25 (major), 104.95 (minor), 103.73 (major), 102.11 (minor), 101.95 (major). HRMS (EI^-) m/z, calcd for $C_9H_5N_2O_2S$ $[M]^-$: 205.0072, found: 206.0065.

N-(2,3-Dihydrobenzo[*b*][1,4]dioxin-6-yl)cyanothioformamide (**3u**). Orange powder (0.314 g, 71%, ratio major/minor 85:15), m.p. 119-120 °C. IR (neat) ν_{max}: 3259, 3136, 3088, 2229 (CN), 1604, 1503, 1401, 1295, 1063, 845, 604 cm^{-1}. ^{1}H NMR (300 MHz, DMSO-d6) δ 13.32 (s, 1H, NH), 7.64 (d, *J* = 2.6 Hz, 0.85H, major), 7.34 (dd, *J* = 8.8, 2.6 Hz, 0.85H, major), 7.03 (dd, *J* = 1.8, 1.1 Hz, 0.15H, minor), 6.99 – 6.90 (m, 1.15H, major and minor), 4.28 (s, 1H, minor), 4.27 (s, 3H, major). ^{13}C NMR (75 MHz, DMSO-d6) δ 164.07 (minor), 159.36 (major), 143.20 (minor), 143.18 (minor), 142.52 (major), 142.44 (major), 131.18 (minor), 131.02 (major), 117.28 (minor), 116.89 (major), 116.25 (minor), 115.64 (major), 113.57, 112.07 (minor), 110.97 (major), 63.93, 63.85. HRMS (EI^-) m/z, calcd for $C_{10}H_7N_2O_2S$ $[M]^-$: 219.0228, found: 219.0222.

N-(2,3-Dimethylphenyl)cyanothioformamide (**3v**). Red solid (0.300 g, 79%, ratio major/minor 75:25), m.p. 87-88 °C. IR (neat) νmax: 3252, 2949, 1600, 1504, 1469, 1458, 1380, 1121, 747 cm^{-1}. ^{1}H NMR (300 MHz, DMSO-d6) δ 13.19 (s, 1H, NH), 7.31 – 7.10 (m, 3H, major and minor), 2.30 (s, 0.75H, minor), 2.28 (s, 2.25H, major), 2.16 (s, 0.75H, minor), 2.06 (s, 2.25H, major). ^{13}C NMR (75 MHz, DMSO-d6) δ 166.76 (minor), 164.39 (major), 138.36 (minor), 138.11 (major), 137.23 (minor), 135.14 (major), 132.34 (major), 132.06 (minor), 130.62 (minor), 129.87 (major), 126.38 (minor), 126.14 (major), 124.17 (minor), 123.68 (major), 113.76 (minor), 19.87 (major), 19.82 (minor), 14.11 (minor), 13.96 (major). HRMS (EI^-) m/z, calcd for $C_{10}H_9N_2S$ $[M]^-$: 189.0486, found: 189.0478.

N-(2,3-Dichlorophenyl)cyanothioformamide (**3w**) [57]. Orange powder (0.361 g, 78%, ratio major/minor 65:35), m.p. 143-144°C. IR (neat) ν_{max}: 3221, 3025, 1530, 1453, 1371, 1188, 1114, 911, 786, 743, 668, 608 cm^{-1}. ^{1}H NMR (300 MHz, DMSO-d6) δ 13.58 (s, 1H, NH), 7.78 (ddd, *J* = 9.4, 8.1, 1.5 Hz, 0.35H, minor), 7.72 (dd, *J* = 7.7, 1.9 Hz, 1H, major), 7.58 – 7.46 (m, 2.35H, major and minor).

N-(3-Chloro-2-methylphenyl)cyanothioformamide (**3x**). Red solid (0.360 g, 85%, ratio major/minor 85:15), m.p. 98-99°C. IR (neat) ν_{max}: 3228, 2988, 2240 (CN), 1507, 1390, 1112, 1014, 743, 655 cm^{-1}. ^{1}H NMR (300 MHz, DMSO-d6) δ 13.35 (s, 1H, NH), 7.60 – 7.53 (m, 0.15H, minor), 7.53 – 7.48 (m, 0.85H, major), 7.41 – 7.36 (m, 0.3H, minor), 7.35 – 7.30 (m, 1.7H, major), 2.31 (s, 0.45H, minor), 2.20 (s, 2.55H, major). ^{13}C NMR (75 MHz, DMSO-d6) δ 167.05 (minor), 164.89 (major), 138.58 (minor), 136.69 (major), 134.43 (major), 132.19 (major), 131.77 (minor), 129.93 (minor), 129.26 (major), 128.02 (minor), 127.84 (major), 125.80 (minor), 125.48 (major), 113.65 (major), 112.23 (minor), 15.13 (minor), 14.96 (major). HRMS (EI^-) m/z, calcd for $C_9H_6N_2S^{35}Cl$ $[M]^-$: 208.9940, found: 208.9945.

N-(2,4-Difluorophenyl)cyanothioformamide (**3y**) [57]. Red orange powder (0.254 g, 64%, ratio major/minor 85:15), m.p. 101-102°C. IR (neat) ν_{max}: 3246, 1603, 1496, 1384, 1295, 1261, 1149, 1098, 965, 853, 813, 604 cm^{-1}. ^{1}H NMR (300 MHz, DMSO-d6) δ 13.37 (s, 1H, NH), 7.81 – 7.72 (m, 0.15H, minor), 7.67 (td, *J* = 8.8, 6.0 Hz, 0.85H, major), 7.58 (ddd, *J* = 10.6, 8.8, 2.8 Hz, 0.15H, minor), 7.50 (ddd, *J* = 10.7, 9.1, 2.8 Hz, 0.85H, major), 7.31 – 7.28 (m, 0.15H, minor), 7.23 (dddd, *J* = 9.1, 8.3, 2.8, 1.4 Hz, 0.85H, minor).

N-(2,4-Dimethoxyphenyl)cyanothioformamide (**3z**) [57]. Orange powder (0.335 g, 75%, ratio major/minor 70:30), m.p. 142-143 °C. IR (neat) ν_{max}: 3243, 3121, 3018, 2934, 2833, 2224 (CN), 1656, 1530, 1398, 1329, 1270, 1206, 1124, 1026, 831, 577 cm^{-1}. ^{1}H NMR (300 MHz, DMSO-d6) δ 12.87 (s, 1H, NH), 7.49 (d, *J* = 8.7 Hz, 0.7H, major), 7.32 (d, *J* = 8.7 Hz, 0.3H, minor), 6.75 (d, *J* = 2.6 Hz, 0.3H, minor), 6.71 (d, *J* = 2.6 Hz, 0.7H, major), 6.59 (m, 1H, major and minor), 3.85 (s, 1H, minor), 3.82 (s, 2H, major), 3.81 (s, 1H, minor), 3.80 (s, 2H, major).

N-(2-Fluoro-4-methoxyphenyl)cyanothioformamide (**3aa**). Yellow powder (0.315 g, 75%, ratio major/minor 90:10), m.p. 131-132 °C. IR (neat) ν_{max}: 3238, 3064, 2972, 2917, 2234 (CN), 1896, 1598, 1541, 1499, 1382, 1318, 1160, 1088, 1021, 836, 741, 605, 546, 453 cm^{-1}. ^{1}H NMR (300 MHz, DMSO-d6) δ 13.21 (s, 1H, NH), 7.53 (td, *J* = 8.9, 5.1 Hz, 1H, major and minor), 7.14 – 7.07 (m, 0.2H, minor), 7.04 (dd, *J* = 12.4, 2.6 Hz, 0.8H, major), 6.96 – 6.83 (m, 1H, major and minor), 3.82 (s, 0.6H, minor), 3.80 (s, 2.4H, major). ^{13}C NMR (75 MHz, DMSO-d6) δ 167.53, 164.39,160.69 (d, *J* = 10.5 Hz, minor), 160.34 (d, *J* = 10.5 Hz, major), 155.92 (d, *J* = 249.4 Hz), 128.25 (d, *J* = 2.8 Hz, minor), 127.78 (d, *J* = 2.8 Hz, major), 118.58 (d, *J* = 12.6 Hz, minor), 116.75 (d, *J* = 12.6 H, major), 113.72 (minor), 113.63 (major), 110.98 (d, *J* = 3.1 Hz, minor), 110.65 (d, *J* = 3.1 Hz, major) ,102.43 (d, *J* = 22.9 Hz, minor), 102.40 (d, *J* = 22.9 Hz, major), 56.08 (minor), 55.96 (major). ^{19}F NMR (282 MHz, DMSO-d6) δ -115.91 (major), -119.64 (minor). HRMS (EI$^-$) m/z, calcd for $C_9H_6N_2OFS$ [M]$^-$: 209.0185, found: 209.0178.

N-(3,5-Dimethylphenyl)cyanothioformamide (**3ab**). Orange powder (0.422 g, 77%, ratio major/minor 80:20), m.p. 102-103°C. IR (neat) ν_{max}: 3267, 3107, 2234 (CN), 1623, 1572, 1402, 1317, 1097, 843, 747, 677 cm^{-1}. ^{1}H NMR (300 MHz, DMSO-d6) δ 13.34 (s, 1H, NH), 7.57 – 7.41 (m, 1.6H, major), 7.08 (s, 0.6H, minor), 7.01 (tt, *J* = 1.5, 0.8 Hz, 0.8H, major), 2.30 (d, *J* = 0.7 Hz, 1.8H, minor), 2.29 (d, *J* = 0.7 Hz, 4.2H, major). ^{13}C NMR (75 MHz, DMSO-d6) δ 161.14, 138.97, 138.39, 137.54, 129.77 (minor), 129.30 (major), 120.89 (minor), 120.19 (major), 113.79, 20.90 (2C, major), 20.72 (2C, minor). HRMS (EI$^-$) m/z, calcd for $C_{10}H_9N_2S$ [M]$^-$: 189.0486, found: 189.0477.

N-(3,5-Dimethoxyphenyl)cyanothioformamide (**3ac**). Orange powder (0.288 g, 65%, ratio major/minor 90:10), m.p. 106-107 °C. IR (neat) ν_{max}: 3277, 3159, 3109, 2965, 2235 (CN), 1628, 1569, 1480, 1462, 1402, 1341, 1209, 1198, 1156, 1054, 668 cm^{-1} ^{1}H NMR (300 MHz, DMSO-d6) δ 13.40 (s, 1H, NH), 7.16 (d, *J* = 2.1 Hz, 1.6H, major), 6.68 (d, *J* = 2.1 Hz, 0.4H, minor), 6.55 (s, 0.2H, minor), 6.52 (t, *J* = 2.2 Hz, 0.8H, major), 3.77 (s, 1.2H, minor), 3.75 (s, 4.8H, major). ^{13}C NMR (75 MHz, DMSO-d6) δ 161.25, 160.81, 160.43, 139.54 (minor), 139.23 (major), 113.68, 101.63 (minor), 100.80 (major), 99.90 (minor), 99.56 (major), 55.59 (2C, minor), 55.45 (2C, major). HRMS (EI$^-$) m/z, calcd for $C_{10}H_9N_2O_2S$ [M]$^-$: 221.0385, found: 221.0382.

N-(3-Bromo-5-methylphenyl)cyanothioformamide (**3ad**). Orange powder (0.224 g, 44%, ratio major/minor 95:5), m.p. 127-128°C. IR (neat) ν_{max}: 3266, 3098, 2230 (CN), 1609, 1549, 1391, 1095, 844, 740, 669 cm^{-1}. ^{1}H NMR (300 MHz, DMSO-d6) δ 13.49 (s, 1H, NH), 7.97 (d, *J* = 2.0 Hz, 0.9H, major), 7.60 (s, 0.9H, major), 7.56 (s, 0.1H, minor), 7.48 (s, 0.1H, minor), 7.43 (dq, *J* = 1.6, 0.8 Hz, 0.9H, major), 7.33 (s, 0.1H, minor), 2.35 – 2.34 (m, 0.4H, minor), 2.33 (d, *J* = 0.7 Hz, 2.6H, major). ^{13}C NMR (75 MHz, DMSO-d6) δ 162.10, 141.26, 138.73, 130.99, 122.34, 122.29, 121.14, 113.68, 20.64. HRMS (EI$^-$) m/z, calcd for $C_9H_6N_2S^{81}Br$ [M]$^-$: 254.9415, found: 254.941.

N-(3-Bromo-5-methoxyphenyl)cyanothioformamide (**3ae**). Orange powder (0.463 g, 85%, ratio major/minor 85:15), m.p. 123-124 °C. IR (neat) ν_{max}: 3263, 3086,2230 (CN), 1606, 1591, 1554, 1459, 1395, 1328, 1278, 1166, 1050, 834, 740, 665 cm^{-1}. ^{1}H NMR (300 MHz, DMSO-d6) δ 13.50 (s, 1H, NH), 7.71 (t, *J* = 1.8 Hz, 0.85H, major), 7.51 (t, *J* = 2.1 Hz, 0.85H, major), 7.30 (t, *J* = 1.8 Hz, 0.15H, minor), 7.23 – 7.21 (m, 0.15H, minor), 7.21 – 7.14 (m, 1H, major and minor), 3.81 (s, 0.5H, minor), 3.79 (s, 2.5H, major). ^{13}C NMR (75 MHz, DMSO-d6) δ 162.11, 160.53 (minor), 160.25 (major), 139.65, 121.96, 117.36, 116.46 (minor) 115.97 (major), 113.63, 108.01, 56.03 (minor), 55.89 (major). HRMS (EI$^-$) m/z, calcd for $C_9H_6N_2OS^{81}Br$ [M]$^-$: 270.9364, found: 370.9362.

N-(2,5-Dimethylphenyl)cyanothioformamide (**3ag**). Yellow powder (0.285 g, 77%, ratio major/minor 85:15), m.p. 75-76 °C. IR (neat) ν_{max}: 3529, 3009, 2976, 2915, 2228 (CN), 1625, 1578, 1388, 1096, 802, 764, 672, 449 cm^{-1}. ^{1}H NMR (300 MHz, DMSO-d6) δ 13.14 (s, 1H, NH), 7.29 – 7.07 (m, 3H, major and minor), 2.30 (s, 0.7H, minor), 2.28 (s, 2.3H, major), 2.22 (s, 0.7H, minor), 2.13 (s, 2.3H, major). ^{13}C NMR (75 MHz, DMSO-d6) δ 166.69 (minor), 164.10 (major), 137.06 (minor), 136.10 (minor), 134.93 (major), 130.89 (major), 130.83 (major), 130.53 (minor), 130.17 (major), 129.92 (minor), 129.31 (minor), 126.69 (minor), 126.18 (major), 113.71 (major), 112.39 (minor), 30.68, 20.34 (major), 20.25 (minor), 16.94. HRMS (EI$^-$) m/z, calcd for $C_{10}H_9N_2S$ [M]$^-$: 189.0486, found: 189.0483.

N-(5-Isopropyl-2-methylphenyl)cyanothioformamide (**3ah**). Brown oil (0.340 g, 78%, ratio major/minor 75:25). IR (neat) νmax: 3436, 3023, 2954, 2225 (CN), 1635, 1526, 1365, 1102, 811, 759 cm^{-1}. ^{1}H NMR (300 MHz, DMSO-d6) δ 13.12 (s, 1H, NH), 7.34 (d, *J* = 1.6 Hz, 0.3H, minor), 7.29 – 7.16 (m, 2.7H, major), 2.96 – 2.80 (m, 1H, major and minor), 2.24 (s, 0.75H, minor), 2.15 (s, 2.25H, major), 1.18 (dd, *J* = 6.9, 6.2 Hz, 6H, major and minor). ^{13}C NMR (75 MHz, DMSO-d6) δ 166.64 (minor), 164.04 (major), 147.54 (minor), 147.08 (major), 137.09 (minor), 134.96 (major), 130.91 (major), 130.87, 130.30 (minor), 127.17 (minor), 126.61 (major), 124.27 (minor), 123.69 (major), 113.72 (major), 112.47 (minor), 32.86 (minor), 32.82 (major), 23.72 (2C, major), 23.62 (2C, minor), 16.99 (major), 16.96 (minor). HRMS (EI$^-$) m/z, calcd for $C_{12}H_{13}N_2S$ [M]$^-$: 217.0799, found: 217.0791

3. ^{1}H NMR (300 MHz, $CDCl_3$), ^{19}F NMR (282 MHz, $CDCl_3$) and ^{13}C NMR (75 MHz, $CDCl_3$) of 2-cyanobenzothiazoles **4a-z** and **4aa-4ag**

4a

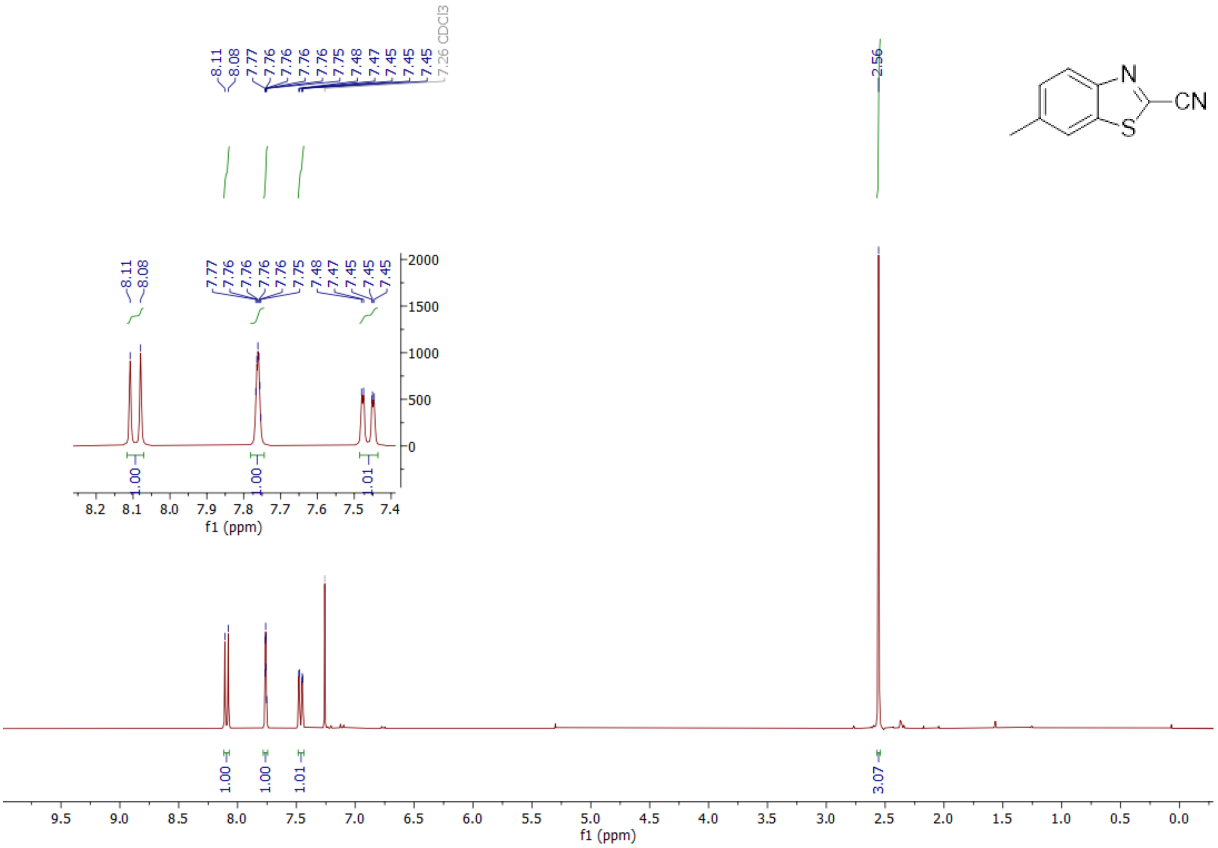

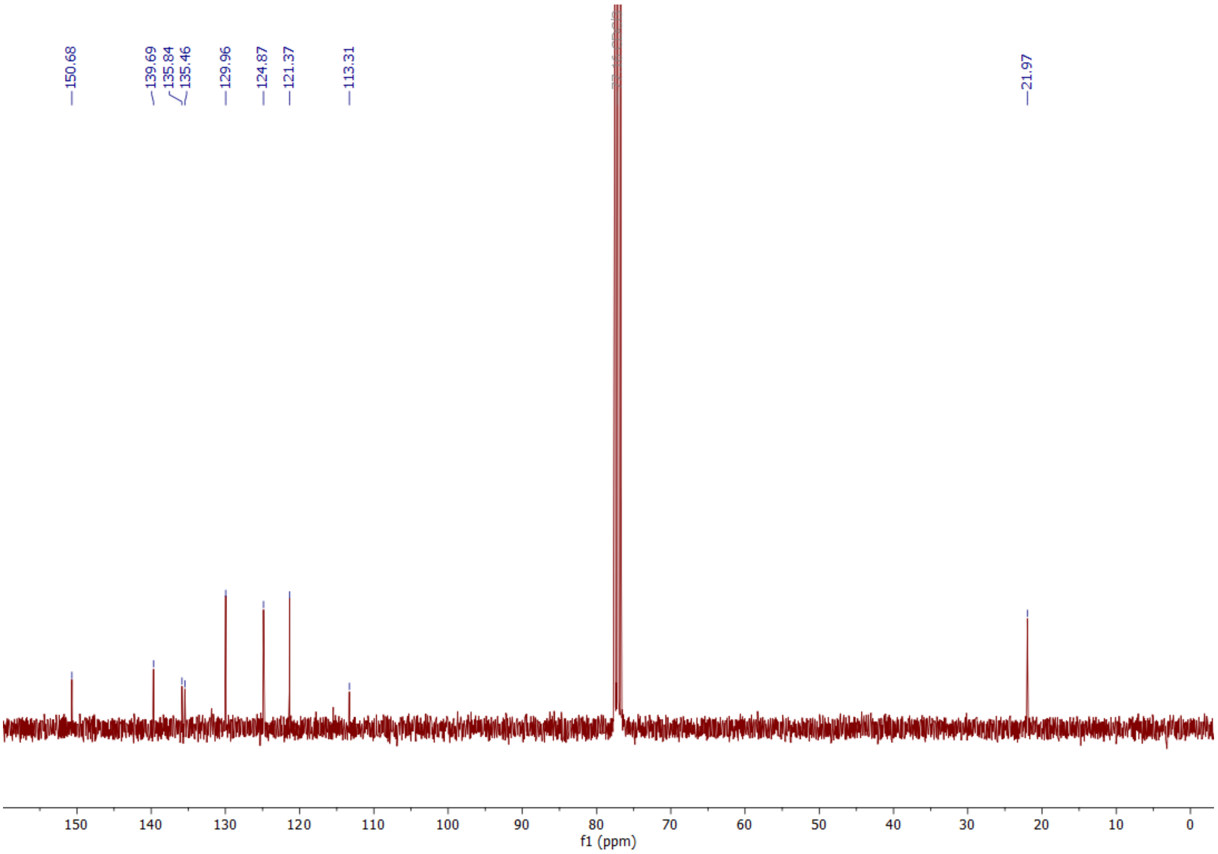

4b

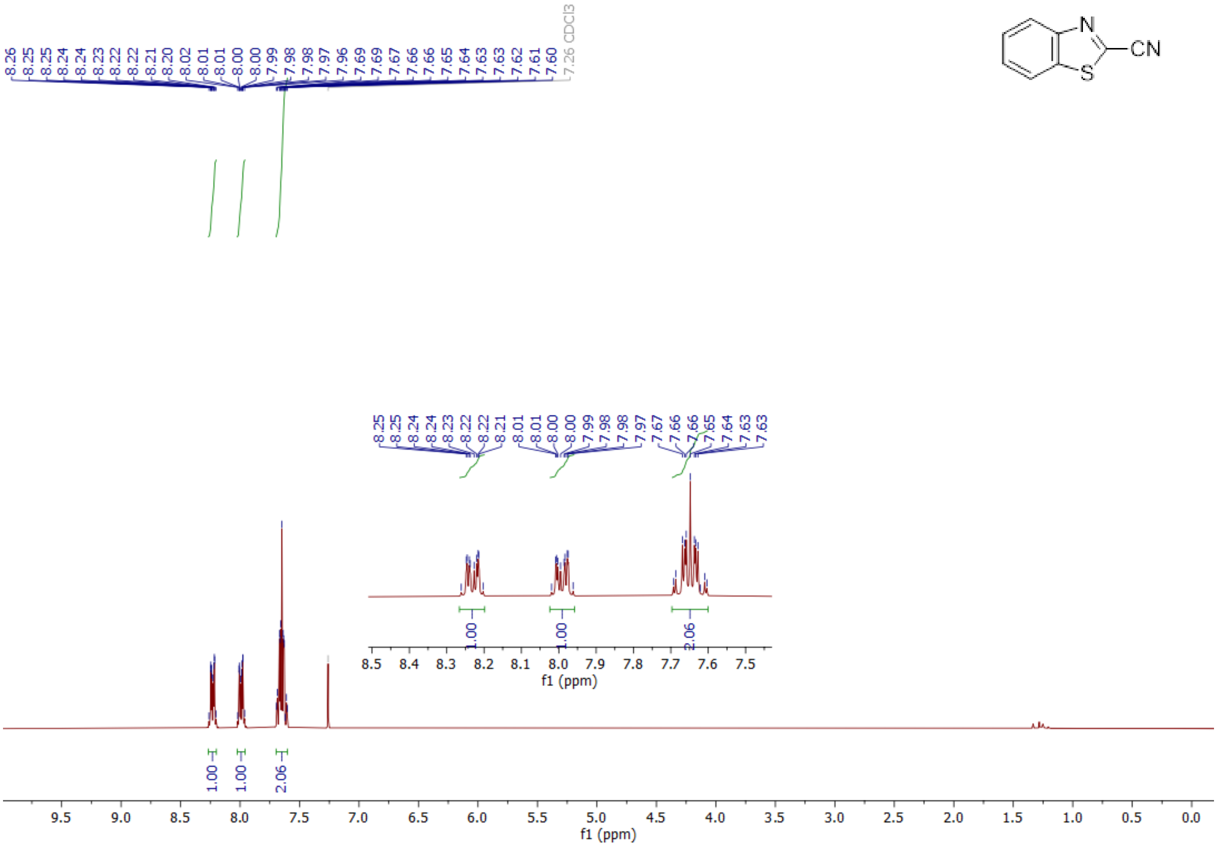
N
S
CN
8.26
8.25
8.25
8.24
8.24
8.23
8.22
8.22
8.21
8.20
8.02
8.01
8.01
8.00
8.00
7.99
7.98
7.98
7.97
7.96
7.69
7.69
7.67
7.66
7.66
7.65
7.64
7.63
7.63
7.62
7.61
7.60
7.26 CDCl3
1.00
1.00
2.06
f1 (ppm)

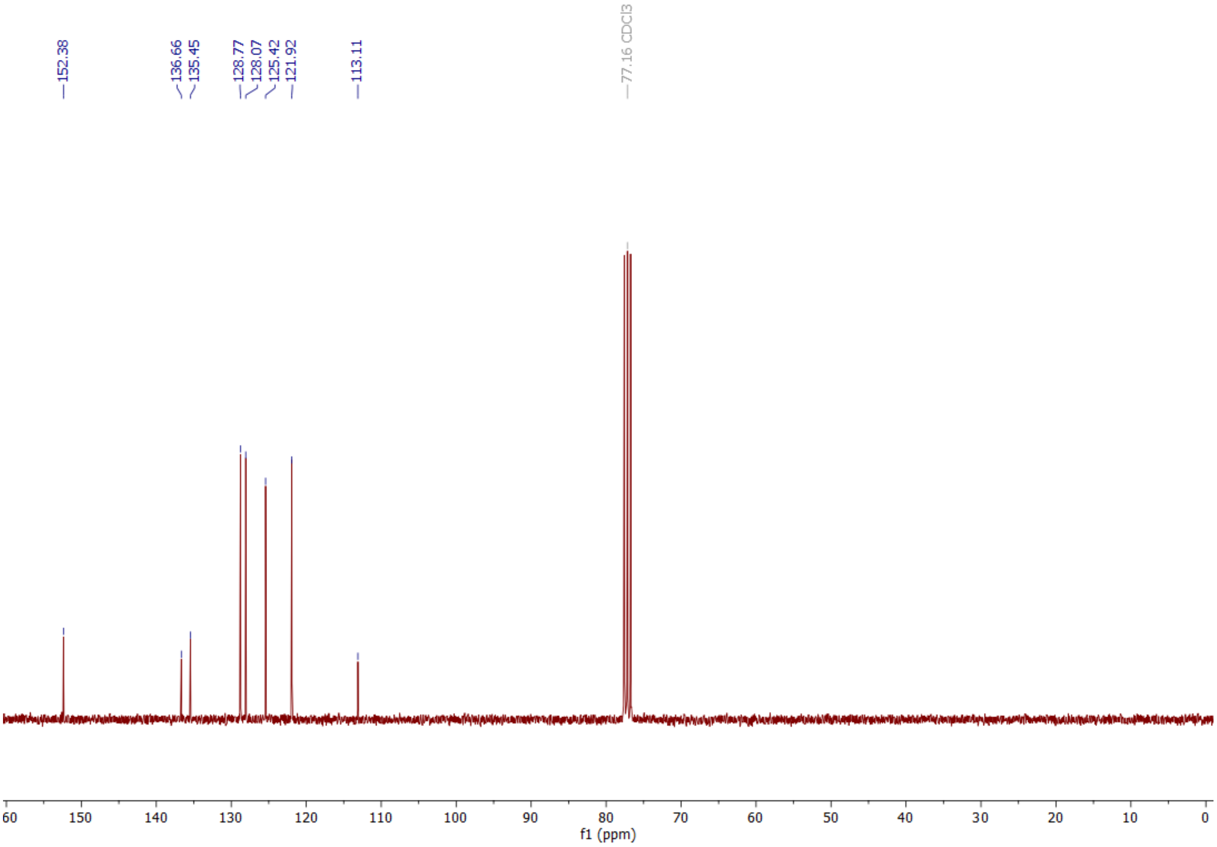
152.38
136.66
135.45
128.77
128.07
125.42
121.92
113.11
77.16 CDCl3
f1 (ppm)

4c

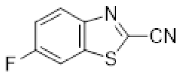

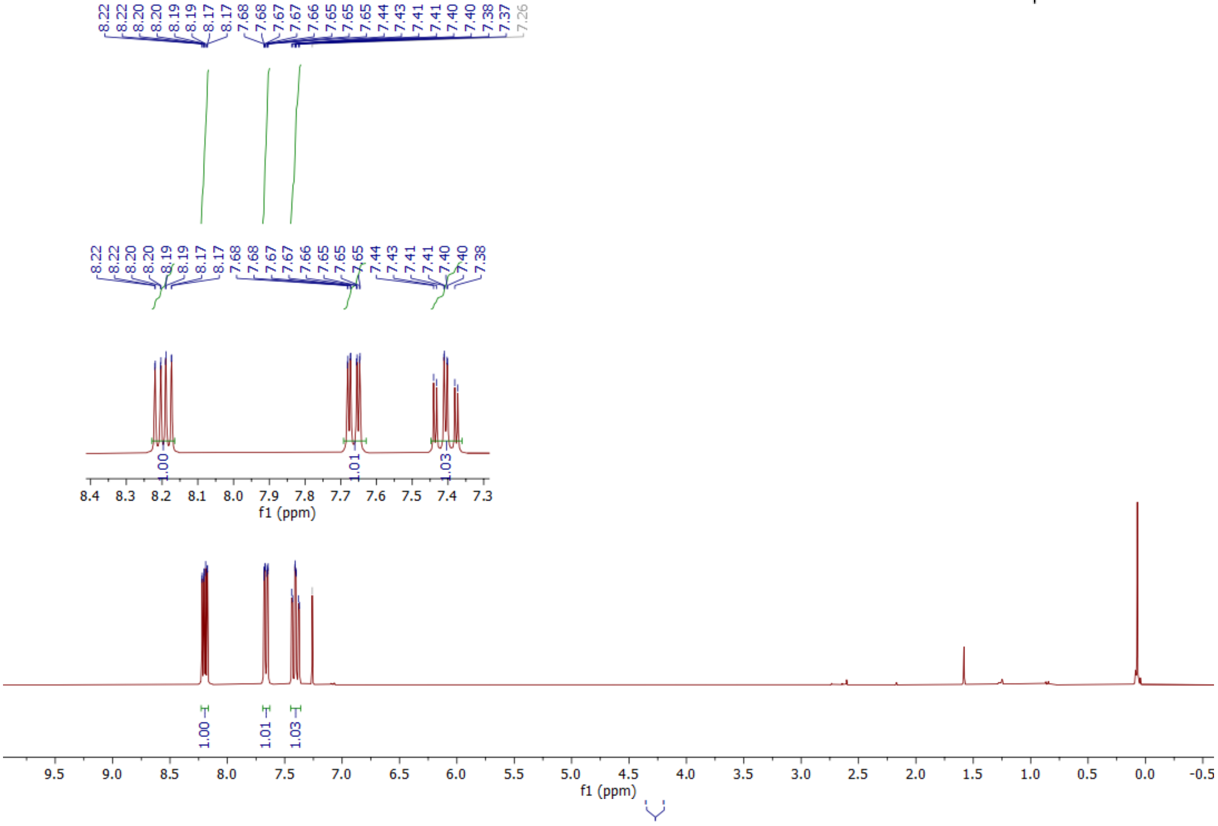

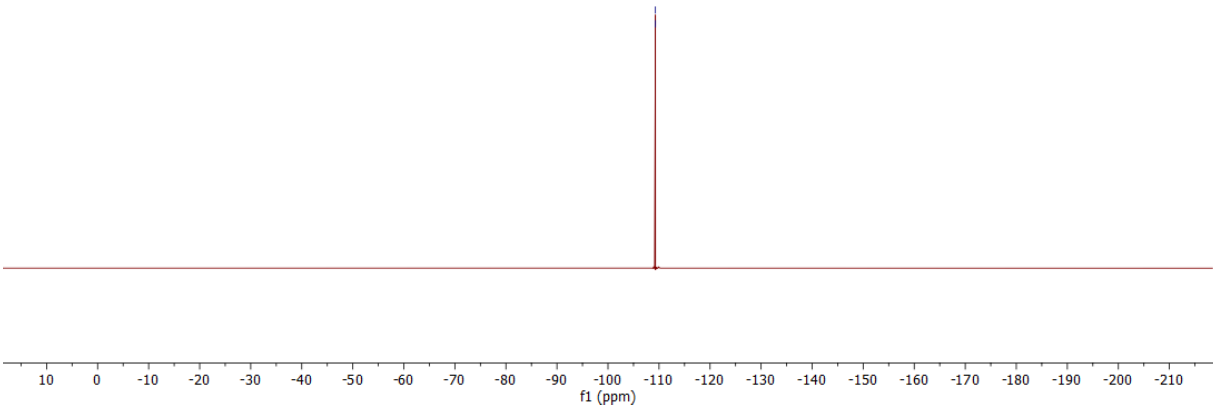

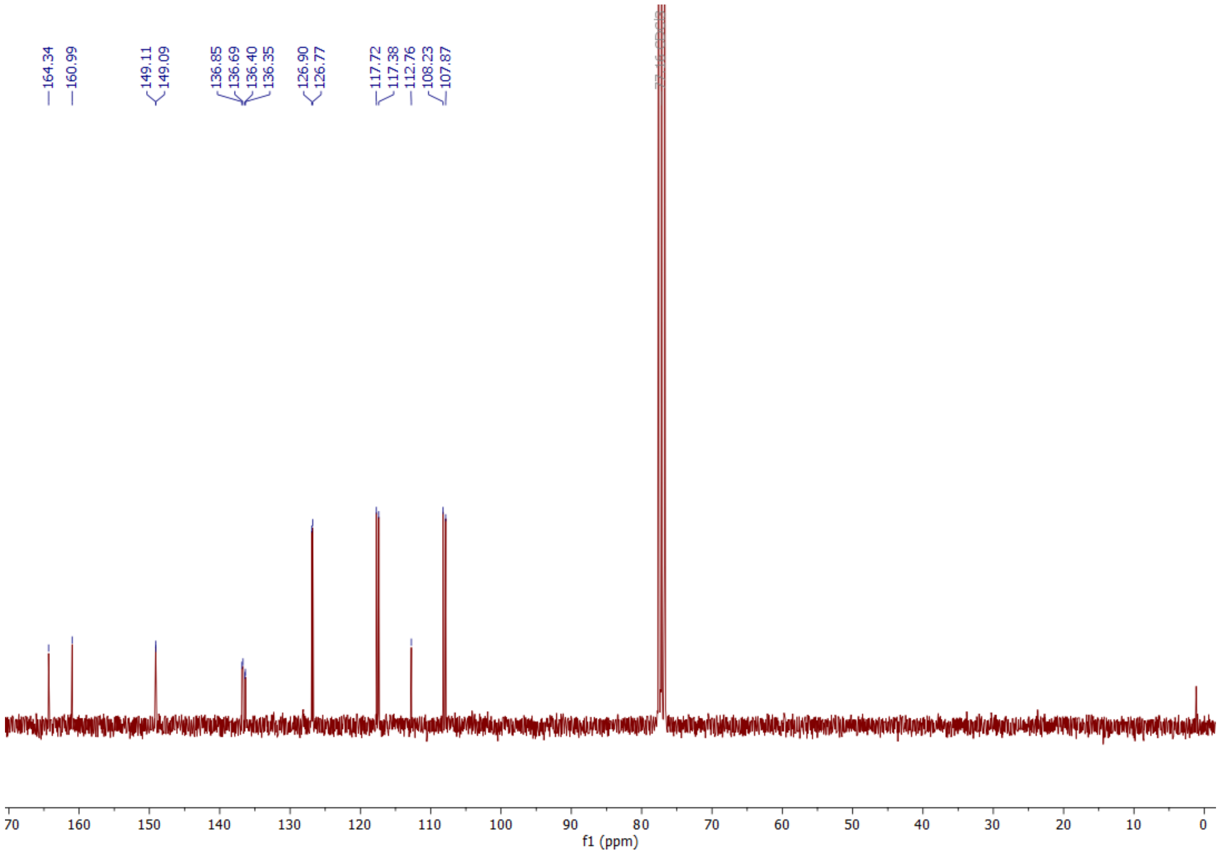
164.34
160.99
149.11
149.09
136.85
136.69
136.40
136.35
126.90
126.77
117.72
117.38
112.76
108.23
107.87
70 160 150 140 130 120 110 100 90 80 70 60 50 40 30 20 10 0
f1 (ppm)

4d

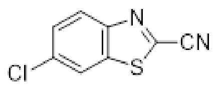

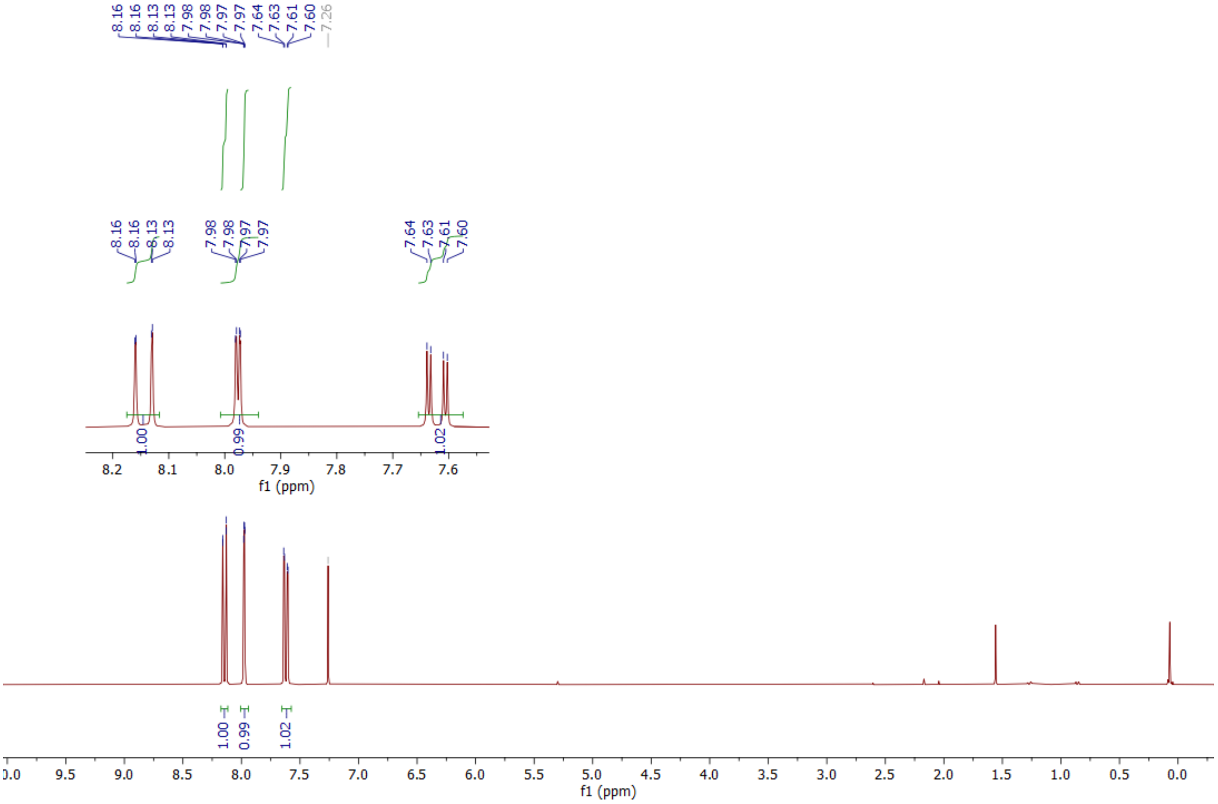

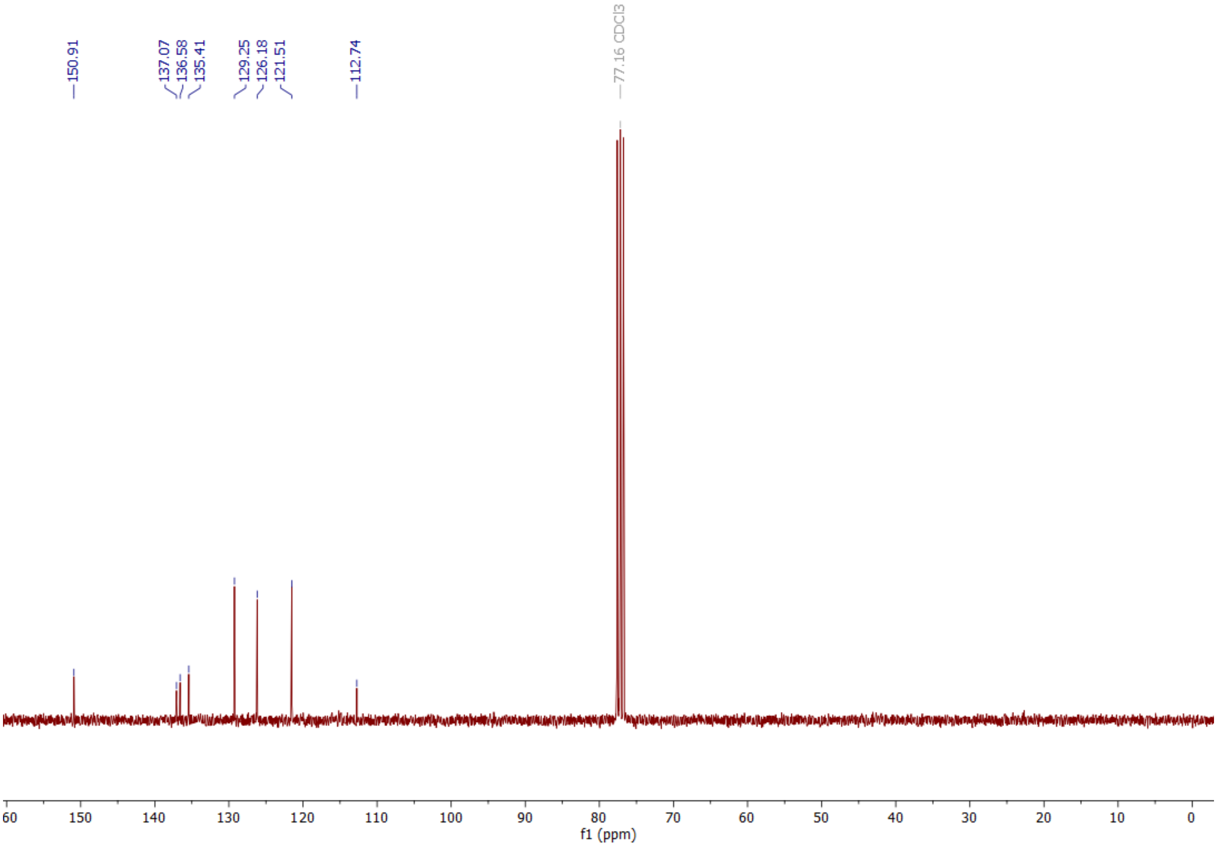

4e

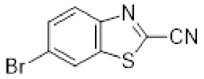

N
CN
S
Br

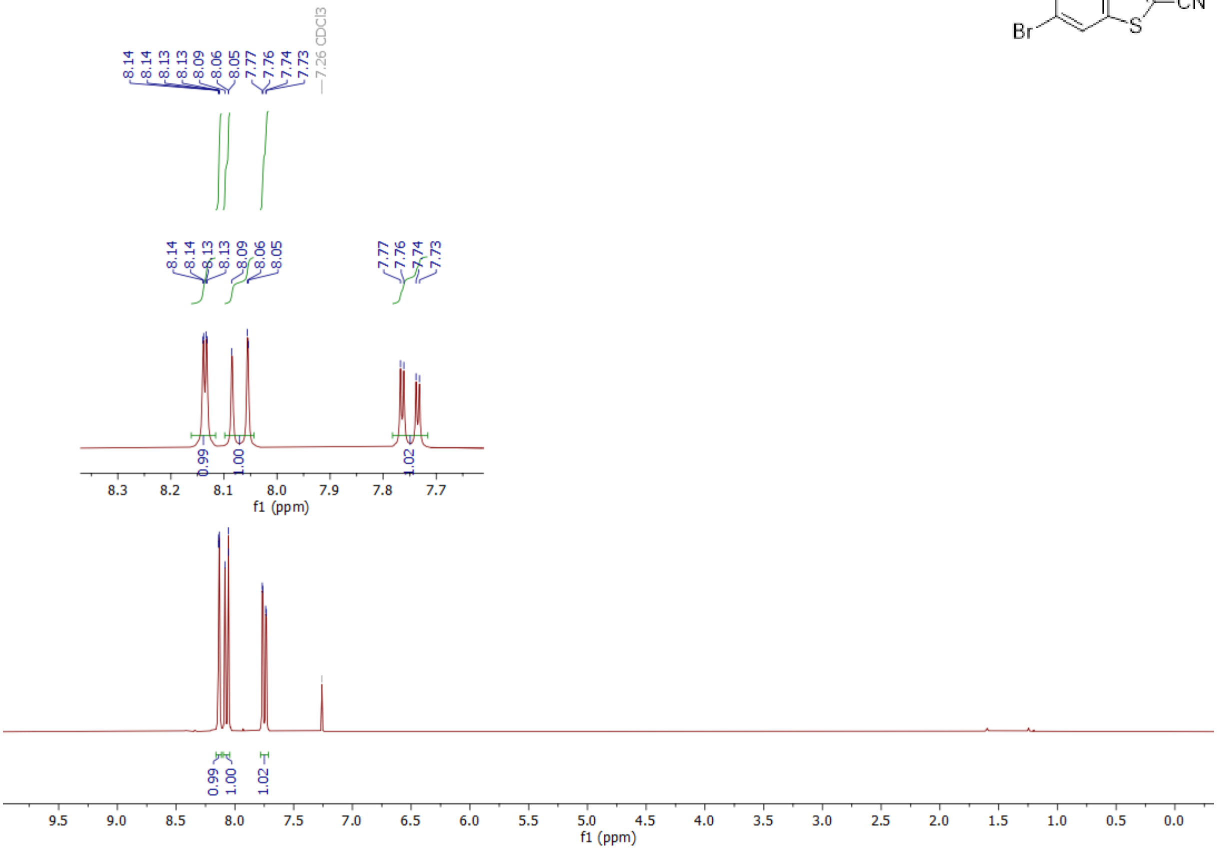

8.14
8.14
8.13
8.13
8.09
8.06
8.05
7.77
7.76
7.74
7.73
7.26 CDCl3
0.99
1.00
1.02
f1 (ppm)

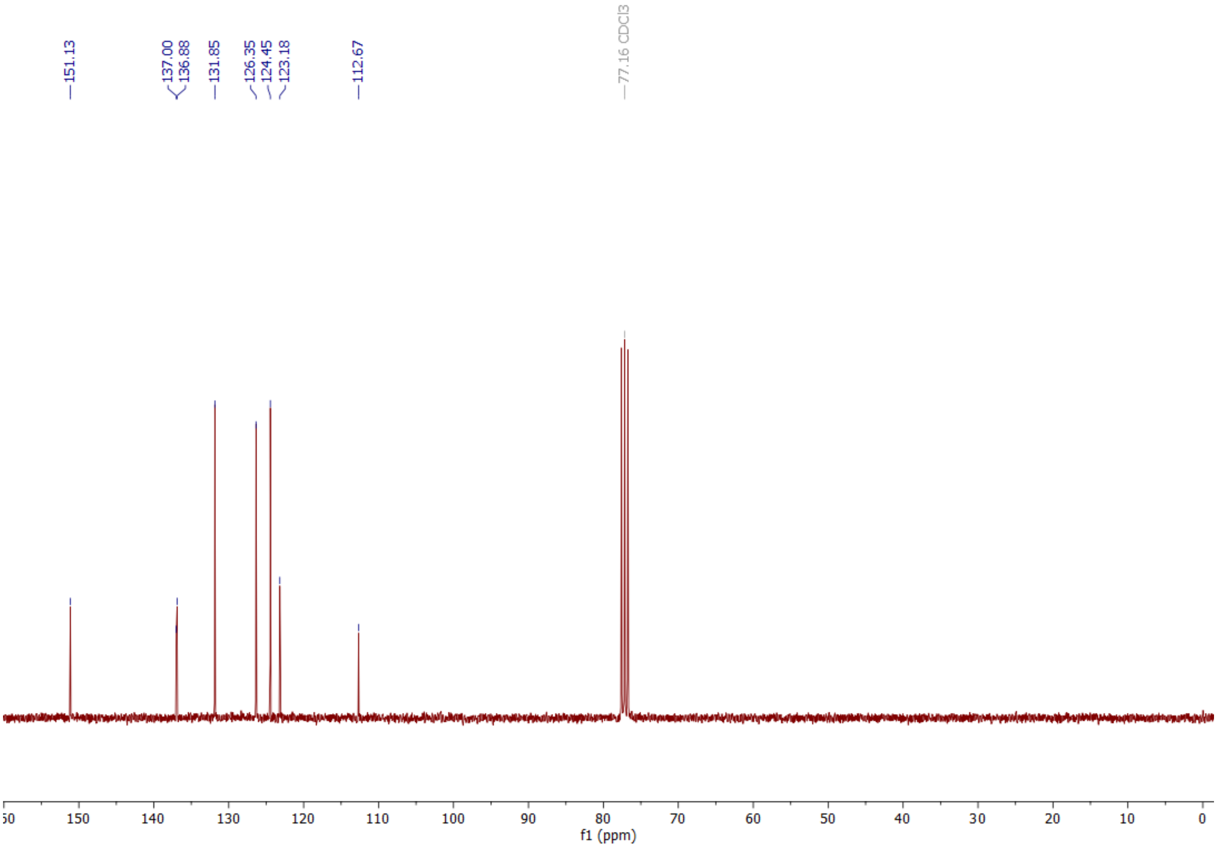

151.13
137.00
136.88
131.85
126.35
124.45
123.18
112.67
77.16 CDCl3
f1 (ppm)

4f

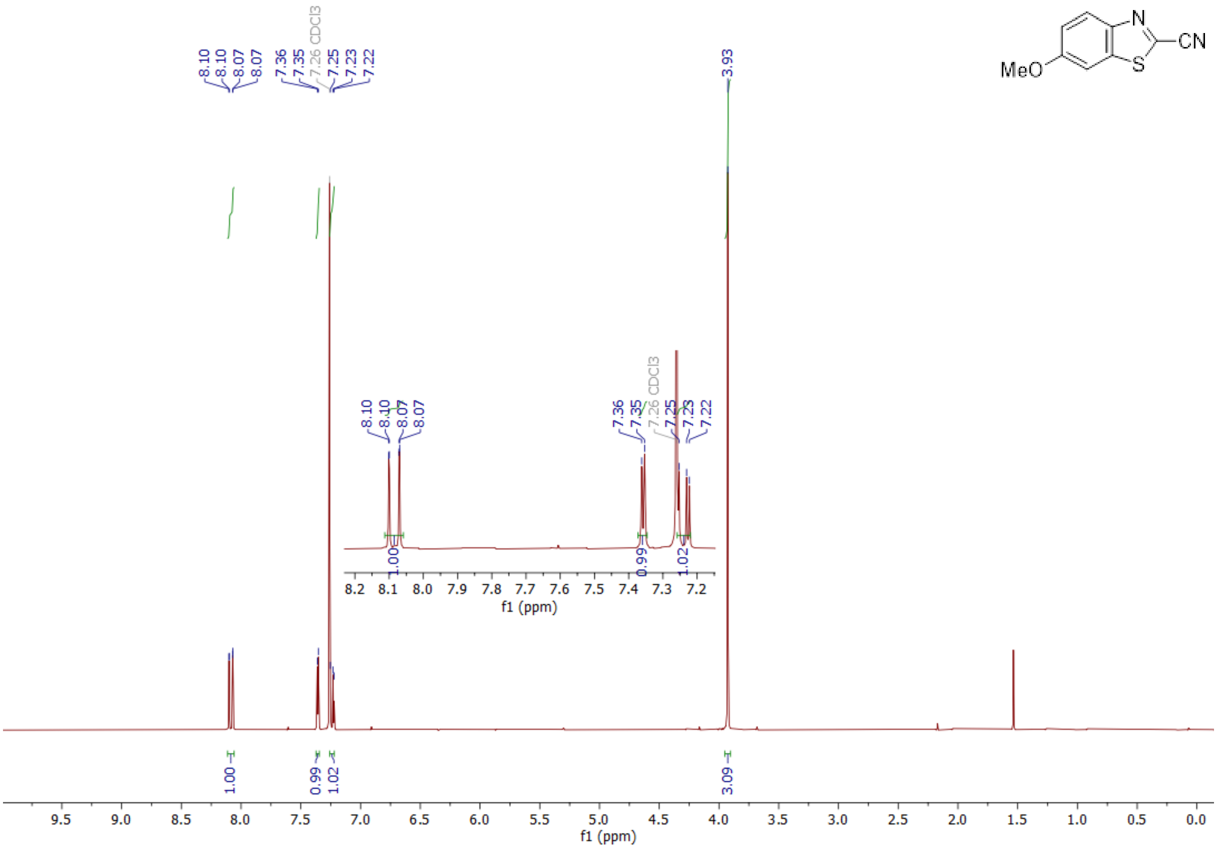

MeO
N
S
CN
8.10
8.10
8.07
8.07
7.36
7.35
7.26 CDCl3
7.25
7.23
7.22
3.93
1.00
0.99
1.02
3.09
9.5 9.0 8.5 8.0 7.5 7.0 6.5 6.0 5.5 5.0 4.5 4.0 3.5 3.0 2.5 2.0 1.5 1.0 0.5 0.0
f1 (ppm)
8.2 8.1 8.0 7.9 7.8 7.7 7.6 7.5 7.4 7.3 7.2
f1 (ppm)

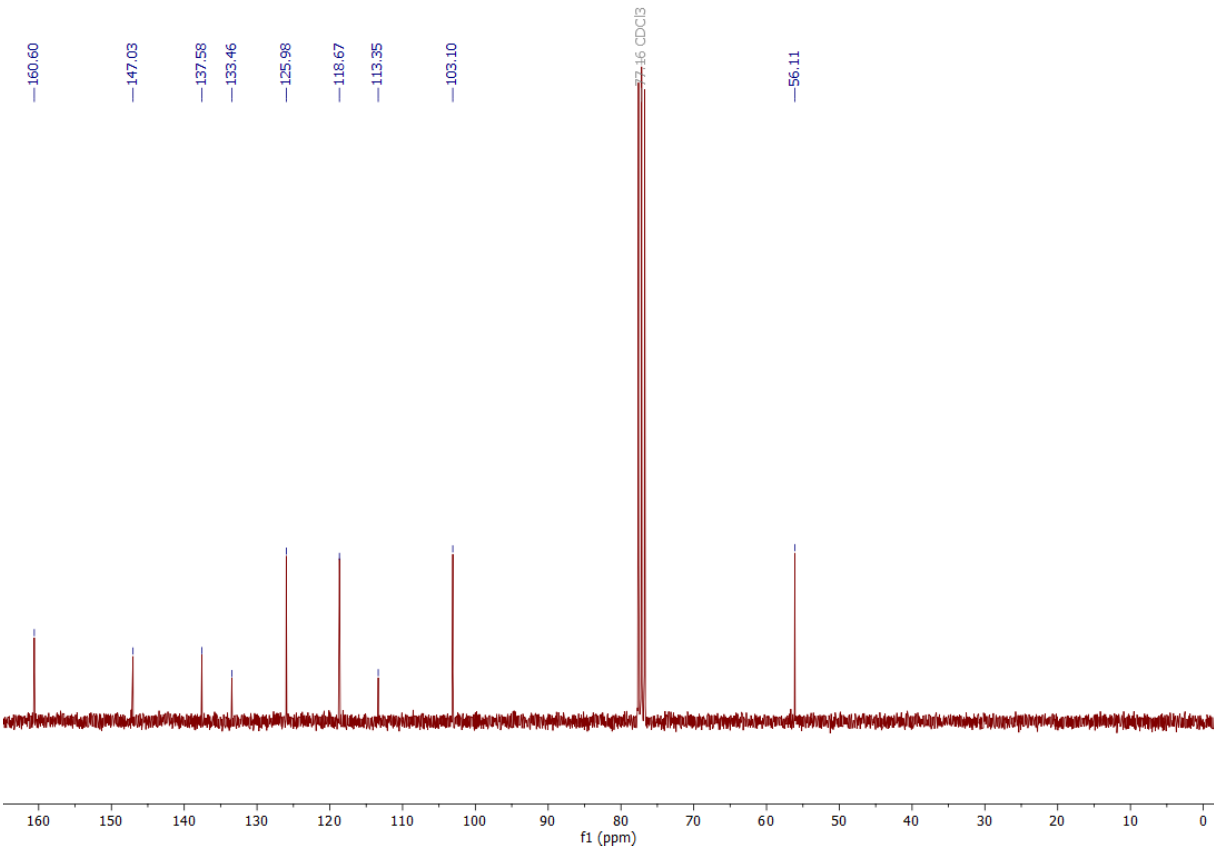

160.60
147.03
137.58
133.46
125.98
118.67
113.35
103.10
77.16 CDCl3
56.11
160 150 140 130 120 110 100 90 80 70 60 50 40 30 20 10 0
f1 (ppm)

4g

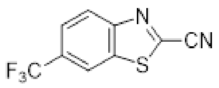

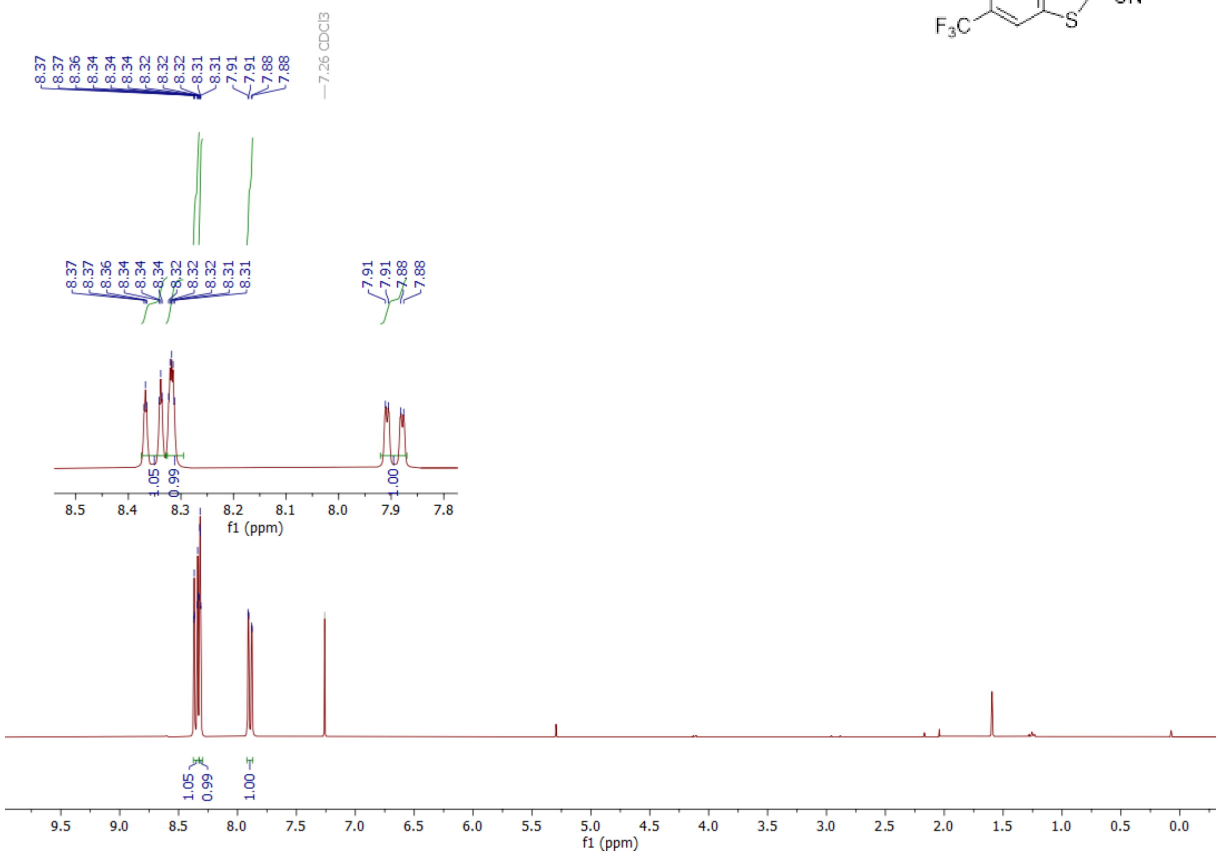

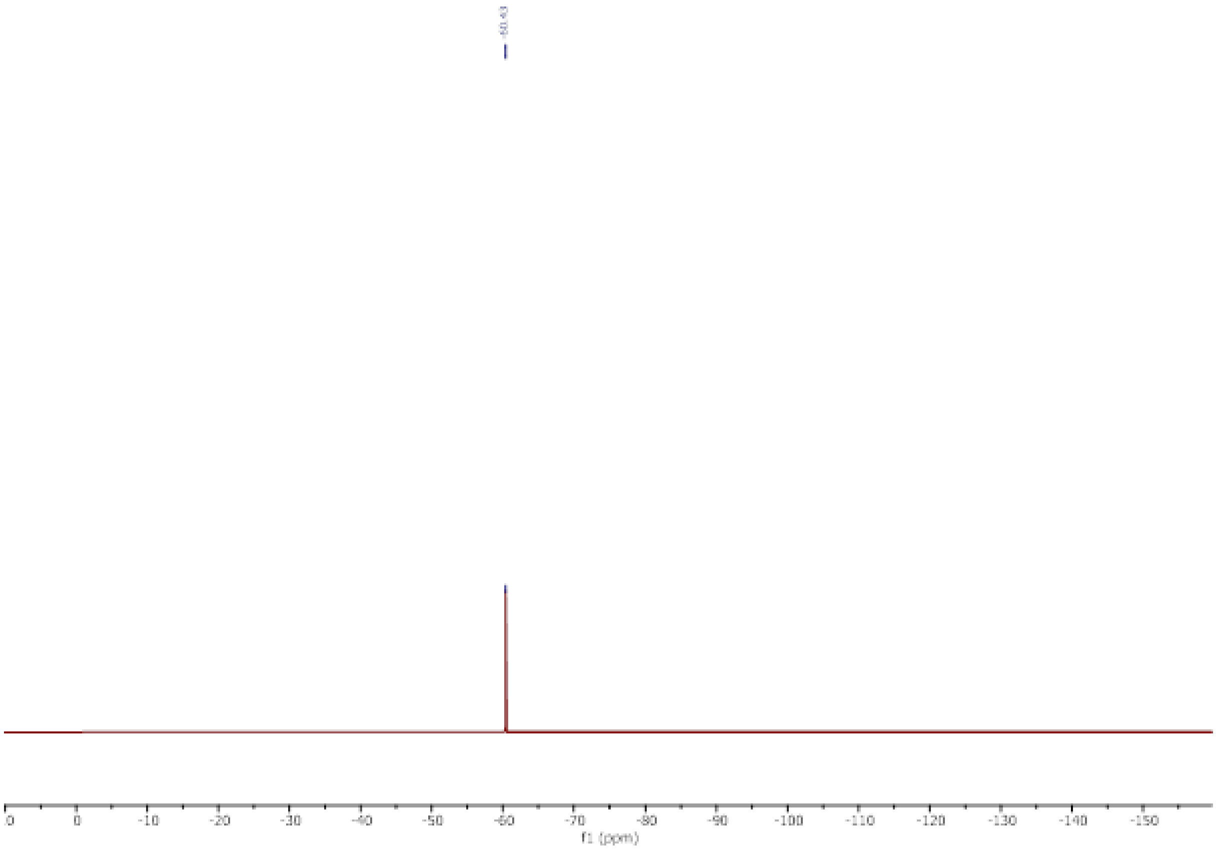

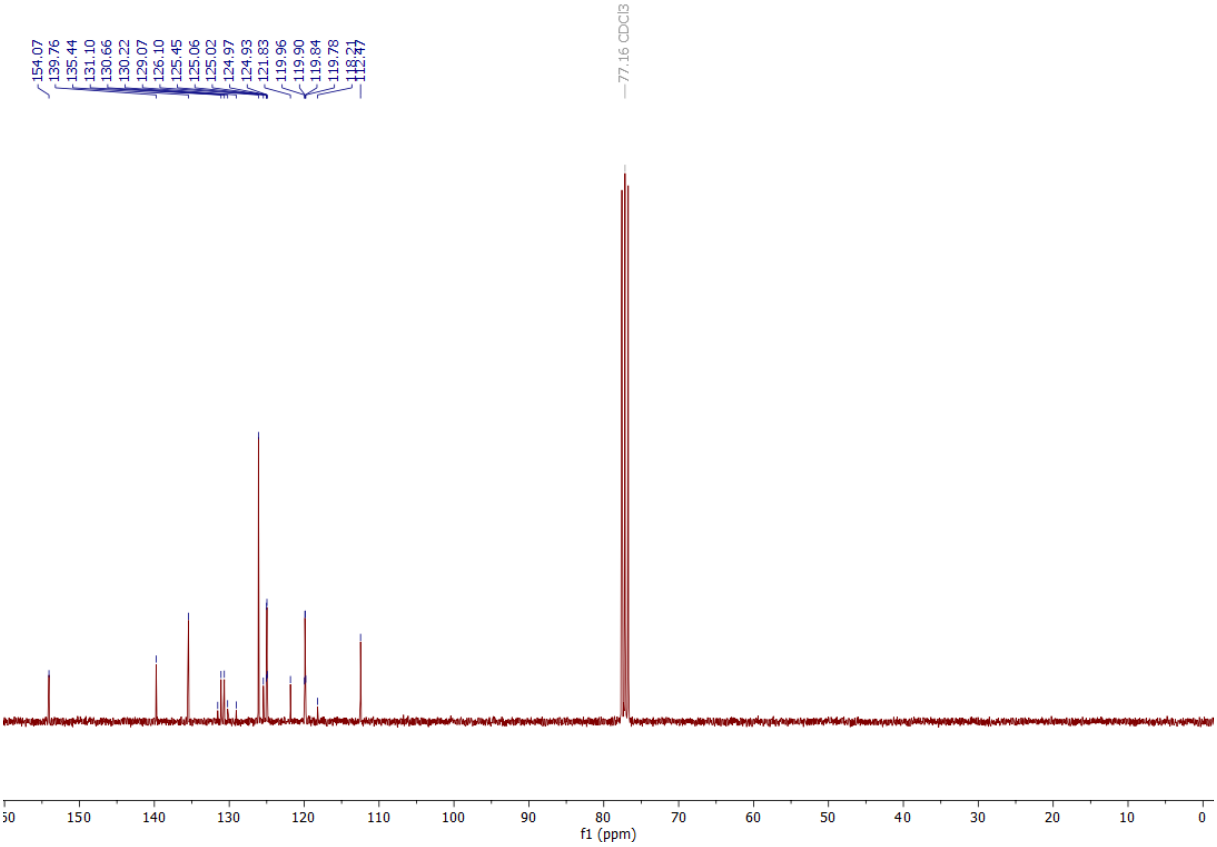
154.07
139.76
135.44
131.10
130.66
130.22
129.07
126.10
125.45
125.06
125.02
124.97
124.93
121.83
119.96
119.90
119.84
119.78
118.21
112.47
77.16 CDCl3
150
140
130
120
110
100
90
80
70
60
50
40
30
20
10
0
f1 (ppm)

4h

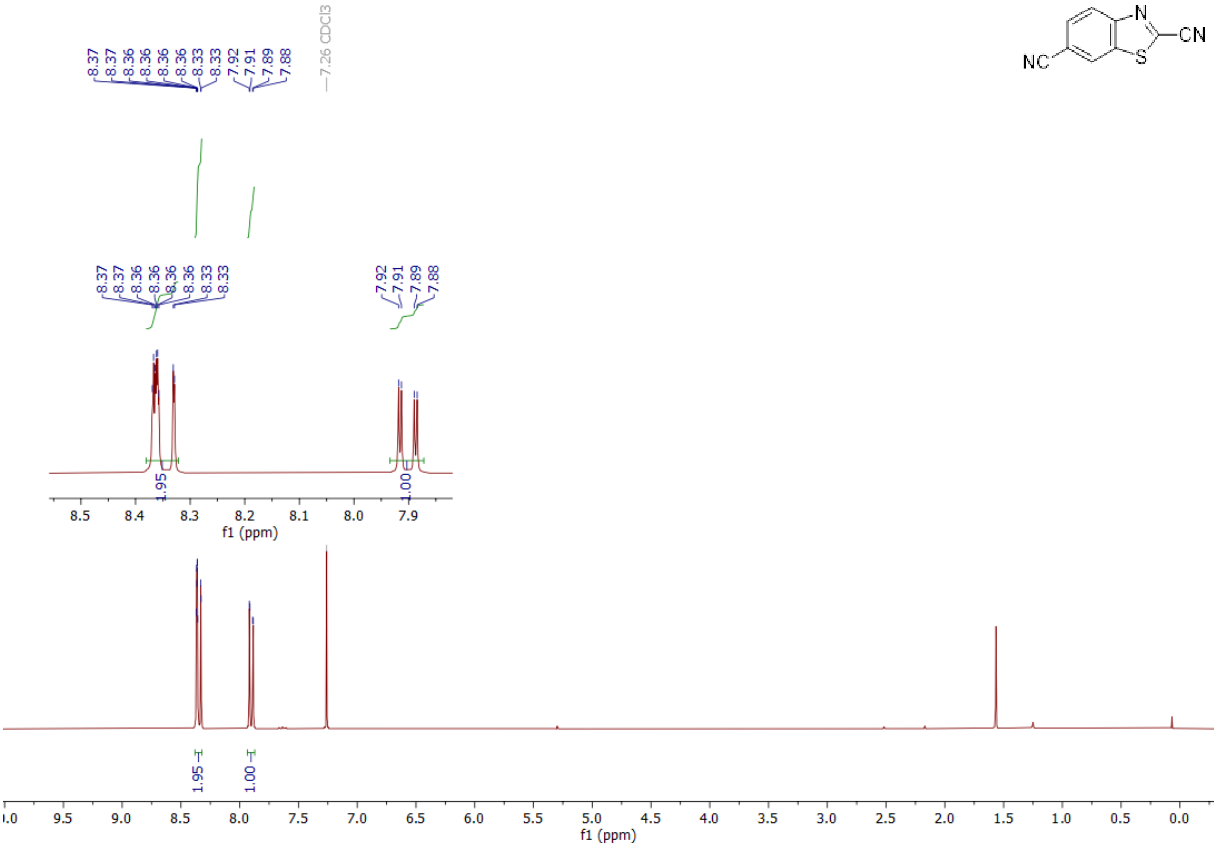

NC
N
S
CN
8.37
8.37
8.36
8.36
8.36
8.36
8.33
8.33
7.92
7.91
7.89
7.88
7.26 CDCl3
1.95
1.00
f1 (ppm)

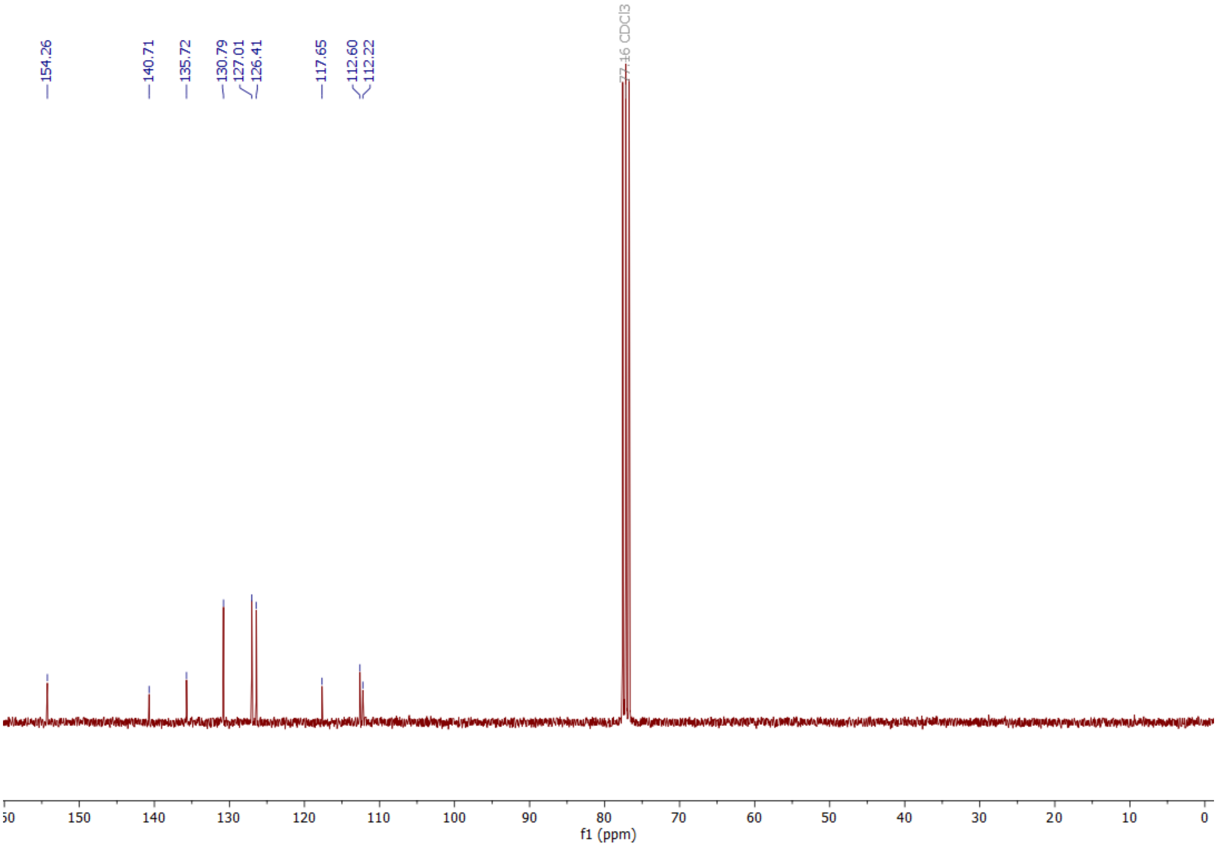

154.26
140.71
135.72
130.79
127.01
126.41
117.65
112.60
112.22
77.16 CDCl3
f1 (ppm)

4i

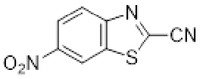
N
CN
O_2N
S

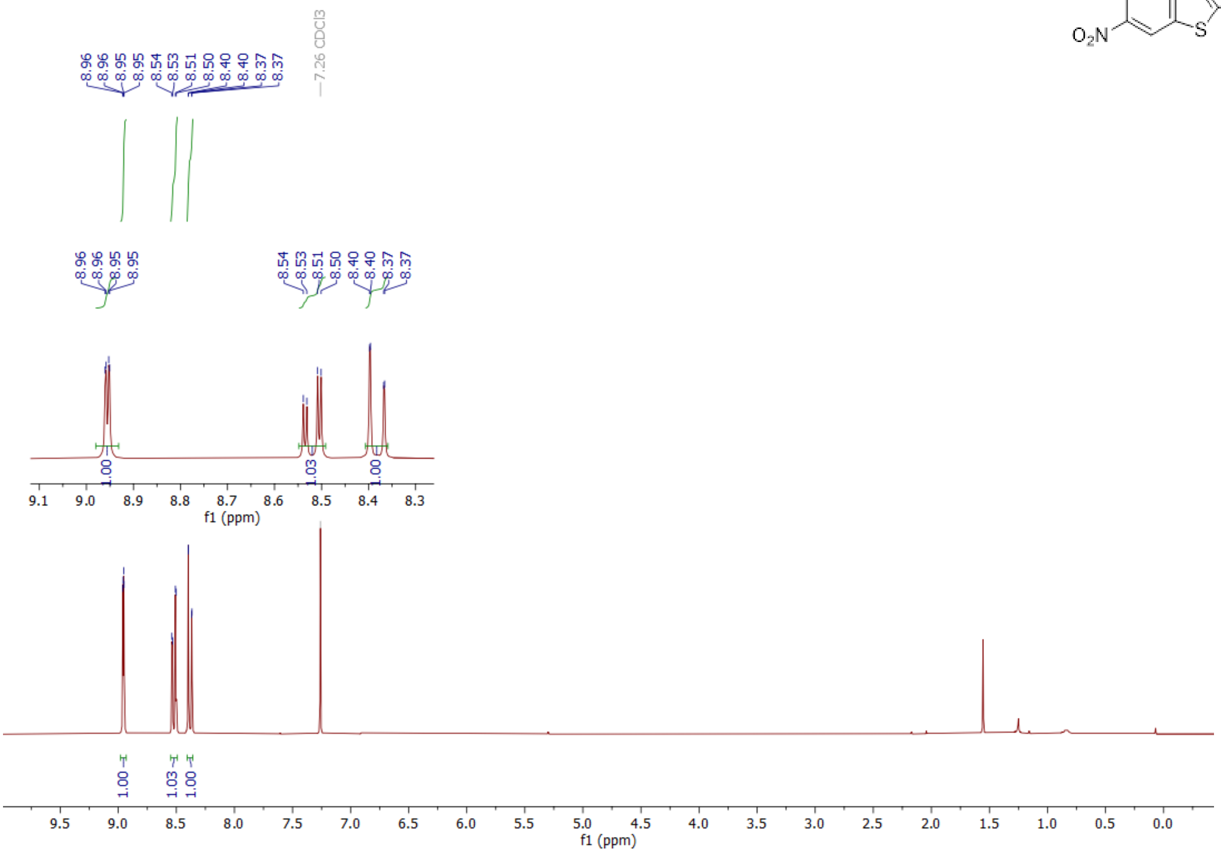
8.96
8.96
8.95
8.95
8.54
8.53
8.51
8.50
8.40
8.40
8.37
8.37
7.26 CDCl3
1.00
1.03
1.00
9.1 9.0 8.9 8.8 8.7 8.6 8.5 8.4 8.3
f1 (ppm)
9.5 9.0 8.5 8.0 7.5 7.0 6.5 6.0 5.5 5.0 4.5 4.0 3.5 3.0 2.5 2.0 1.5 1.0 0.5 0.0
f1 (ppm)

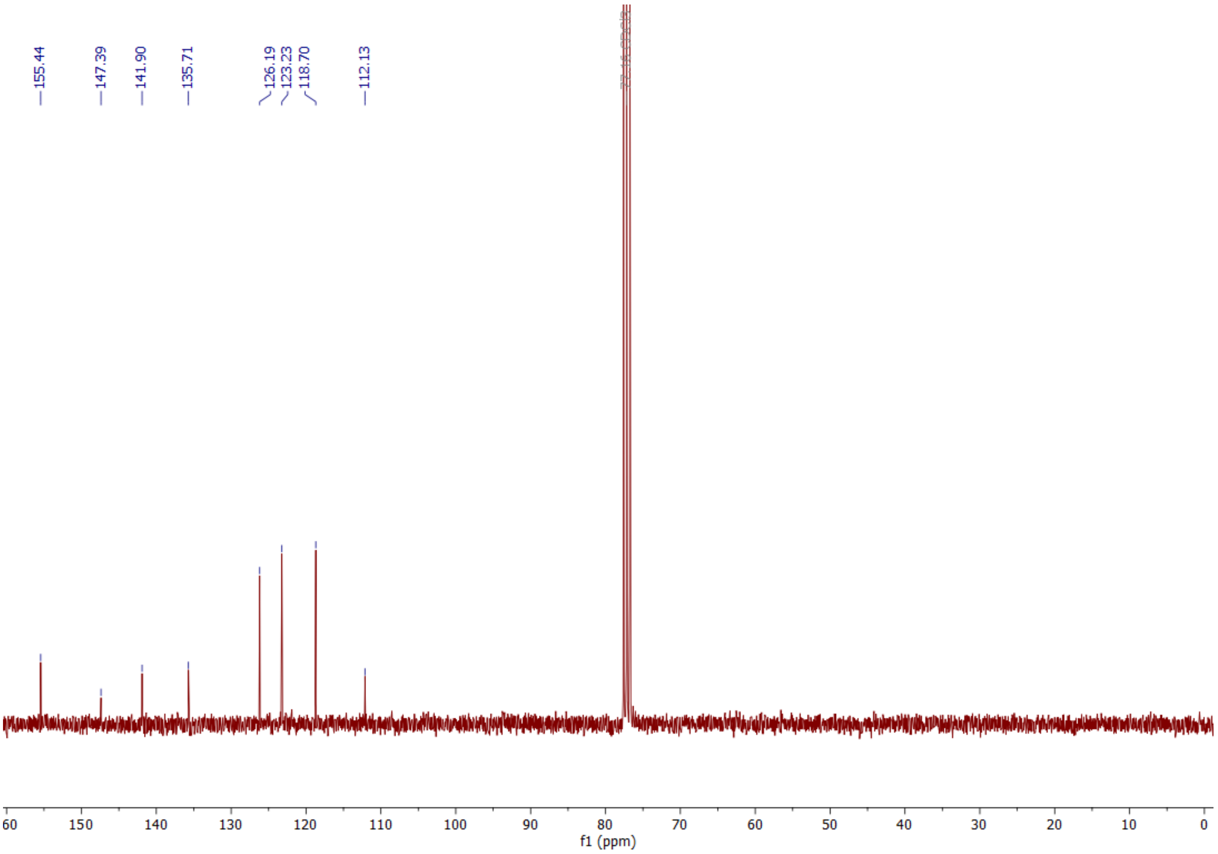
155.44
147.39
141.90
135.71
126.19
123.23
118.70
112.13
60 150 140 130 120 110 100 90 80 70 60 50 40 30 20 10 0
f1 (ppm)

4j

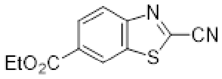

EtO2C
N
S
CN

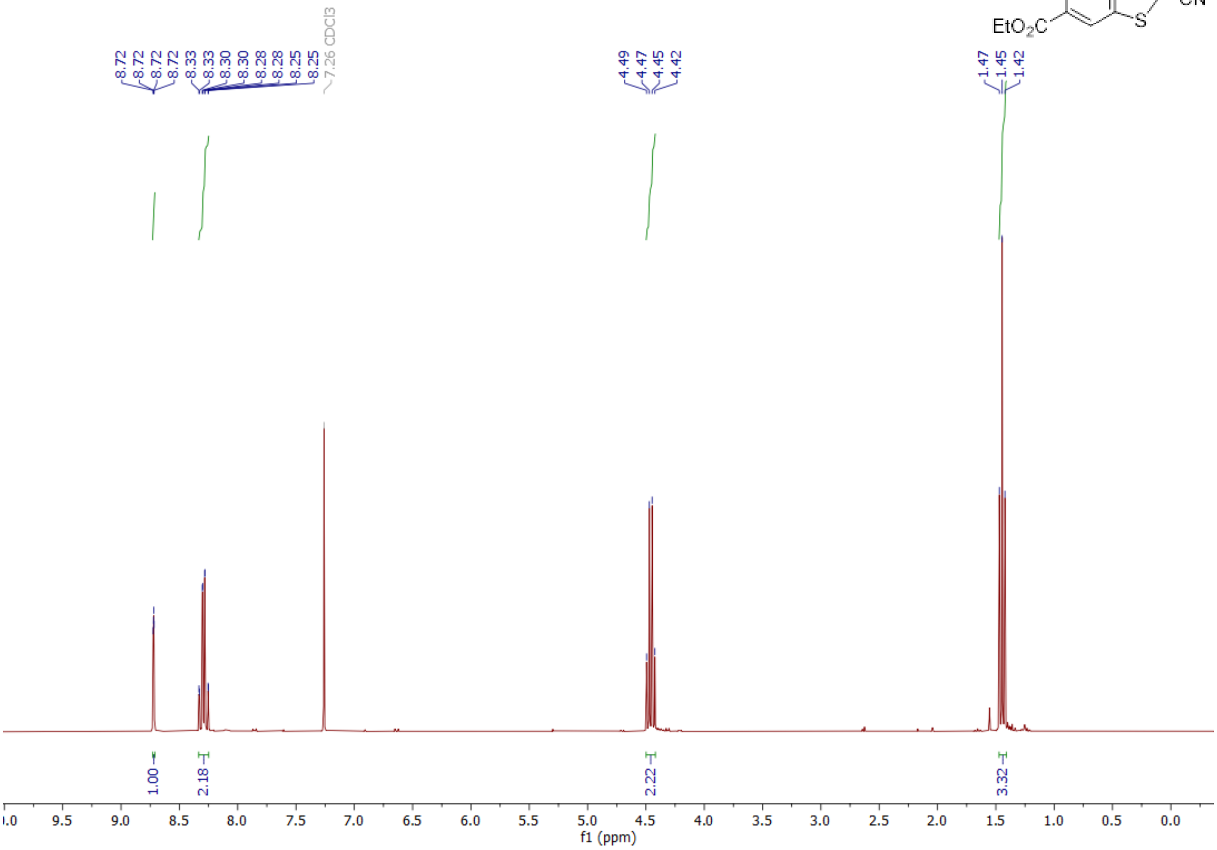

8.72
8.72
8.72
8.72
8.33
8.33
8.30
8.30
8.28
8.28
8.25
8.25
7.26 CDCl3
4.49
4.47
4.45
4.42
1.47
1.45
1.42
1.00
2.18
2.22
3.32
f1 (ppm)

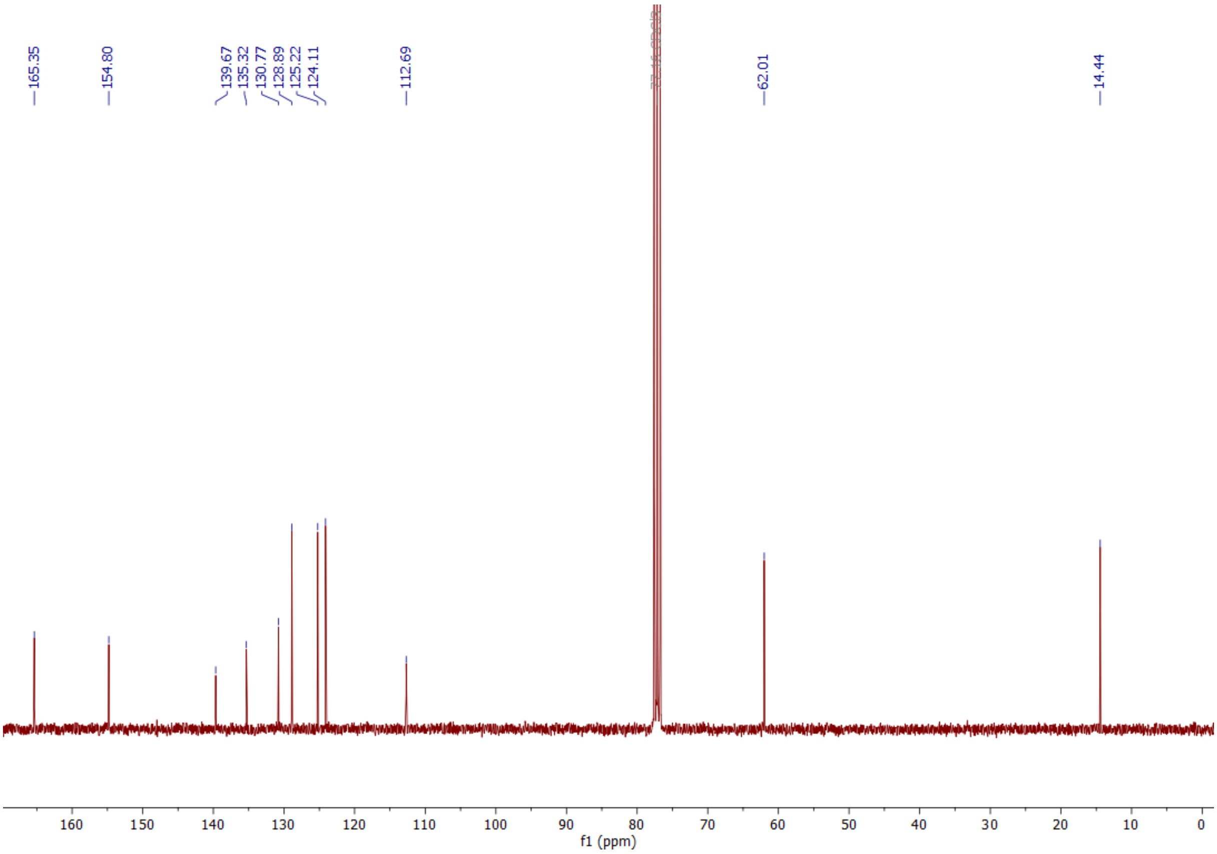

165.35
154.80
139.67
135.32
130.77
128.89
125.22
124.11
112.69
62.01
14.44
f1 (ppm)

4k

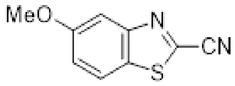
MeO
N
S
CN

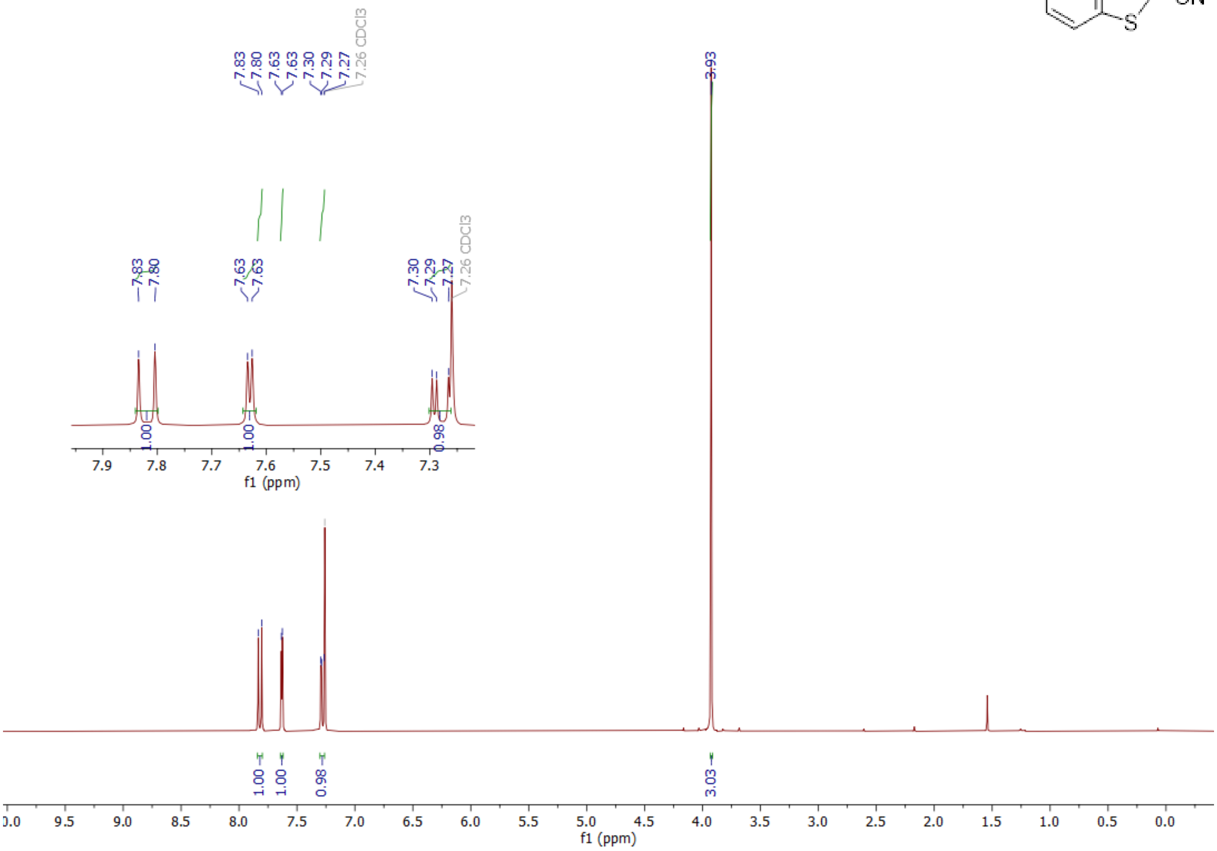
7.83
7.80
7.63
7.63
7.30
7.29
7.27
7.26 CDCl3
3.93
1.00
1.00
0.98
3.03
f1 (ppm)

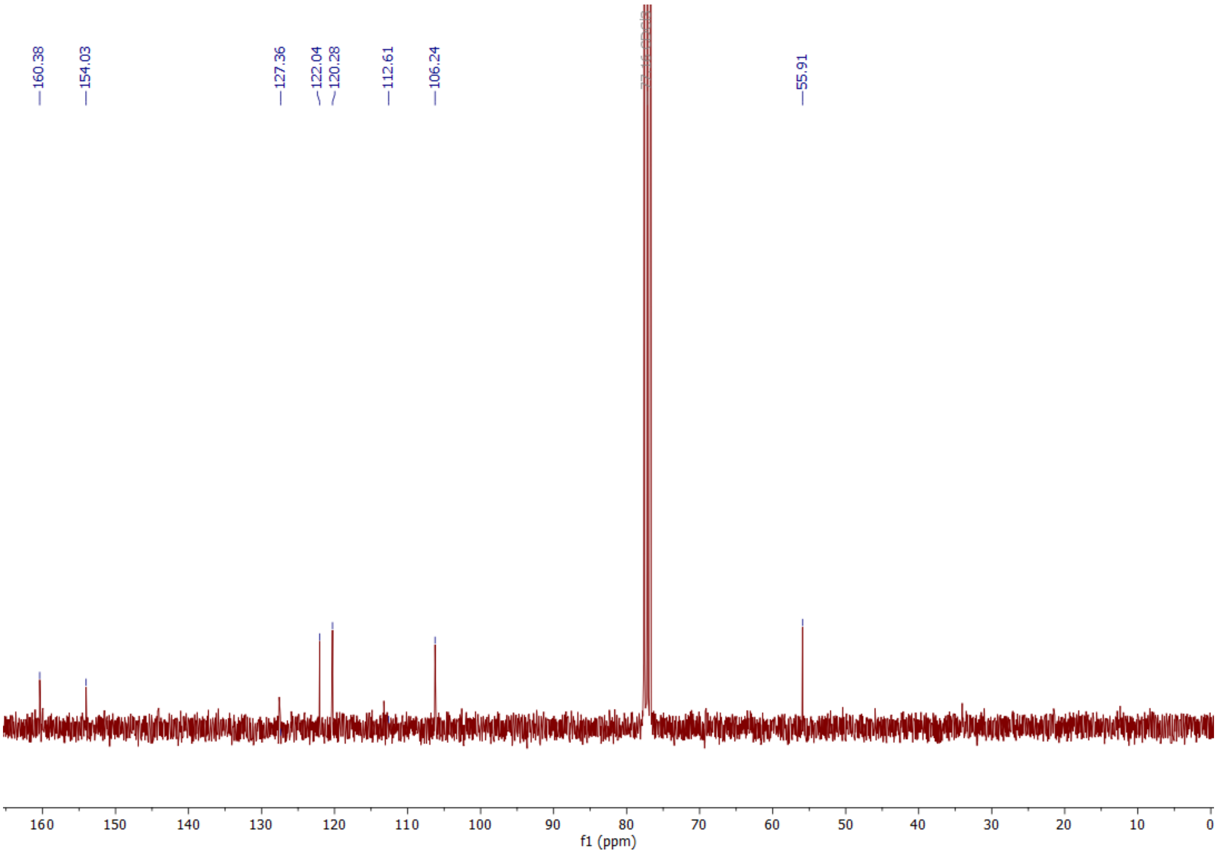
160.38
154.03
127.36
122.04
120.28
112.61
106.24
55.91
f1 (ppm)

4l

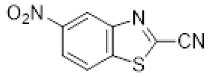

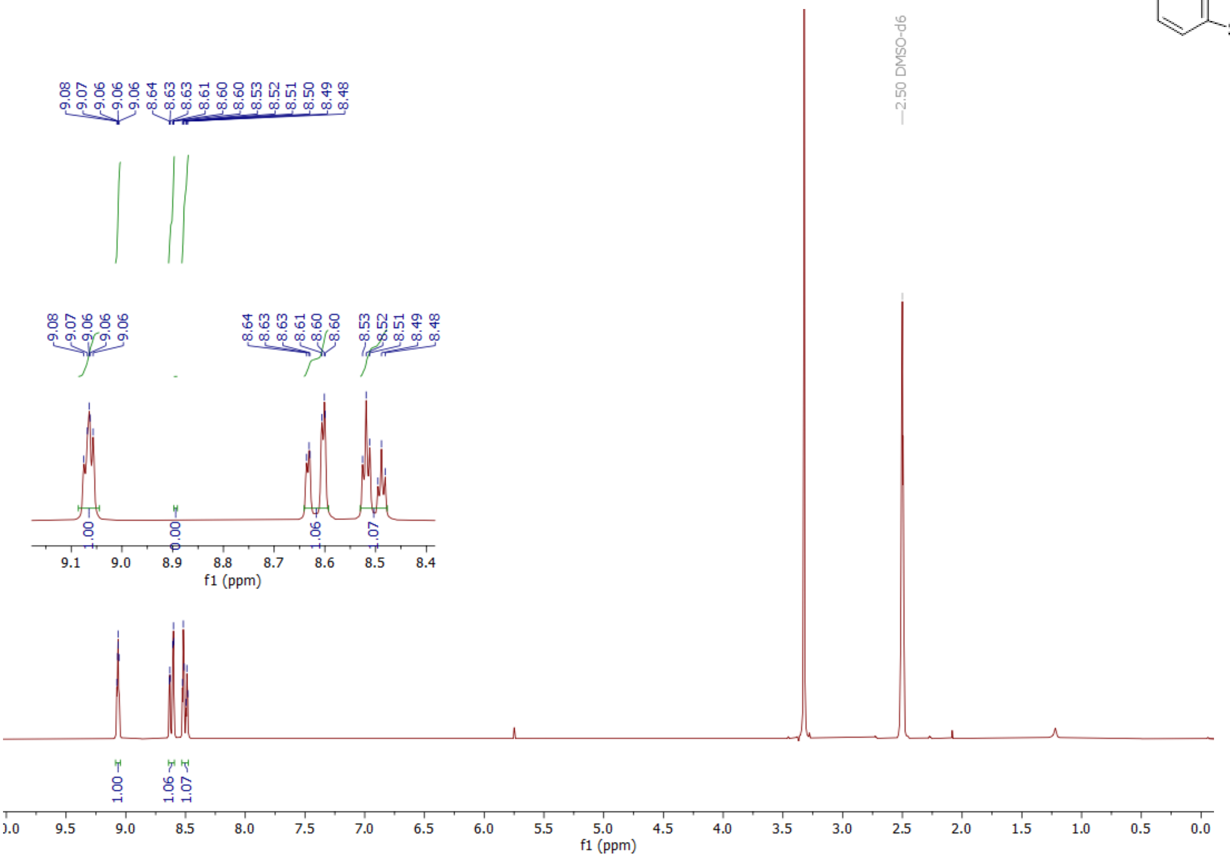

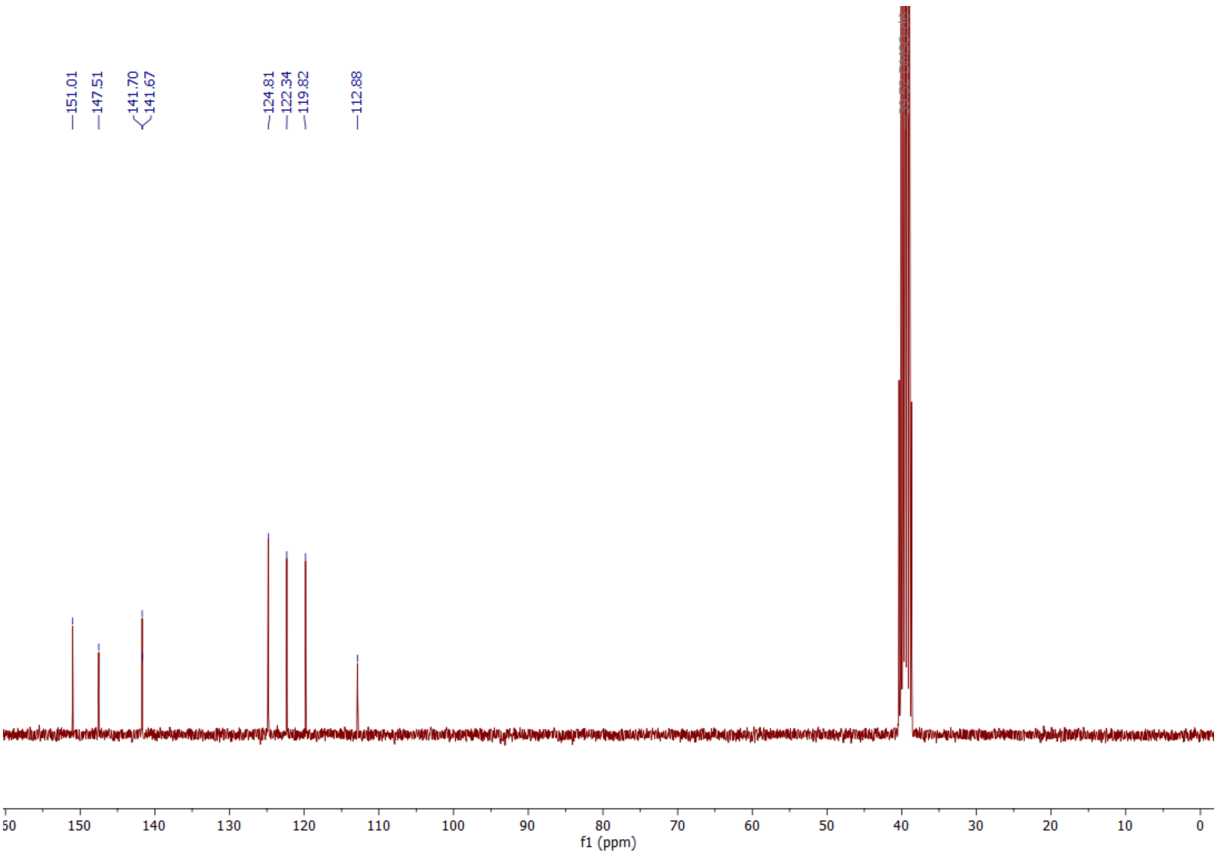

4m

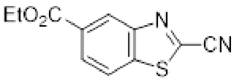
EtO2C
N
CN
S

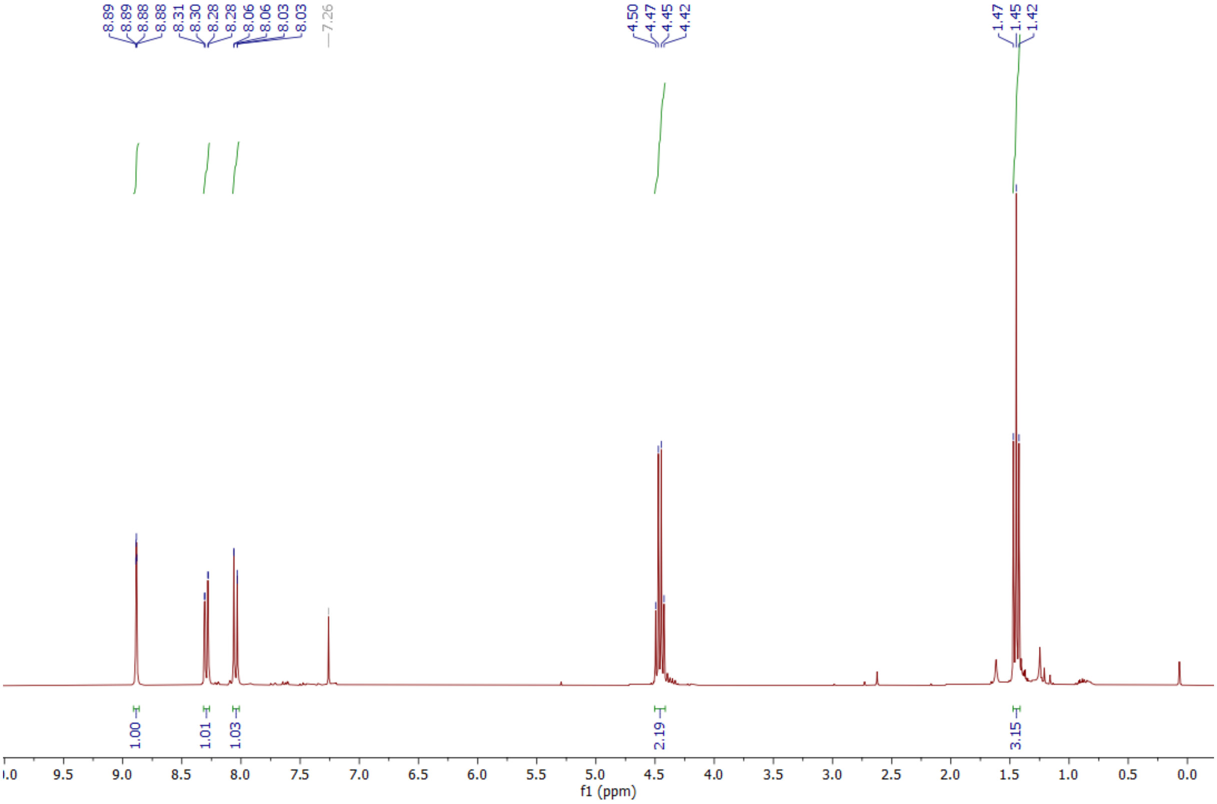
8.89
8.89
8.88
8.88
8.31
8.30
8.28
8.28
8.06
8.06
8.03
8.03
7.26 CDCl3
4.50
4.47
4.45
4.42
1.47
1.45
1.42
1.00
1.01
1.03
2.19
3.15
f1 (ppm)

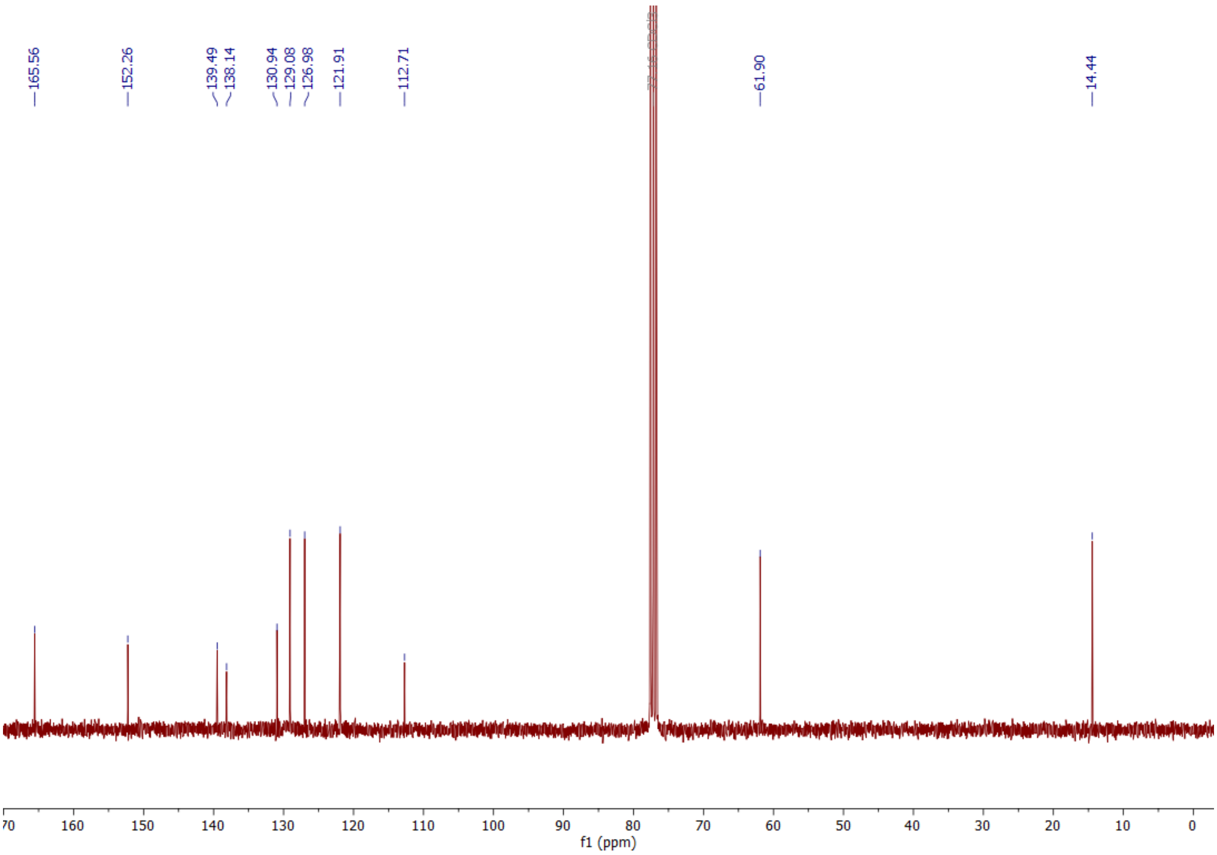
165.56
152.26
139.49
138.14
130.94
129.08
126.98
121.91
112.71
61.90
14.44
f1 (ppm)

4n

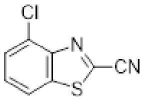
Cl
N
S
CN

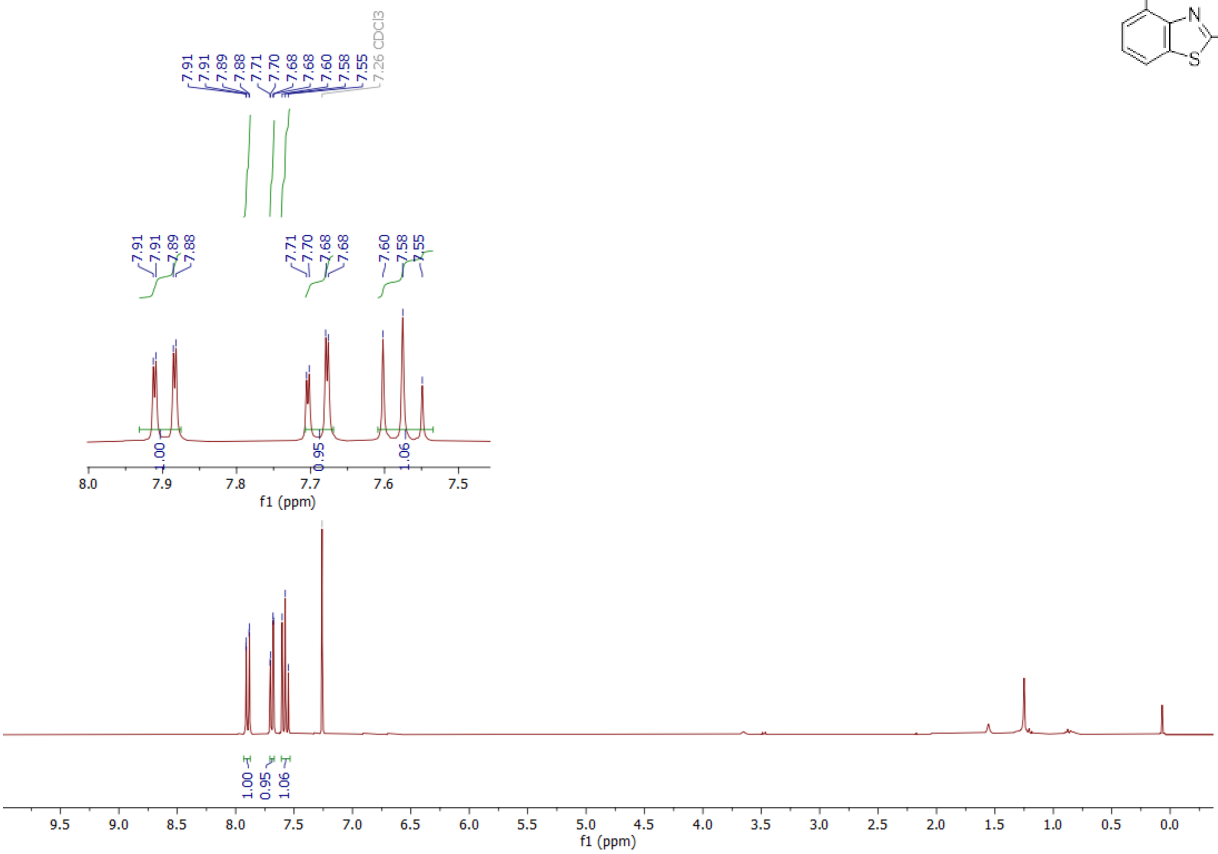
7.91
7.91
7.89
7.88
7.71
7.70
7.68
7.68
7.60
7.58
7.55
7.26 CDCl3
1.00
0.95
1.06
f1 (ppm)

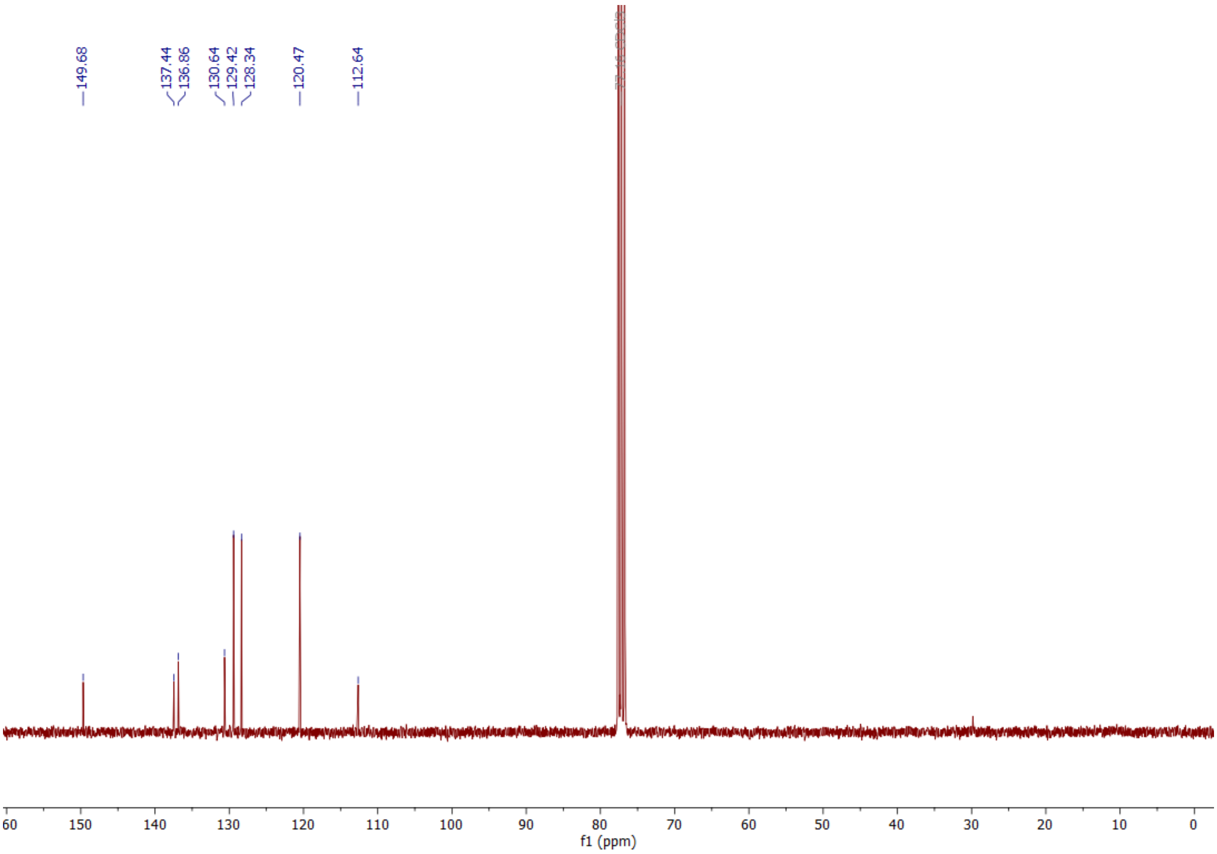
149.68
137.44
136.86
130.64
129.42
128.34
120.47
112.64
f1 (ppm)

4o

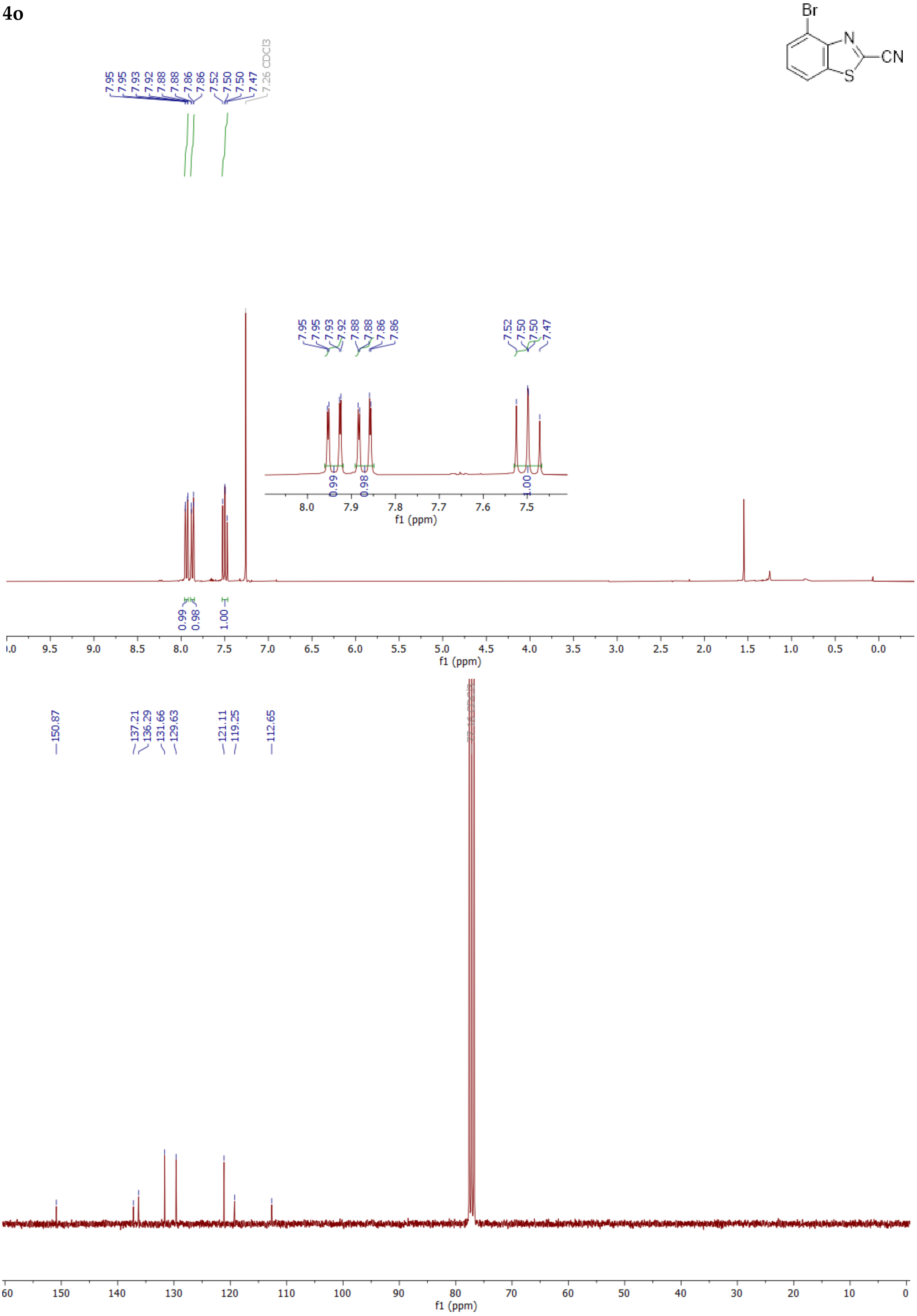
Br
N
CN
S
7.95
7.95
7.93
7.92
7.88
7.88
7.86
7.86
7.52
7.50
7.50
7.47
7.26 CDCl3
0.99
0.98
1.00
f1 (ppm)
150.87
137.21
136.29
131.66
129.63
121.11
119.25
112.65

4p

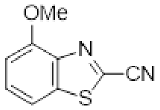
OMe
N
CN
S

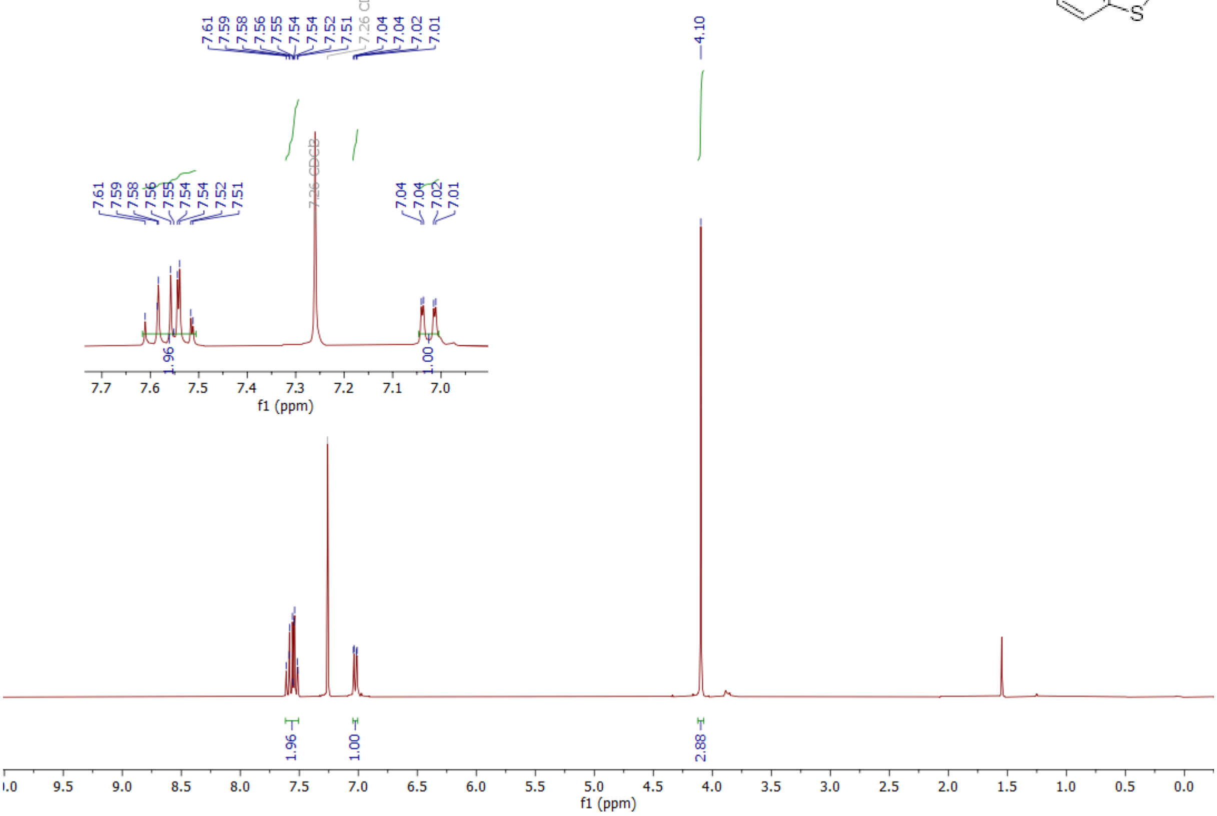
7.61
7.59
7.58
7.56
7.55
7.54
7.54
7.52
7.51
7.26 CDCl3
7.04
7.04
7.02
7.01
4.10
1.96
1.00
2.88
f1 (ppm)

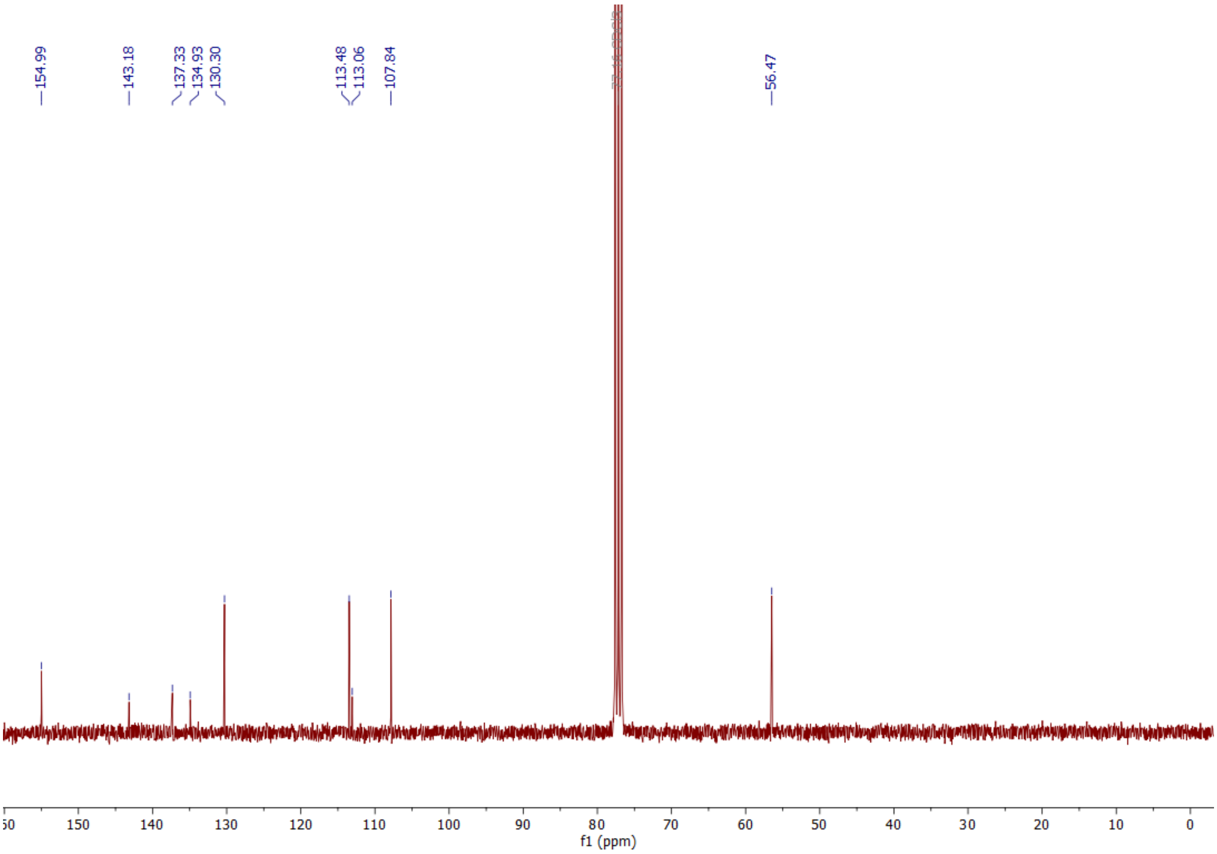
154.99
143.18
137.33
134.93
130.30
113.48
113.06
107.84
56.47
f1 (ppm)

4q

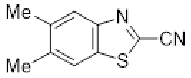
Me
Me
N
S
CN

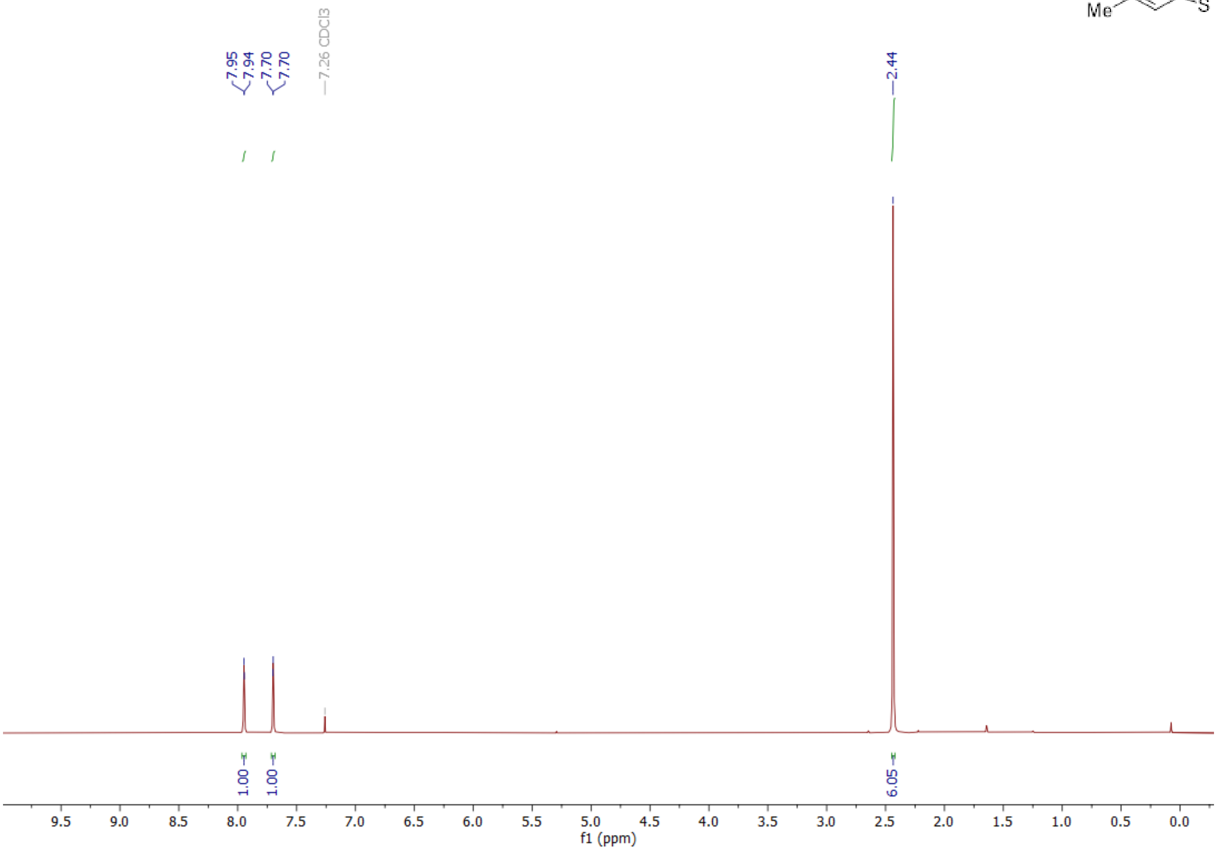
7.95
7.94
7.70
7.70
7.26 CDCl3
2.44
1.00
1.00
6.05
f1 (ppm)

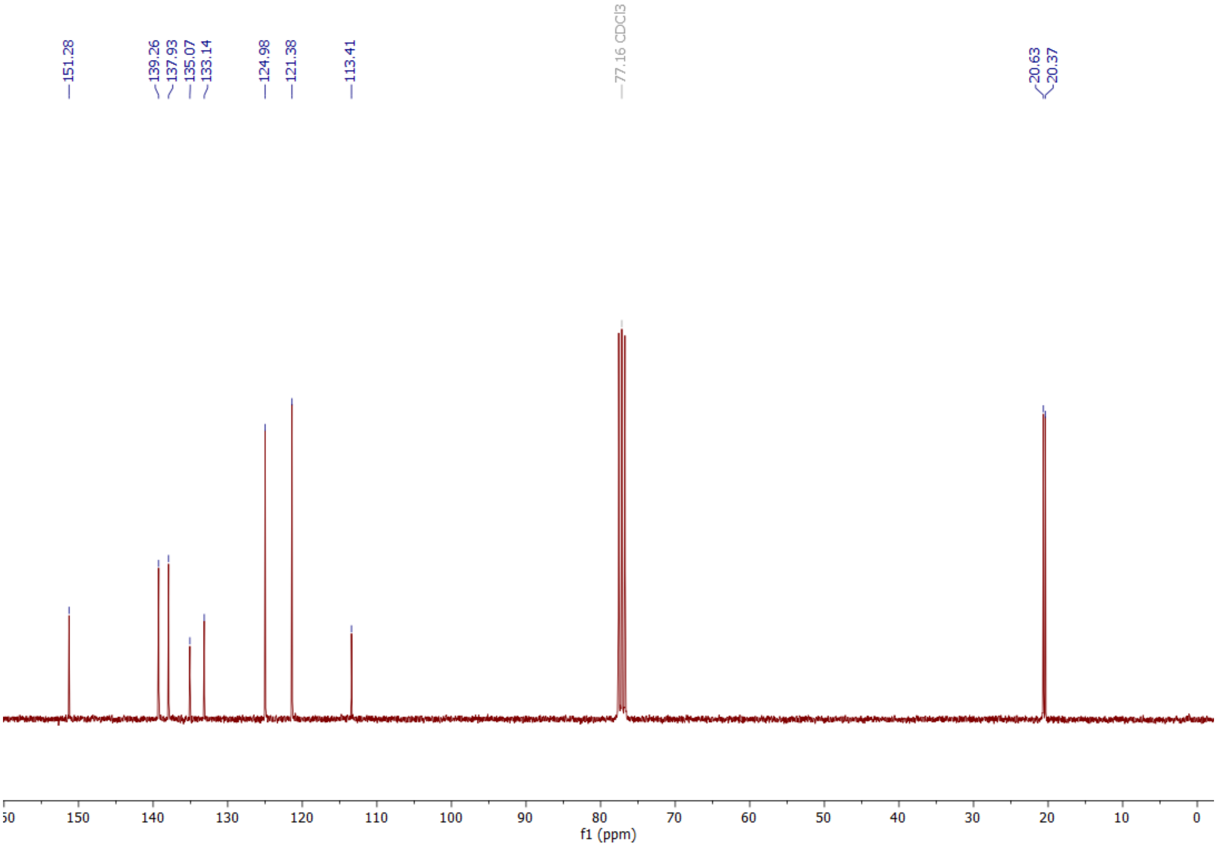
151.28
139.26
137.93
135.07
133.14
124.98
121.38
113.41
77.16 CDCl3
20.63
20.37
f1 (ppm)

4r

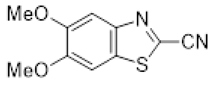
MeO
MeO
N
S
CN

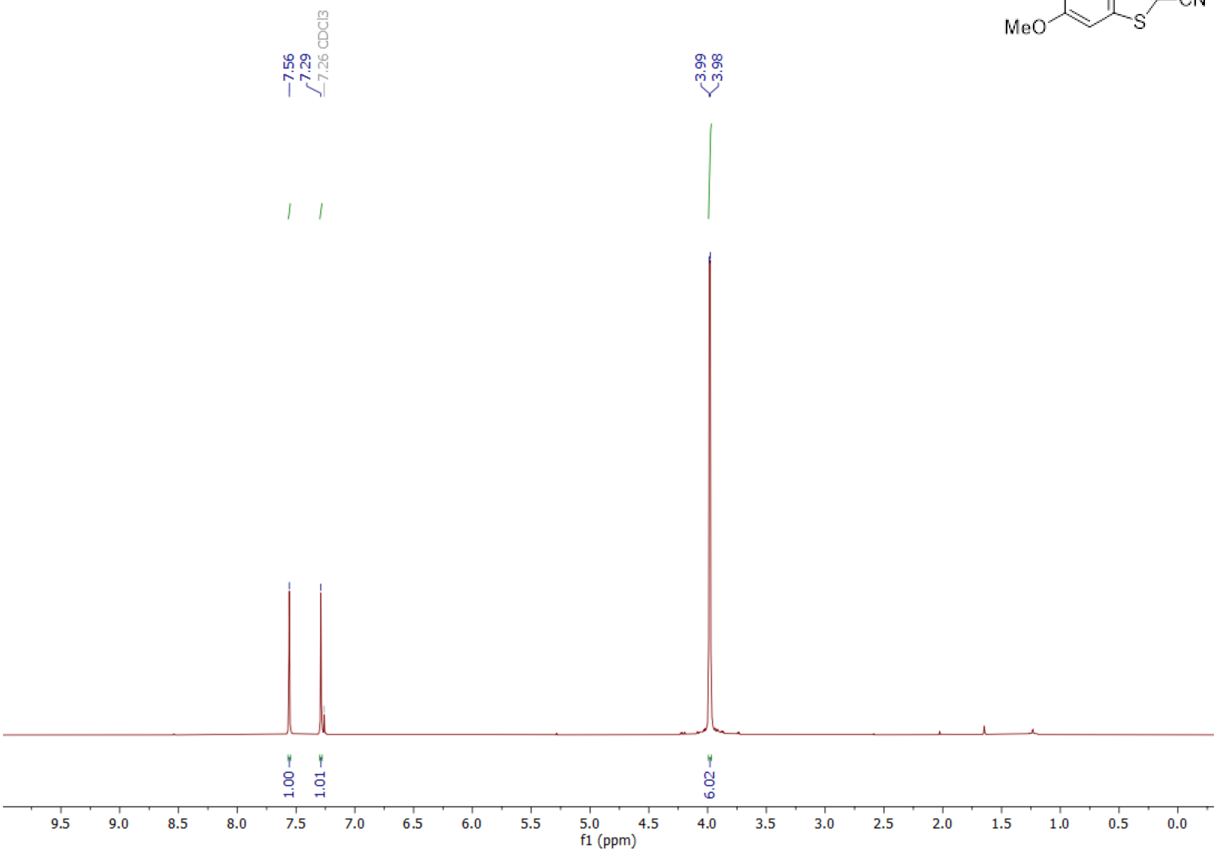
7.56
7.29
7.26 CDCl3
3.99
3.98
1.00
1.01
6.02
9.5 9.0 8.5 8.0 7.5 7.0 6.5 6.0 5.5 5.0 4.5 4.0 3.5 3.0 2.5 2.0 1.5 1.0 0.5 0.0
f1 (ppm)

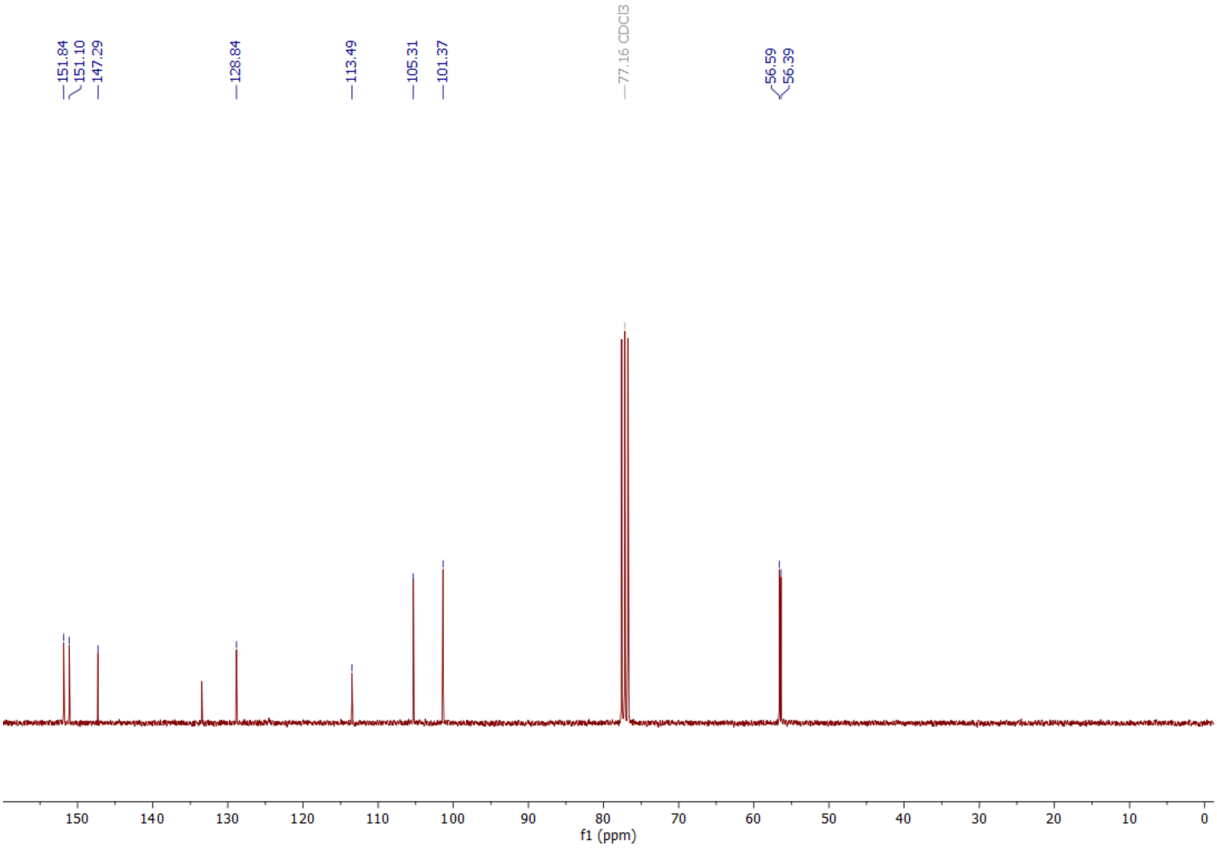
151.84
151.10
147.29
128.84
113.49
105.31
101.37
77.16 CDCl3
56.59
56.39
150 140 130 120 110 100 90 80 70 60 50 40 30 20 10 0
f1 (ppm)

4s

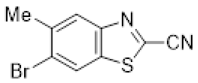

Me
N
CN
Br
S

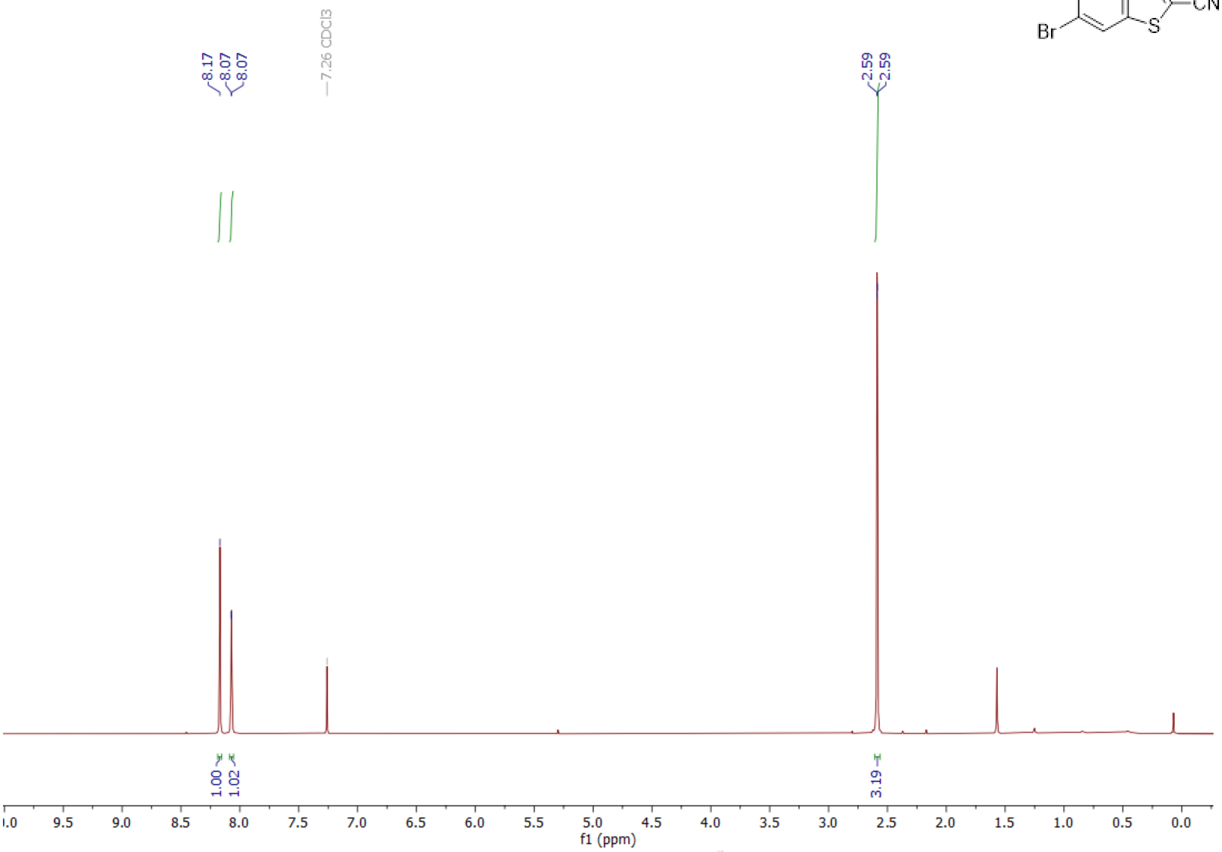

8.17
8.07
8.07
7.26 CDCl3
2.59
2.59
1.00
1.02
3.19
f1 (ppm)

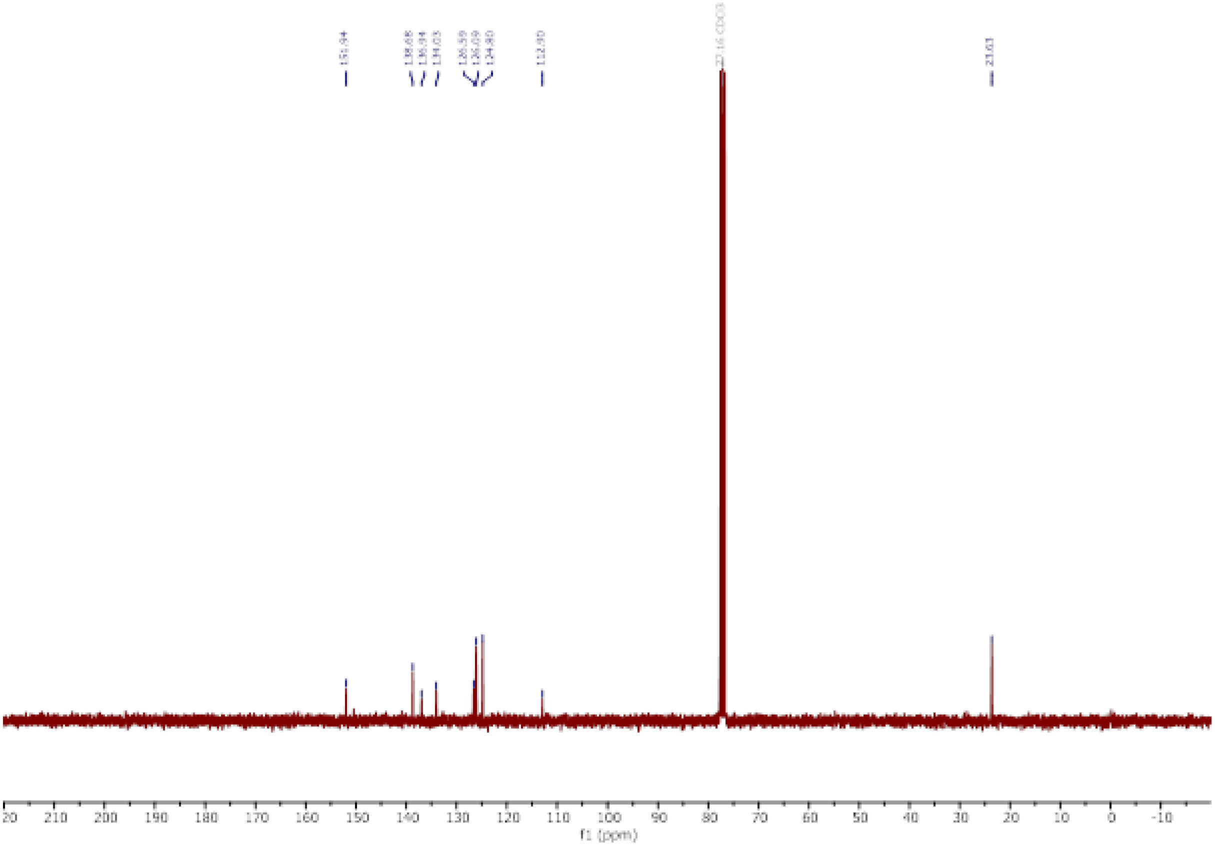

151.94
138.68
136.94
134.03
126.59
126.09
124.80
112.90
77.16 CDCl3
23.63
f1 (ppm)

4t

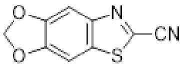
O
O
N
S
CN

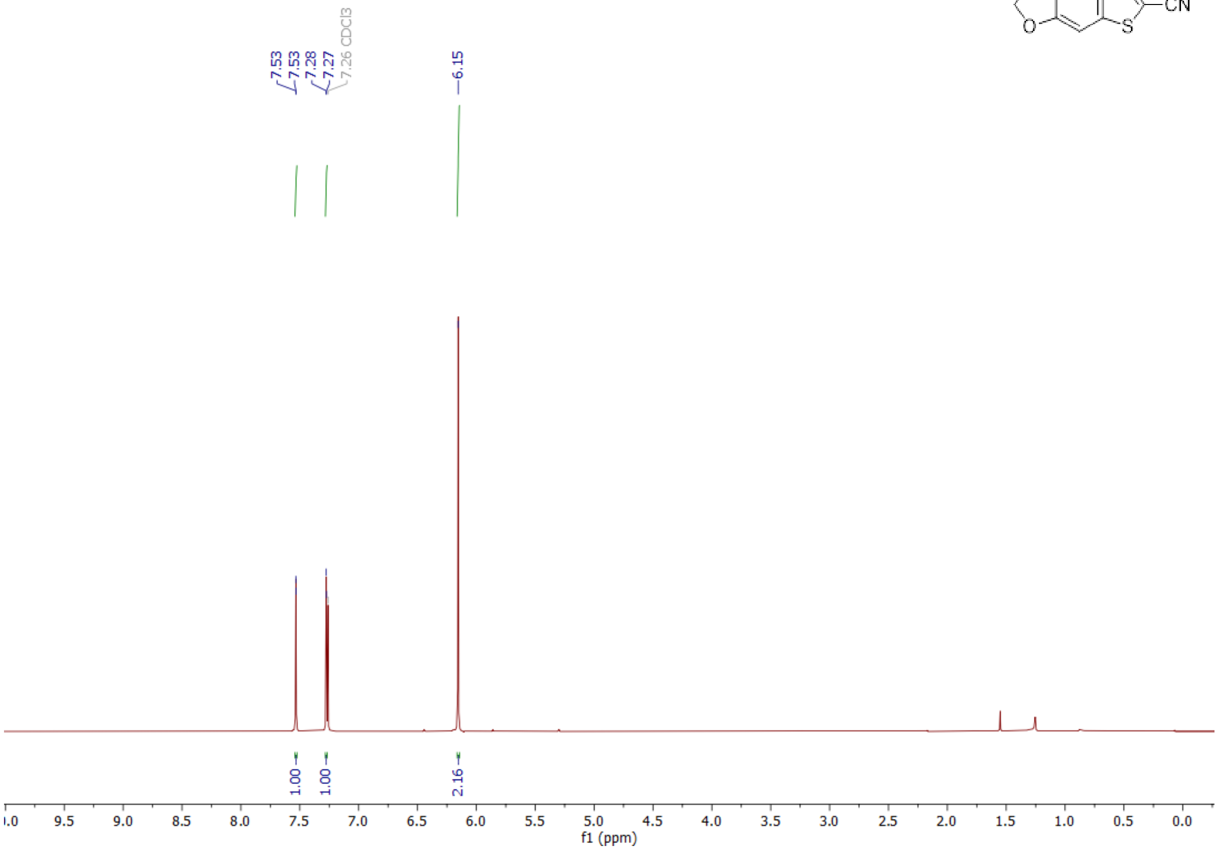
7.53
7.53
7.28
7.27
7.26 CDCl3
6.15
1.00
1.00
2.16
f1 (ppm)

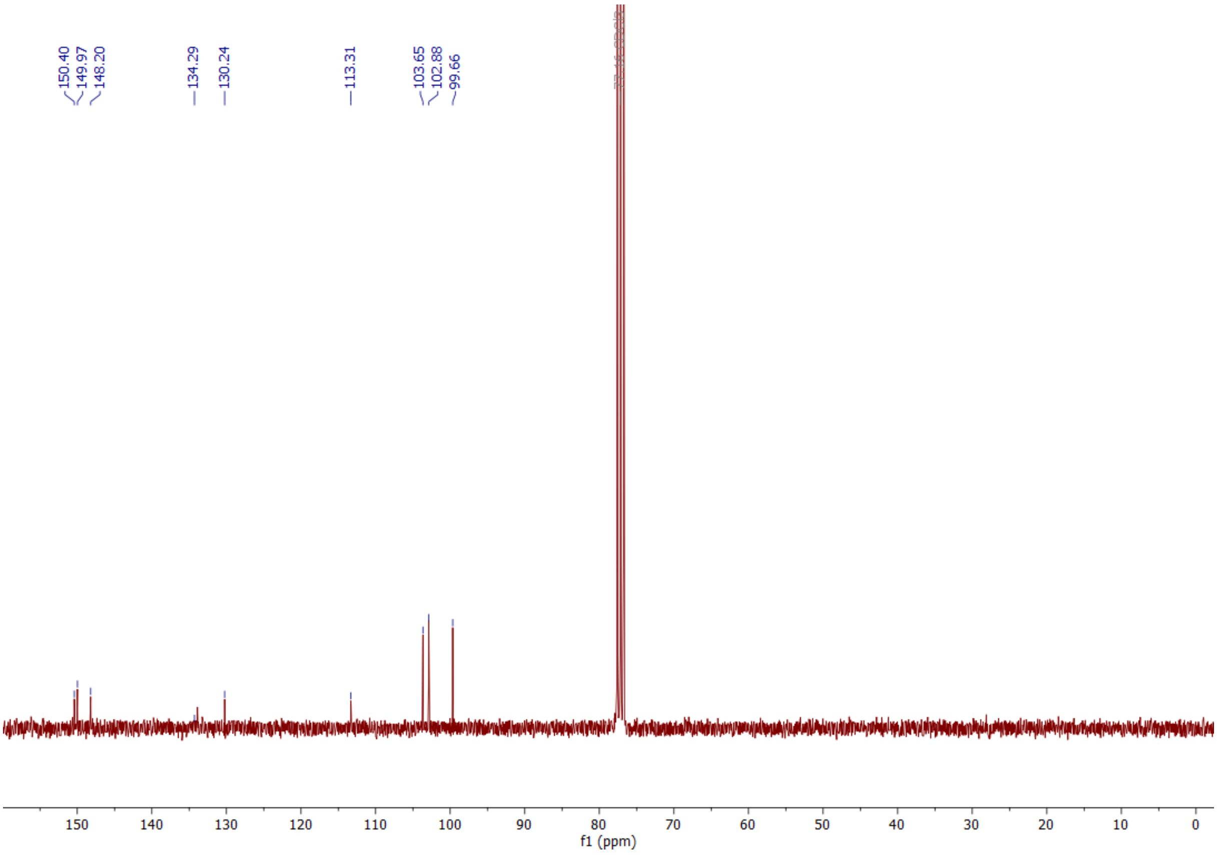
150.40
149.97
148.20
134.29
130.24
113.31
103.65
102.88
99.66
f1 (ppm)

4u

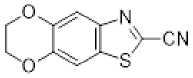

O
O
N
S
CN

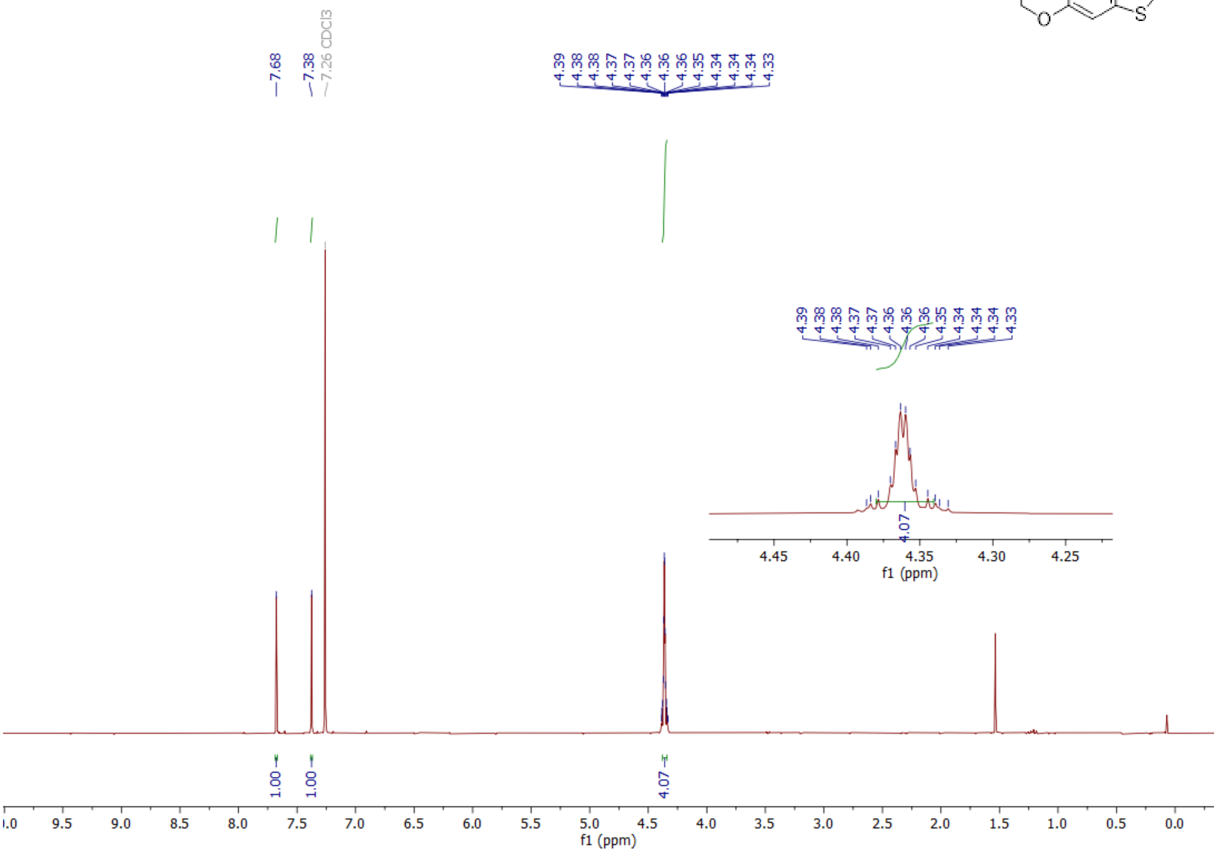

7.68
7.38
7.26 CDCl3
4.39
4.38
4.38
4.37
4.37
4.36
4.36
4.36
4.35
4.34
4.34
4.34
4.33
4.07
1.00
1.00
4.07
f1 (ppm)

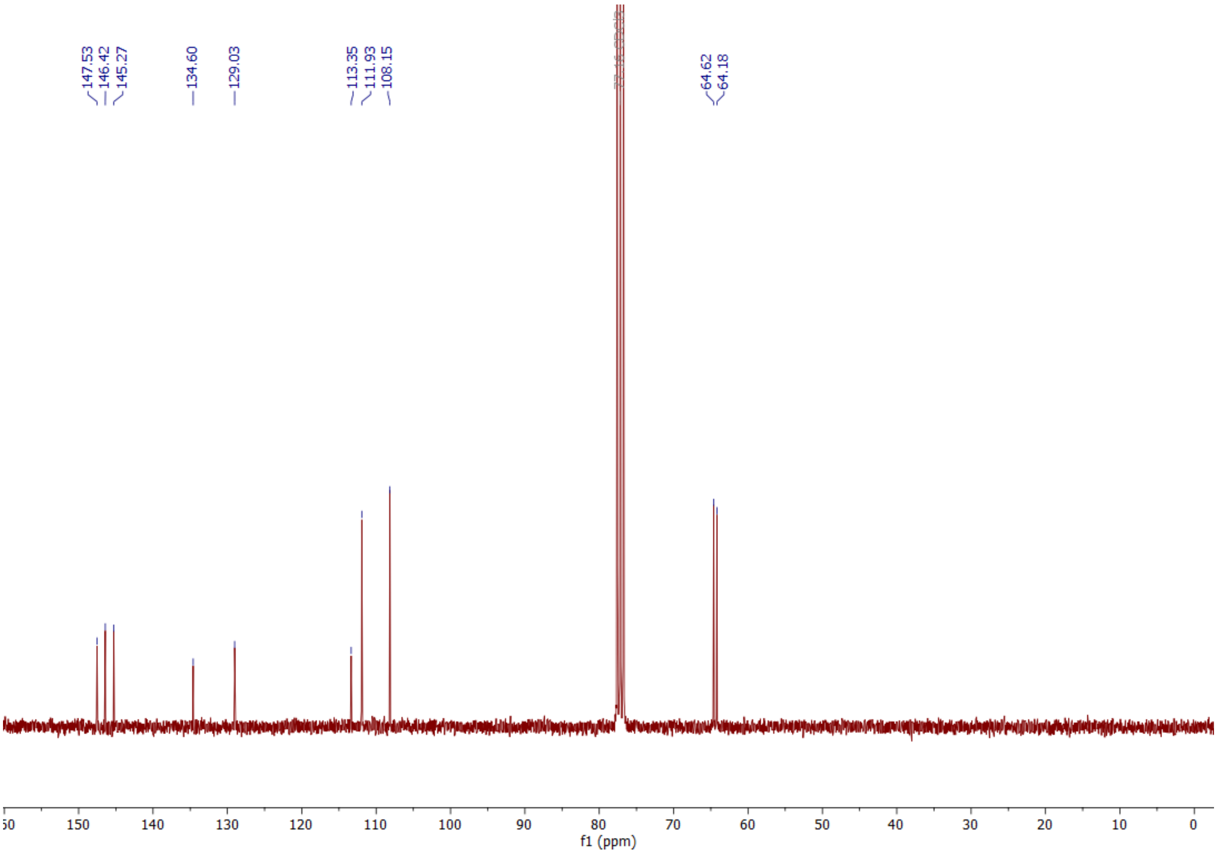

147.53
146.42
145.27
134.60
129.03
113.35
111.93
108.15
64.62
64.18
f1 (ppm)

4v

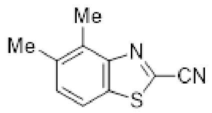
Me
Me
N
CN
S

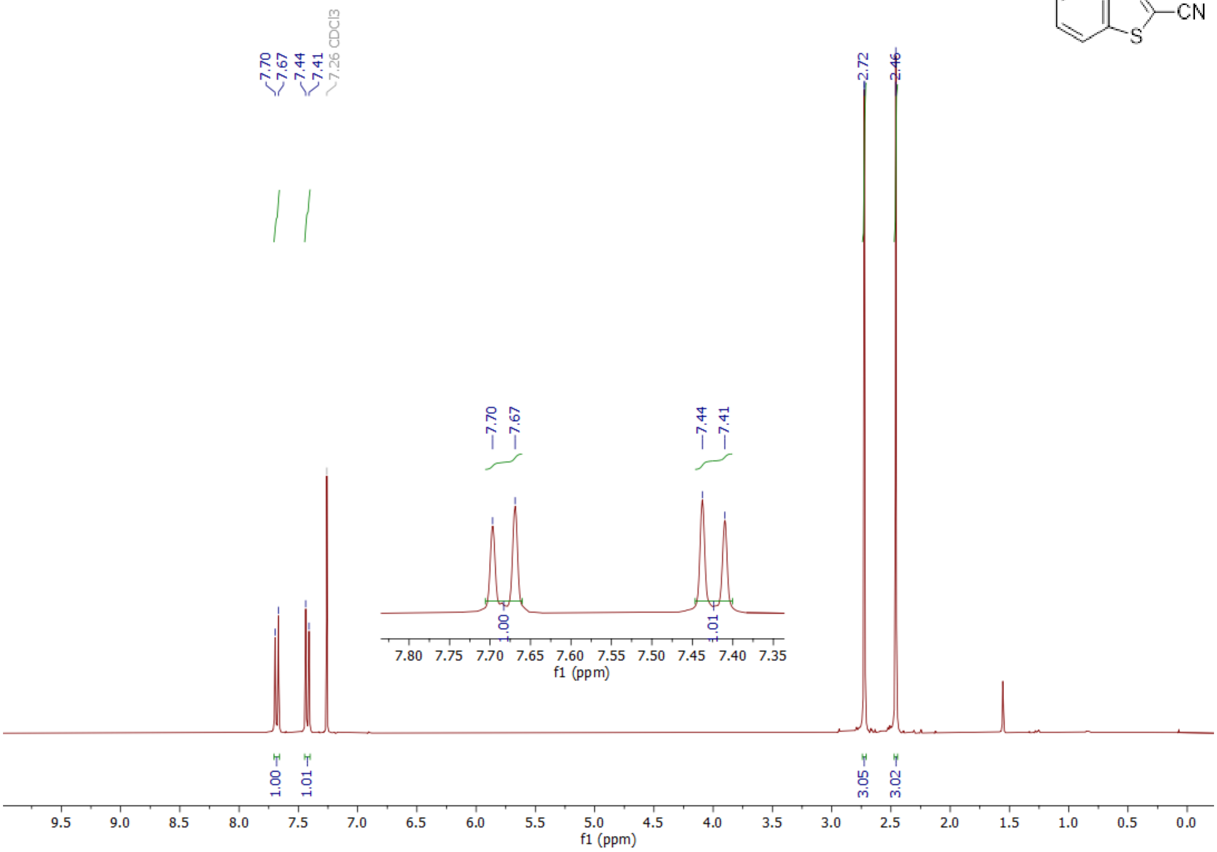
7.70
7.67
7.44
7.41
7.26 CDCl3
2.72
2.46
1.00
1.01
3.05
3.02
f1 (ppm)

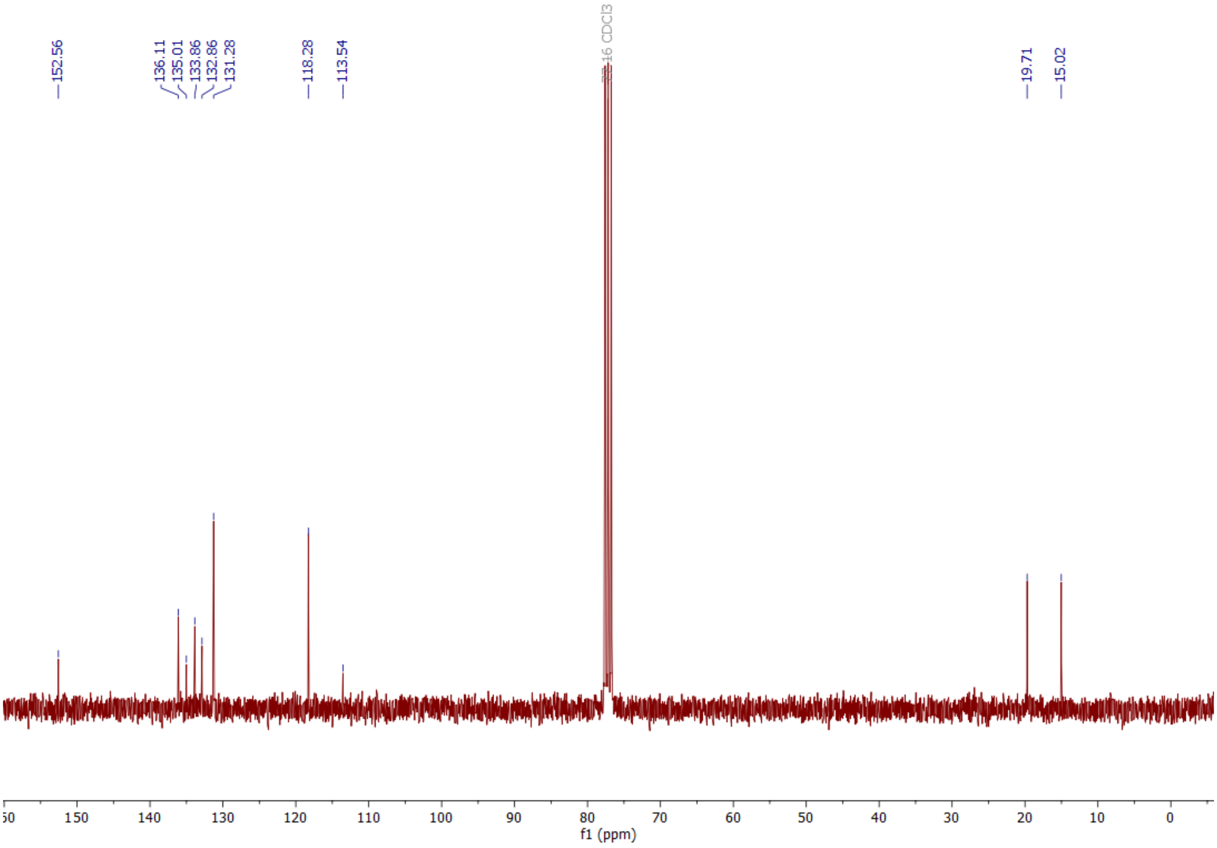
152.56
136.11
135.01
133.86
132.86
131.28
118.28
113.54
77.16 CDCl3
19.71
15.02
f1 (ppm)

4w

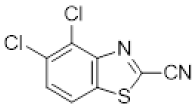
Cl
Cl
N
CN
S

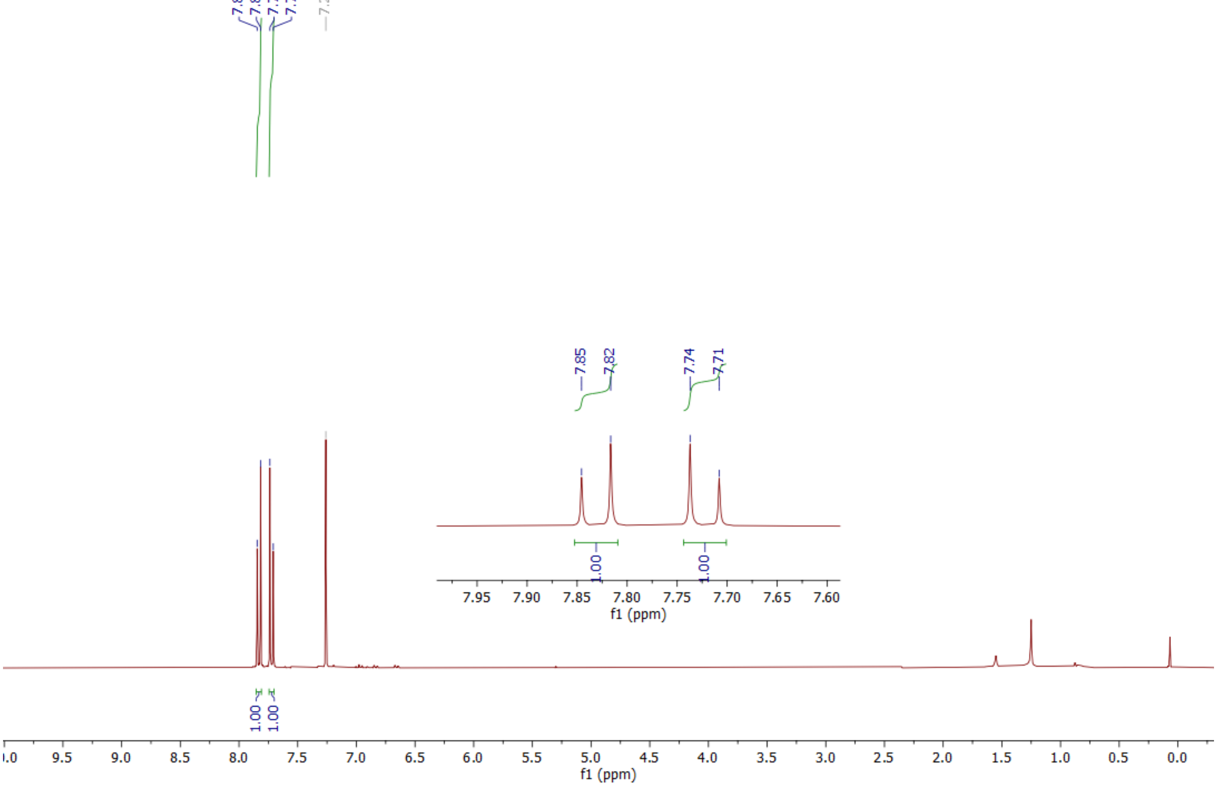
7.85
7.82
7.74
7.71
7.26 CDCl3
1.00
1.00
f1 (ppm)

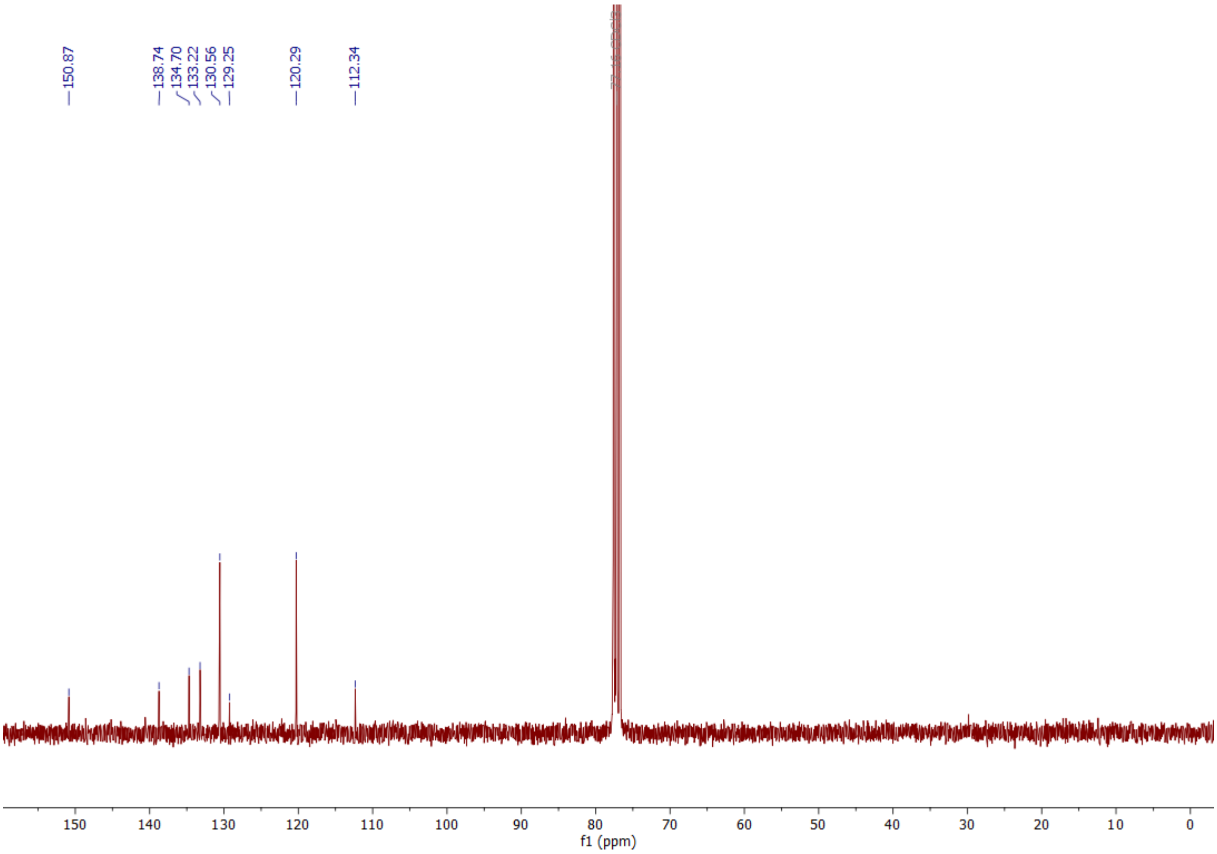
150.87
138.74
134.70
133.22
130.56
129.25
120.29
112.34
f1 (ppm)

4x

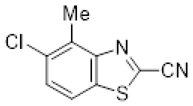

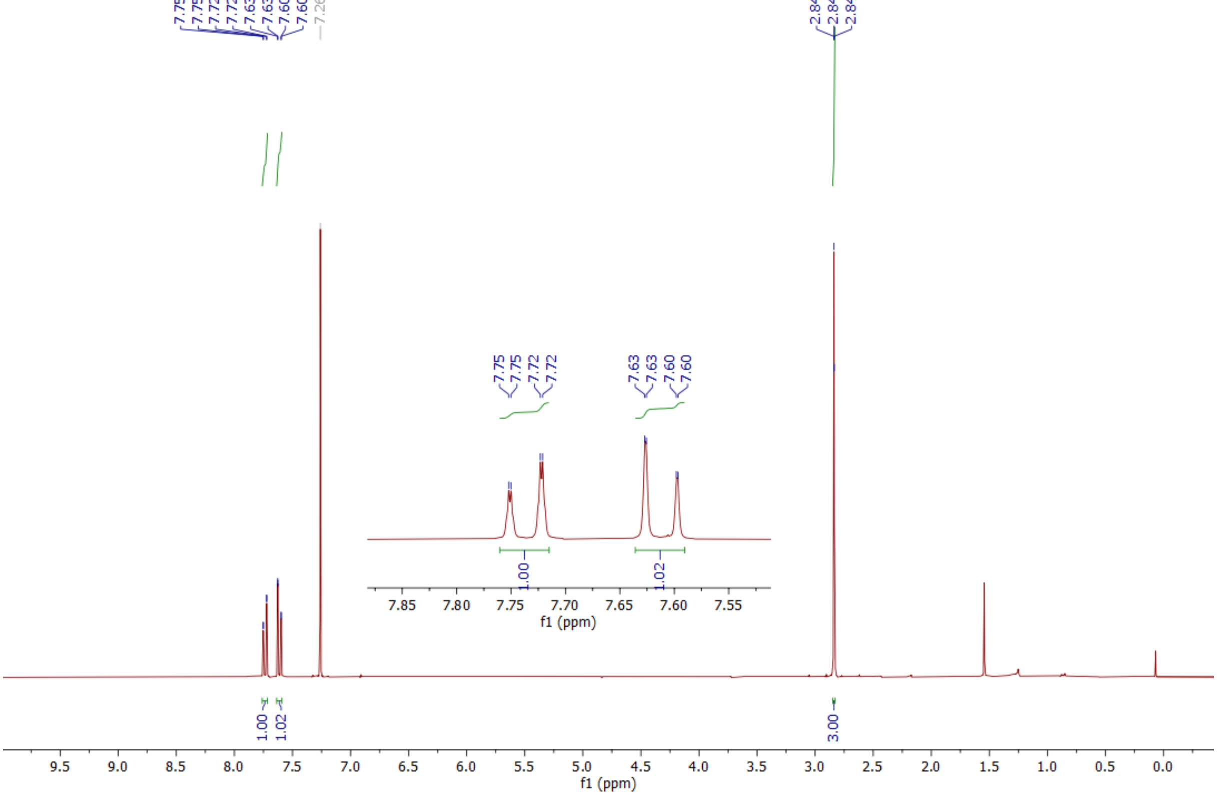

7.75
7.75
7.72
7.72
7.63
7.63
7.60
7.60
7.26 CDCl3
2.84
2.84
2.84
1.00
1.02
3.00
7.85
7.80
7.75
7.70
7.65
7.60
7.55
9.5
9.0
8.5
8.0
7.5
7.0
6.5
6.0
5.5
5.0
4.5
4.0
3.5
3.0
2.5
2.0
1.5
1.0
0.5
0.0
f1 (ppm)

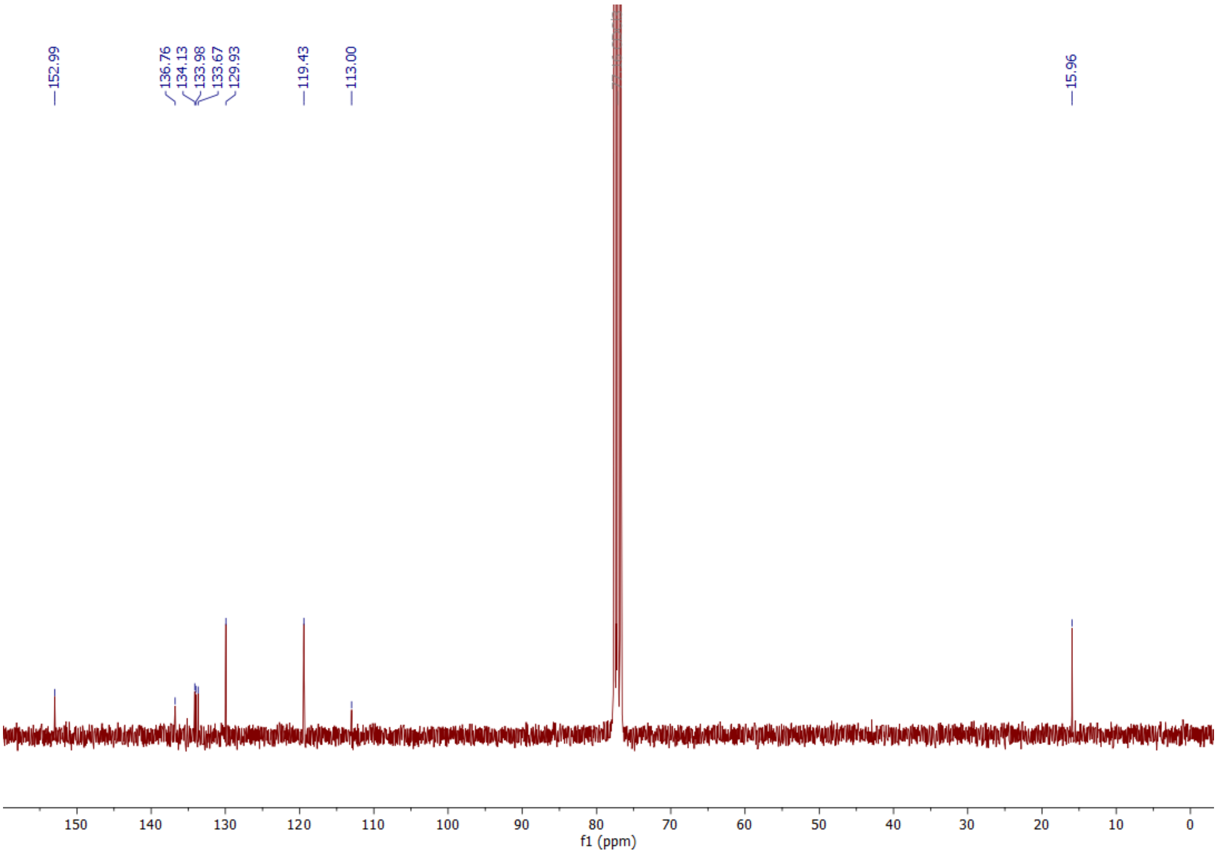

152.99
136.76
134.13
133.98
133.67
129.93
119.43
113.00
15.96
150
140
130
120
110
100
90
80
70
60
50
40
30
20
10
0
f1 (ppm)

4y

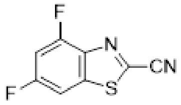
F
N
CN
F
S

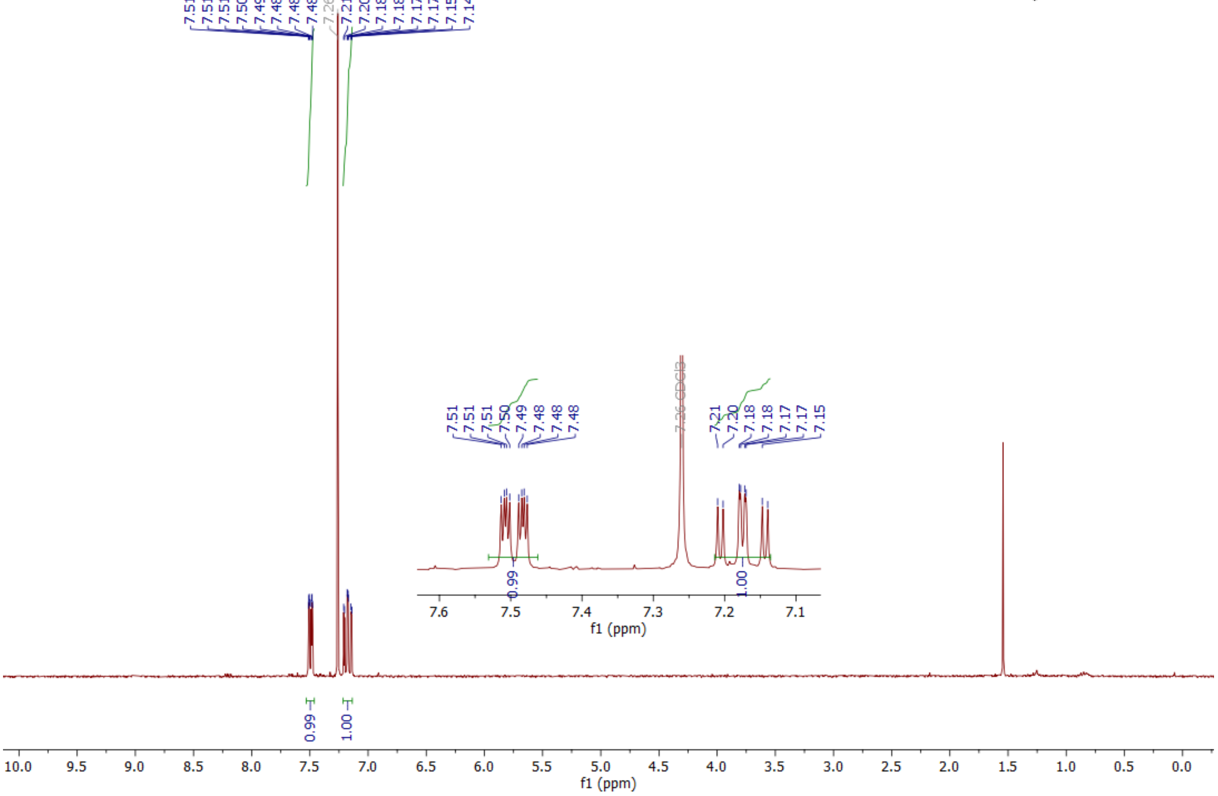
f1 (ppm)
0.99
1.00
7.26 CDCl3

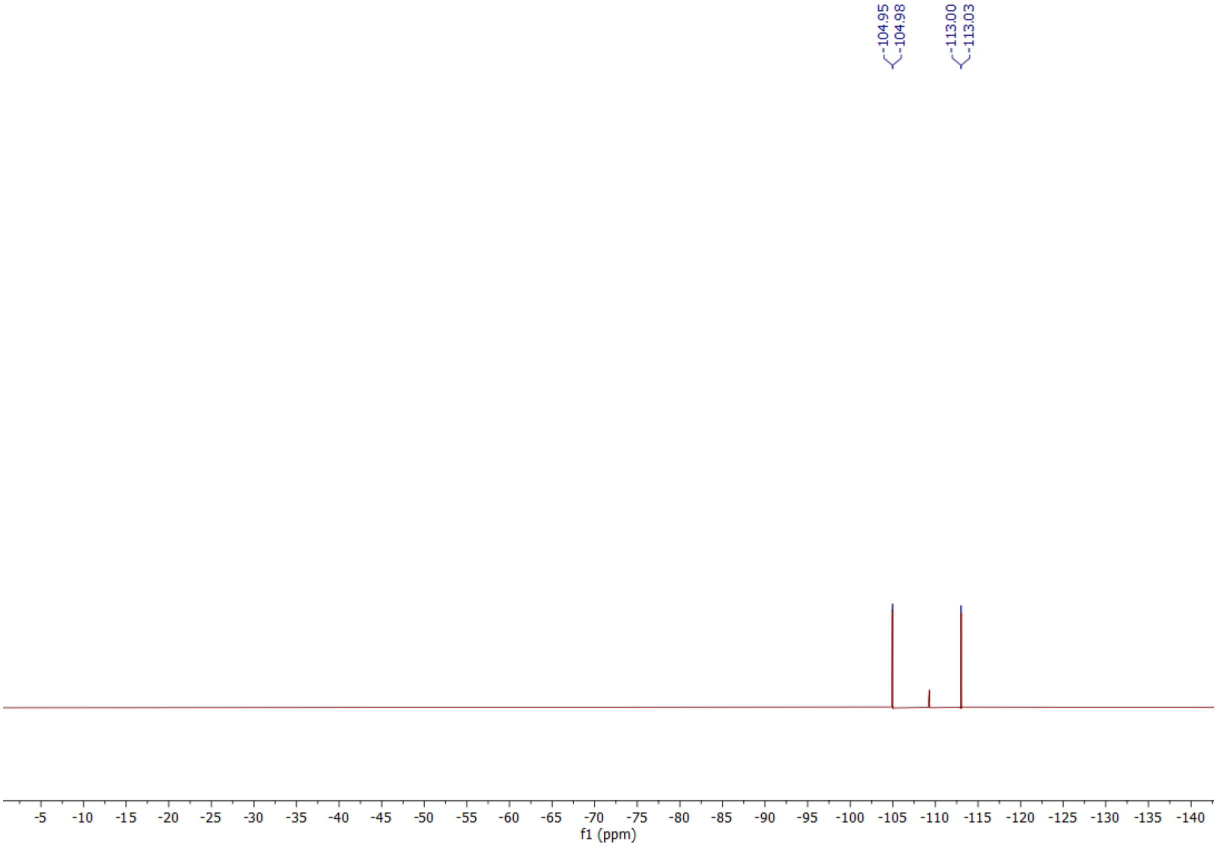
-104.95
-104.98
-113.00
-113.03
f1 (ppm)

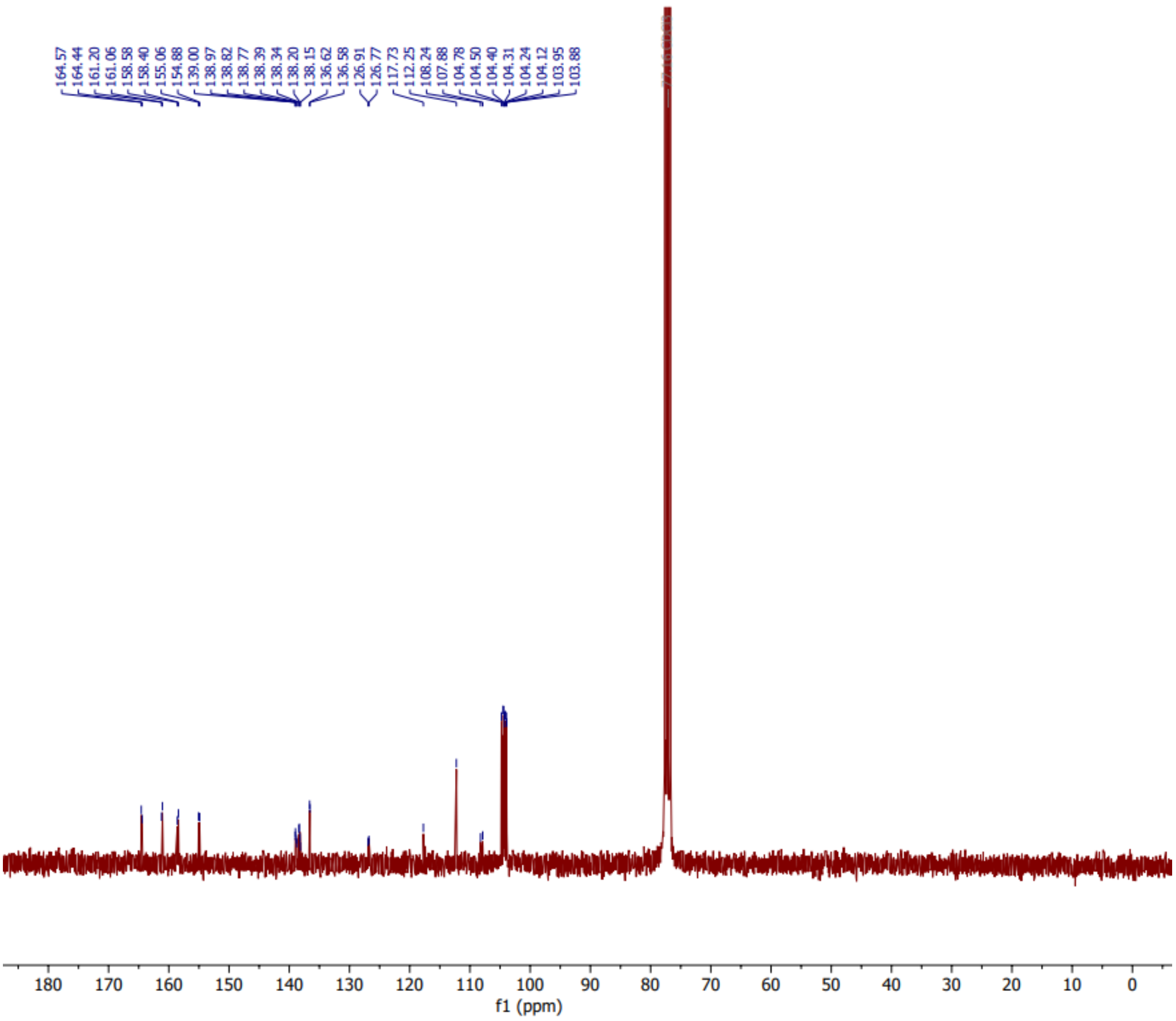
164.57
164.44
161.20
161.06
158.58
158.40
155.06
154.88
139.00
138.97
138.82
138.77
138.39
138.34
138.20
138.15
136.62
136.58
126.91
126.77
117.73
112.25
108.24
107.88
104.78
104.50
104.40
104.31
104.24
104.12
103.95
103.88
180 170 160 150 140 130 120 110 100 90 80 70 60 50 40 30 20 10 0
f1 (ppm)

4z

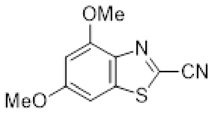

OMe
N
CN
MeO
S

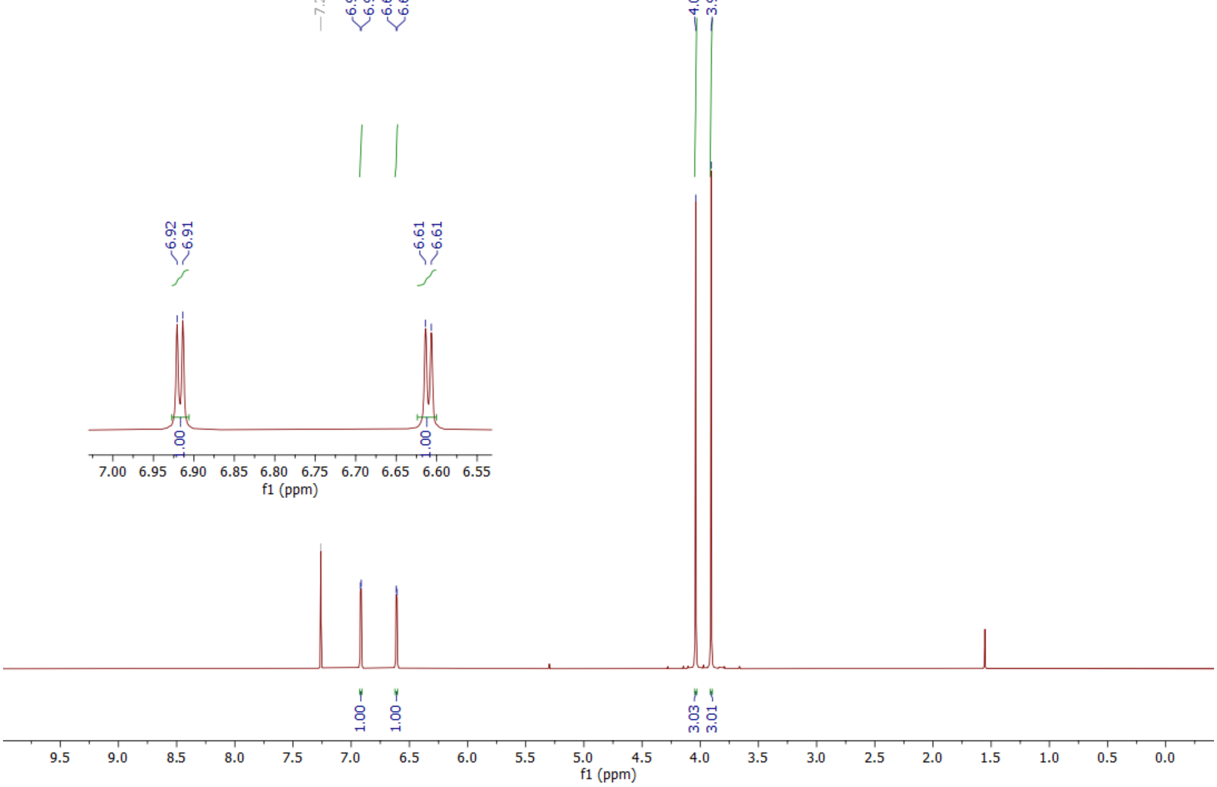

7.26 CDCl3
6.92
6.91
6.61
6.61
4.04
3.91
1.00
1.00
3.03
3.01
f1 (ppm)

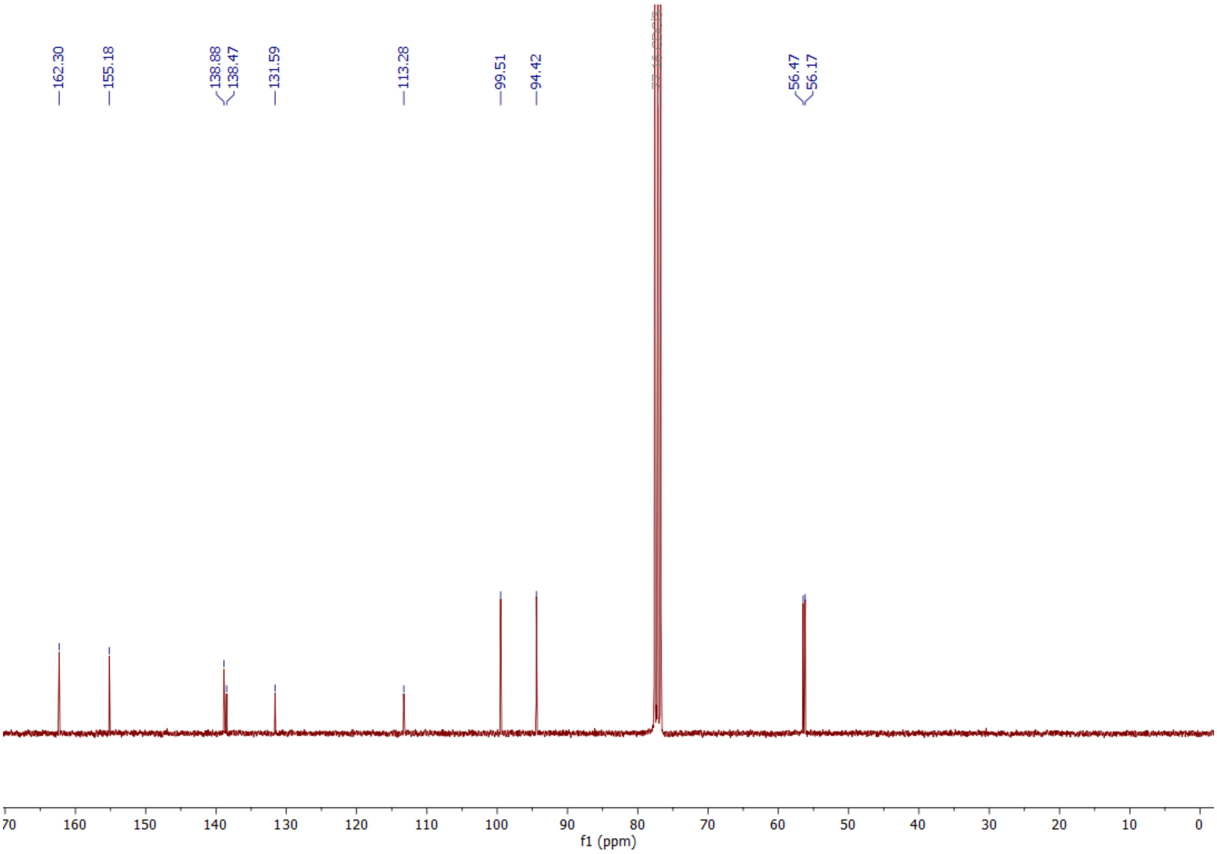

162.30
155.18
138.88
138.47
131.59
113.28
99.51
94.42
56.47
56.17
f1 (ppm)

4aa

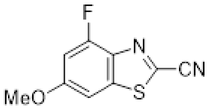
F
N
CN
MeO
S

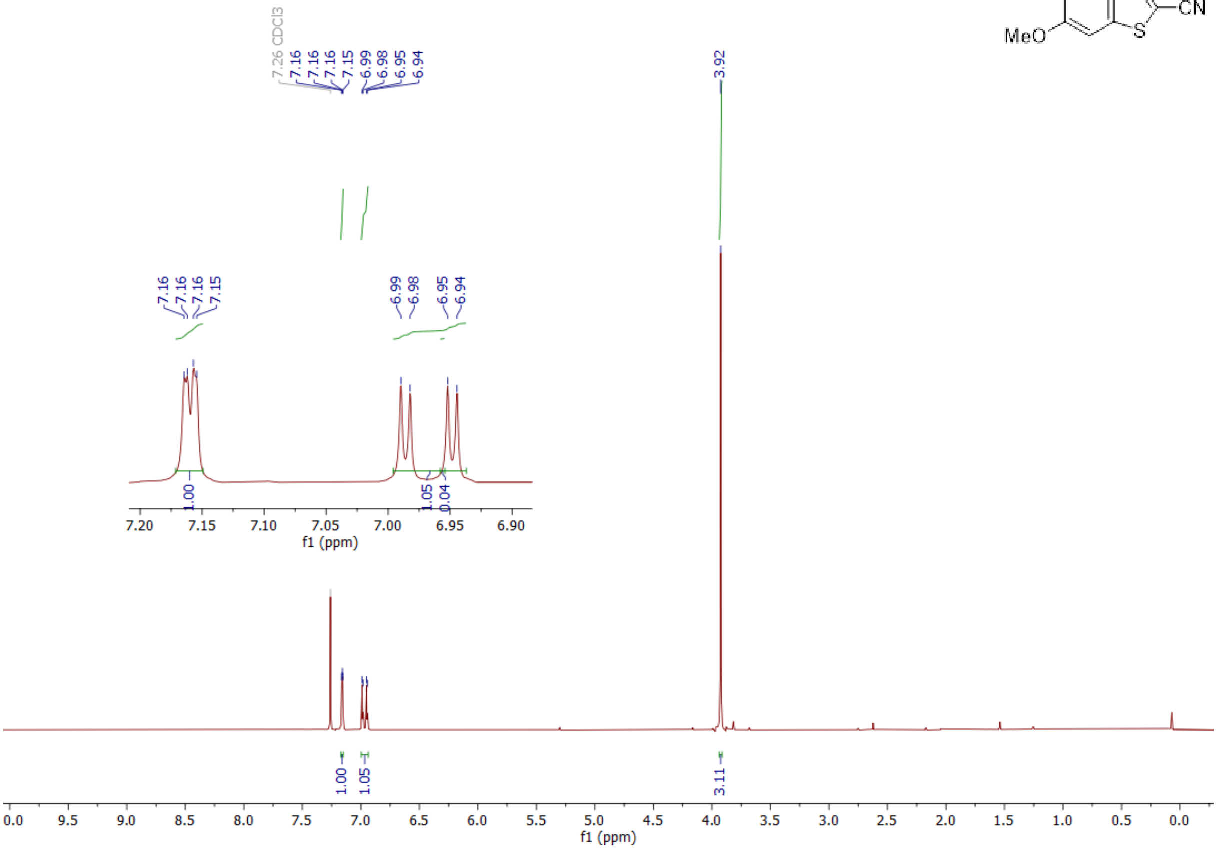
7.26 CDCl3
7.16
7.16
7.16
7.15
6.99
6.98
6.95
6.94
3.92
1.00
1.05
0.04
3.11
f1 (ppm)

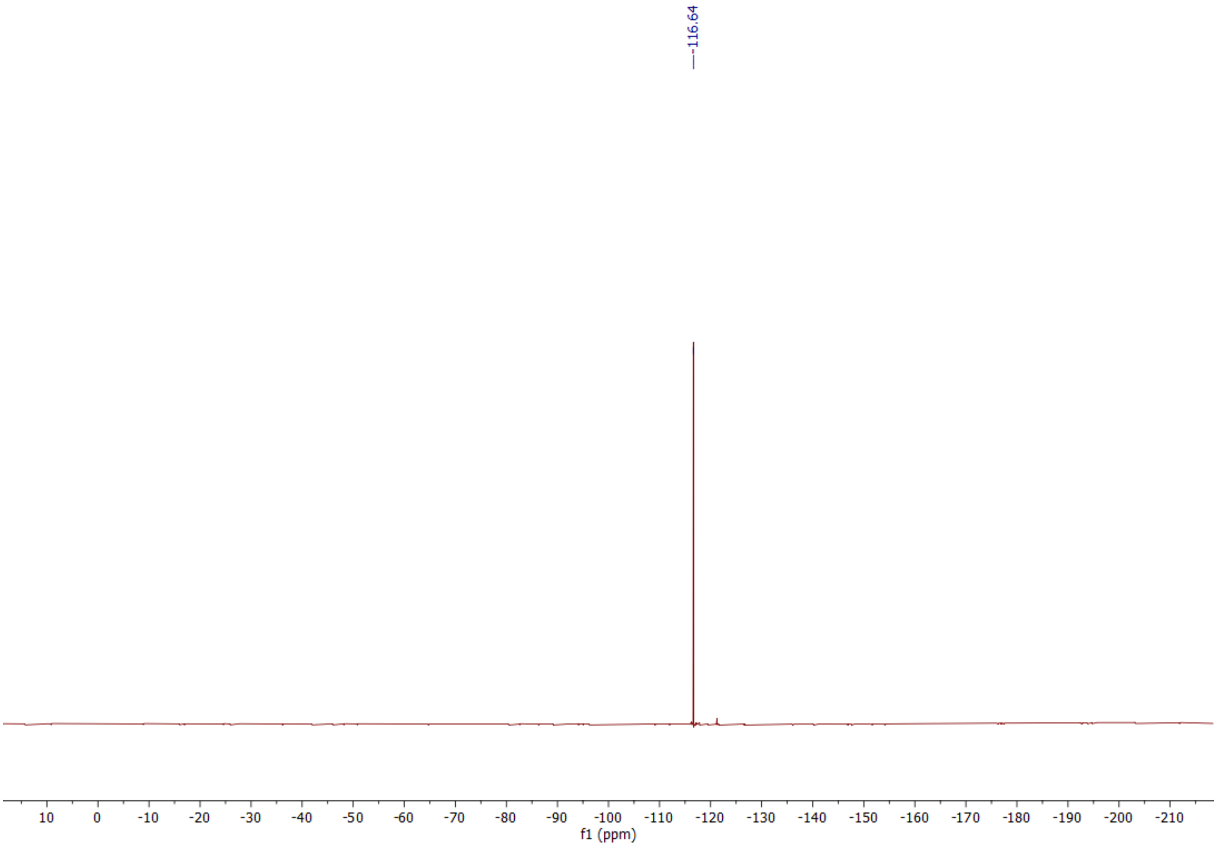
-116.64
f1 (ppm)

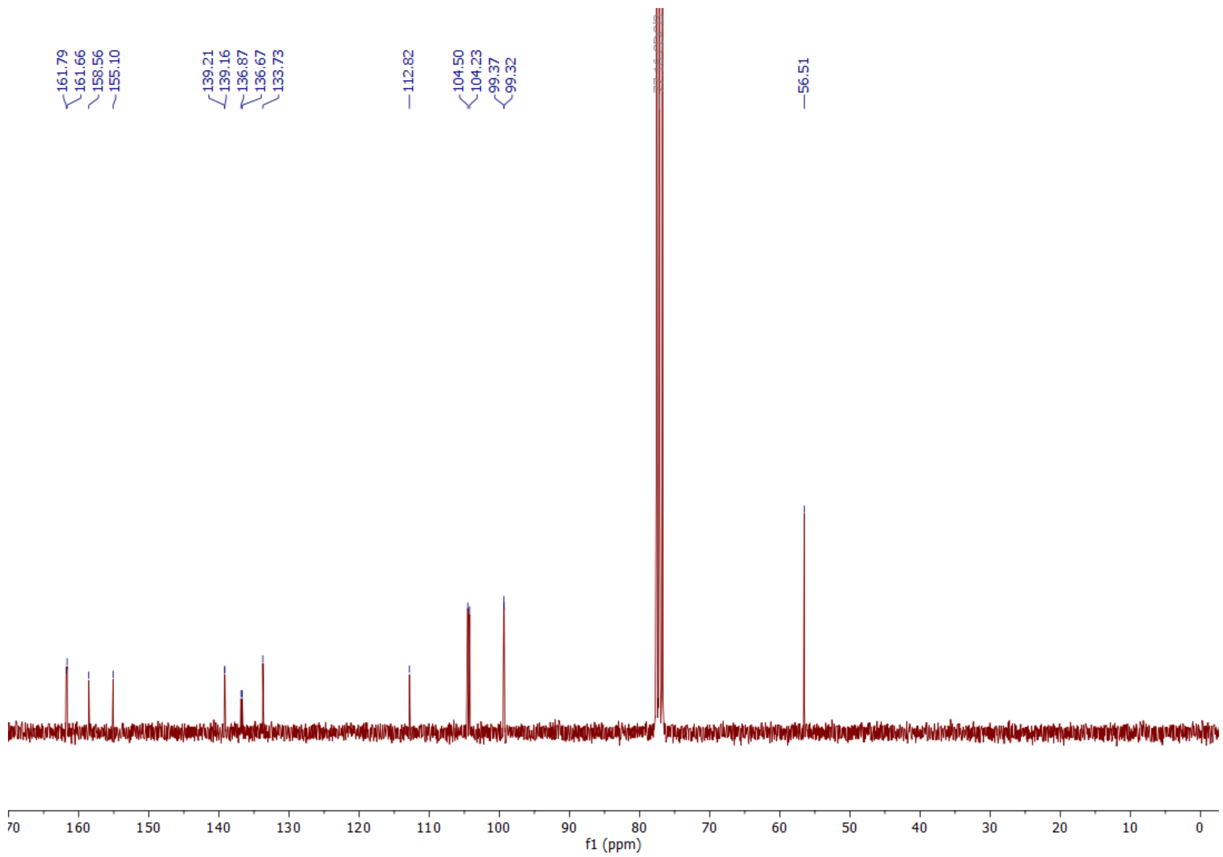
161.79
161.66
158.56
155.10
139.21
139.16
136.87
136.67
133.73
112.82
104.50
104.23
99.37
99.32
56.51
f1 (ppm)

4ab

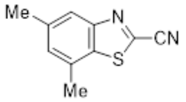

Me
N
CN
S
Me

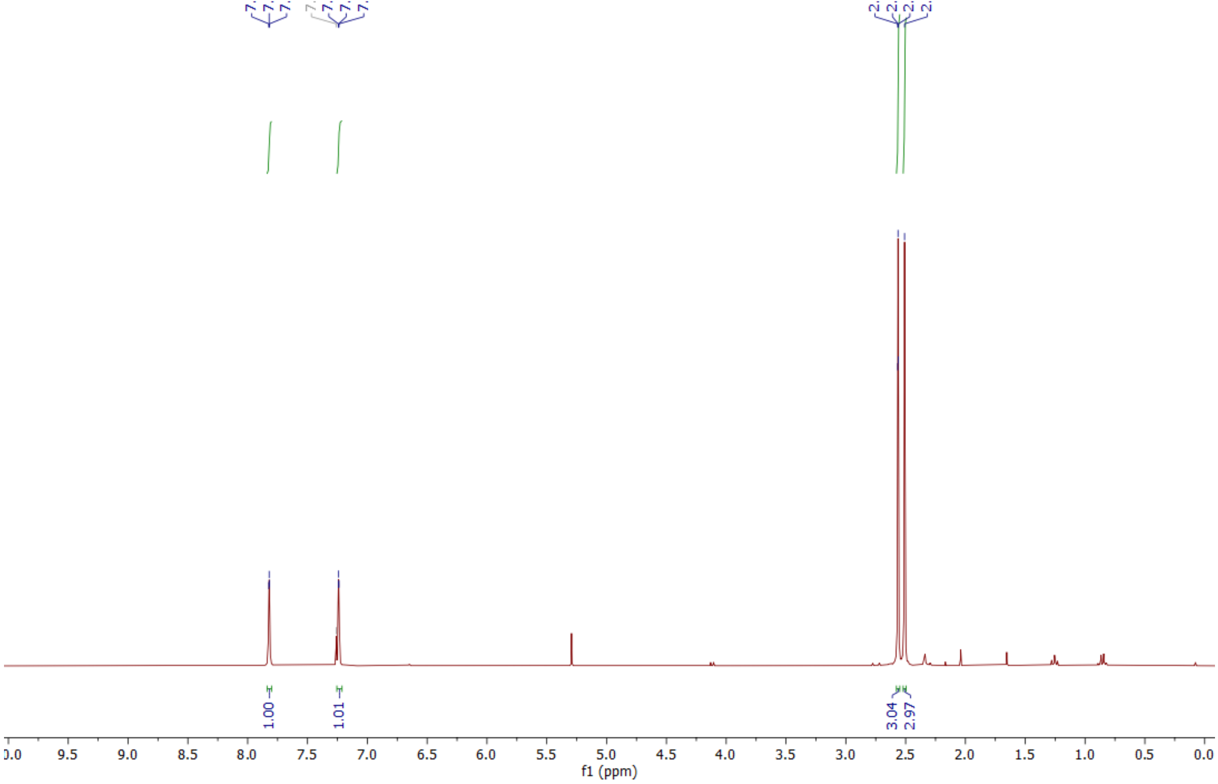

7.82
7.82
7.82
7.26 CDCl3
7.24
7.24
7.24
2.57
2.56
2.56
2.51
1.00
1.01
3.04
2.97
f1 (ppm)

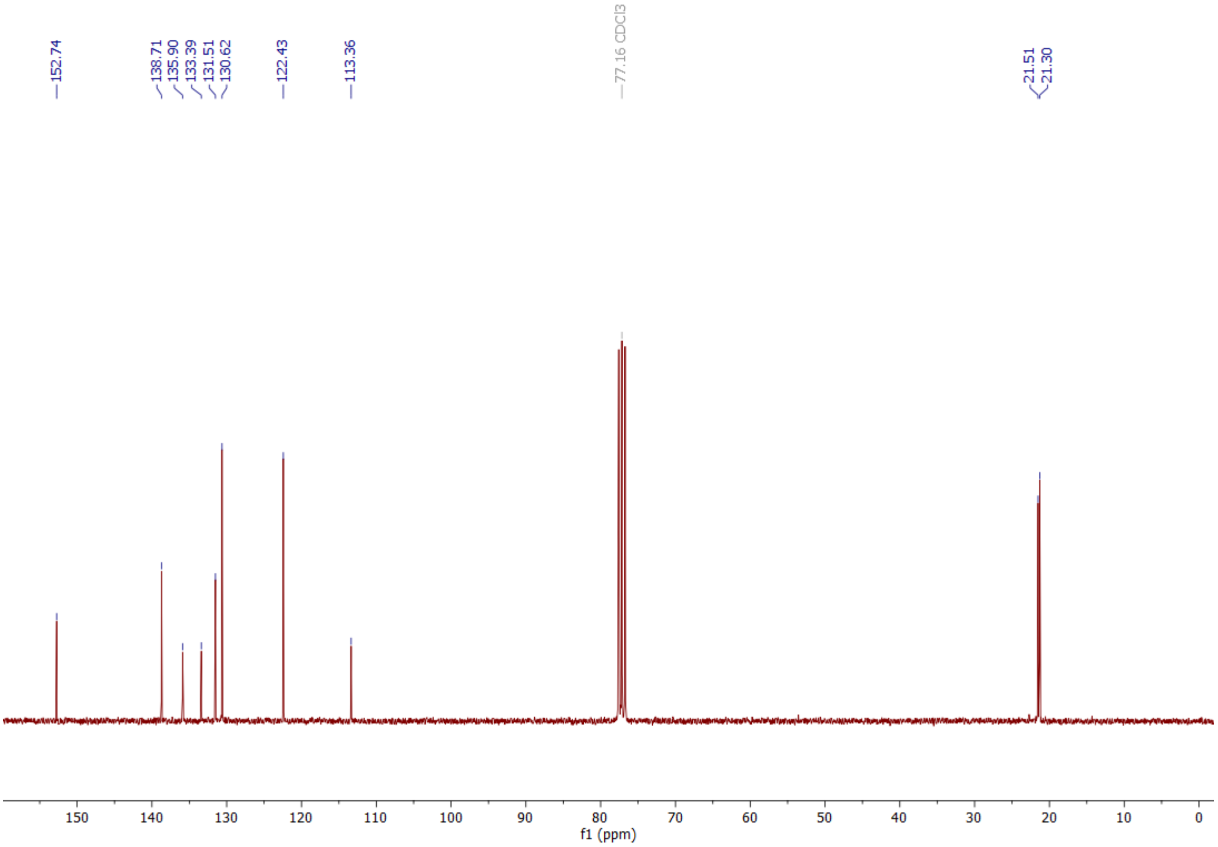

152.74
138.71
135.90
133.39
131.51
130.62
122.43
113.36
77.16 CDCl3
21.51
21.30
f1 (ppm)

4ac

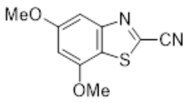

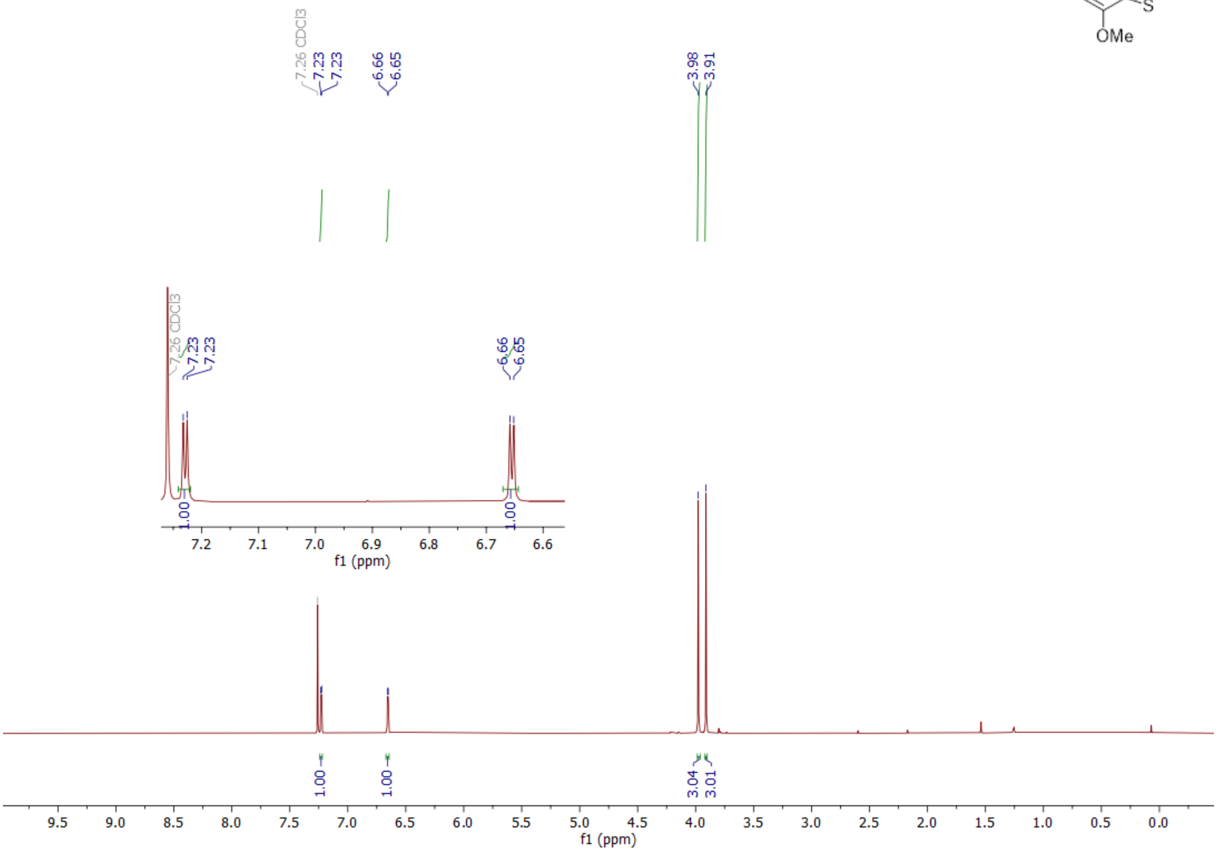

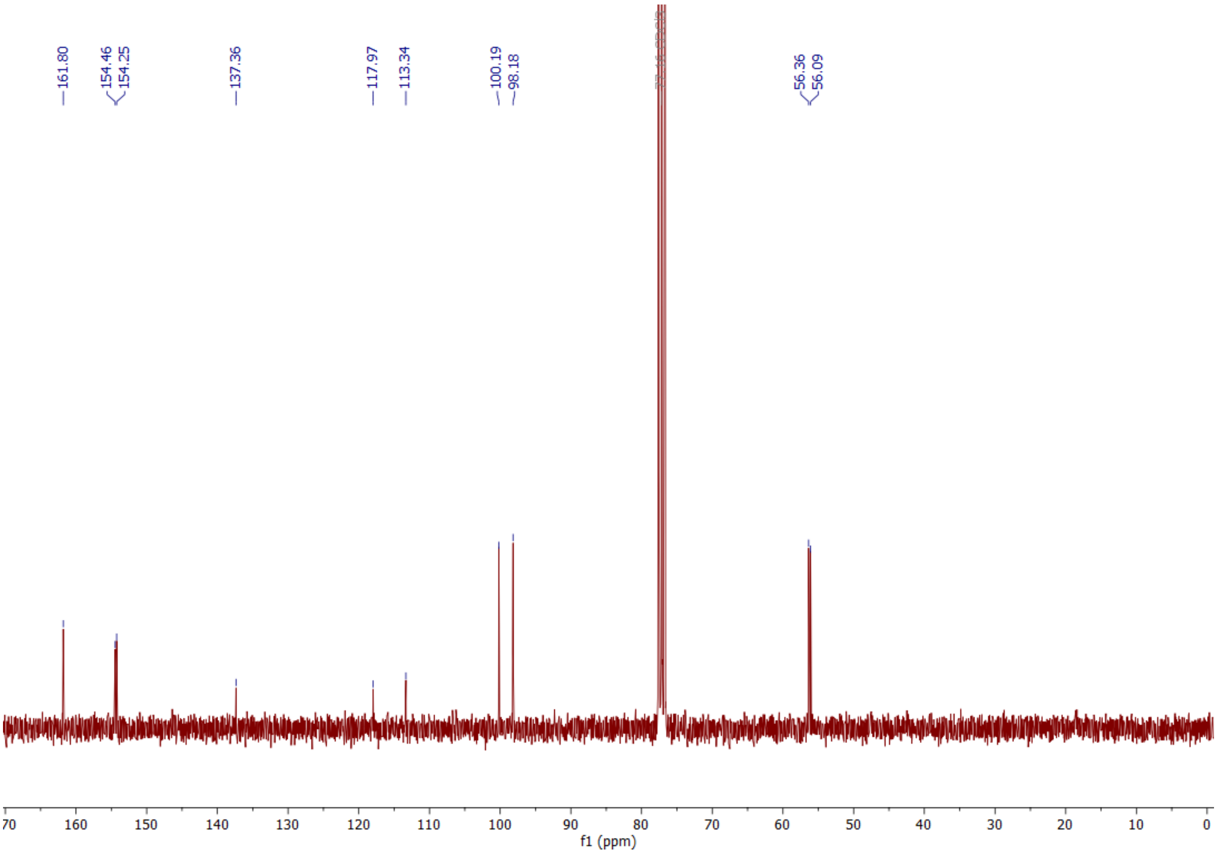

4ad′ & 4ad″

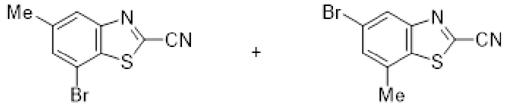

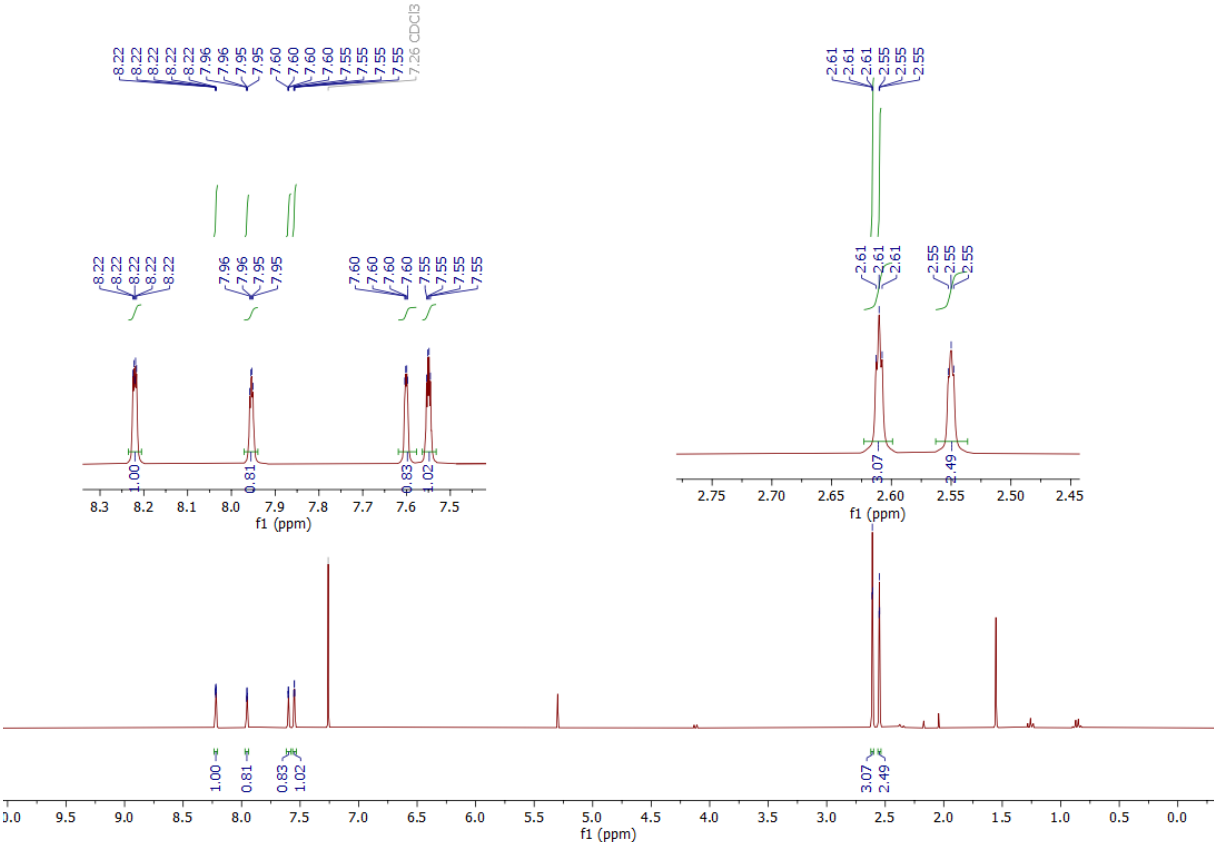

4ae'

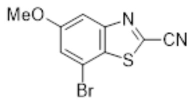

MeO
N
CN
S
Br

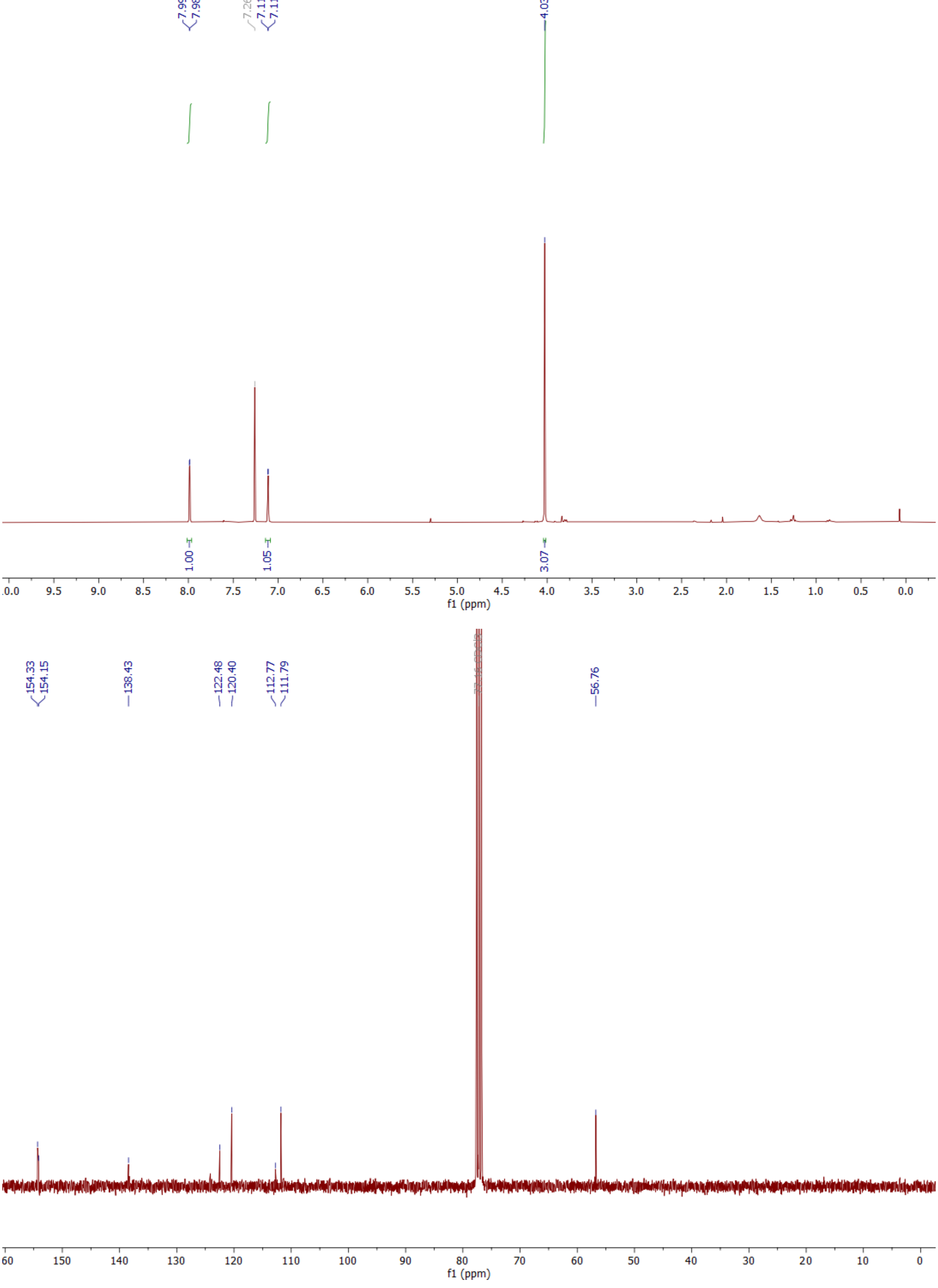

7.99
7.98
7.26 CDCl3
7.11
7.11
4.03
1.00
1.05
3.07
f1 (ppm)
154.33
154.15
138.43
122.48
120.40
112.77
111.79
56.76
f1 (ppm)

4ae''

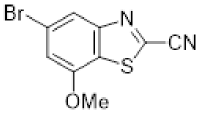
Br
N
CN
S
OMe

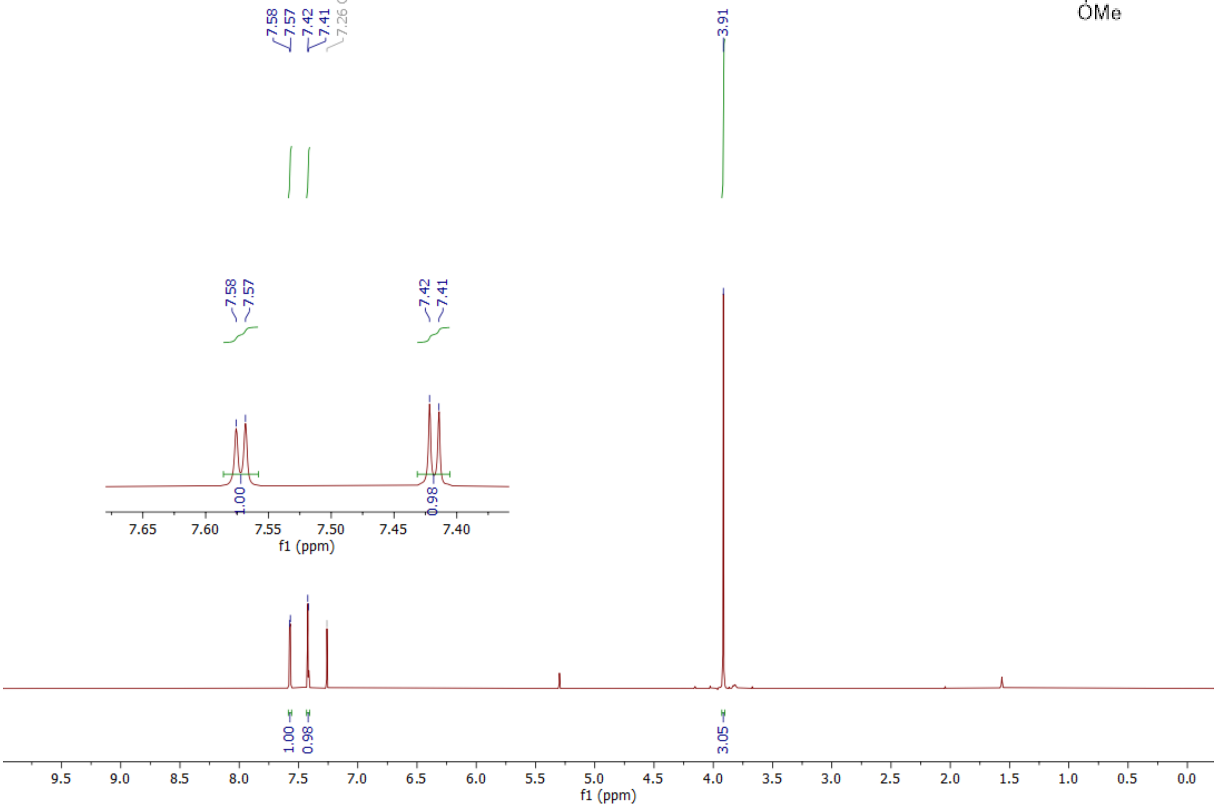
7.58
7.57
7.42
7.41
7.26 CDCl3
3.91
1.00
0.98
3.05
f1 (ppm)

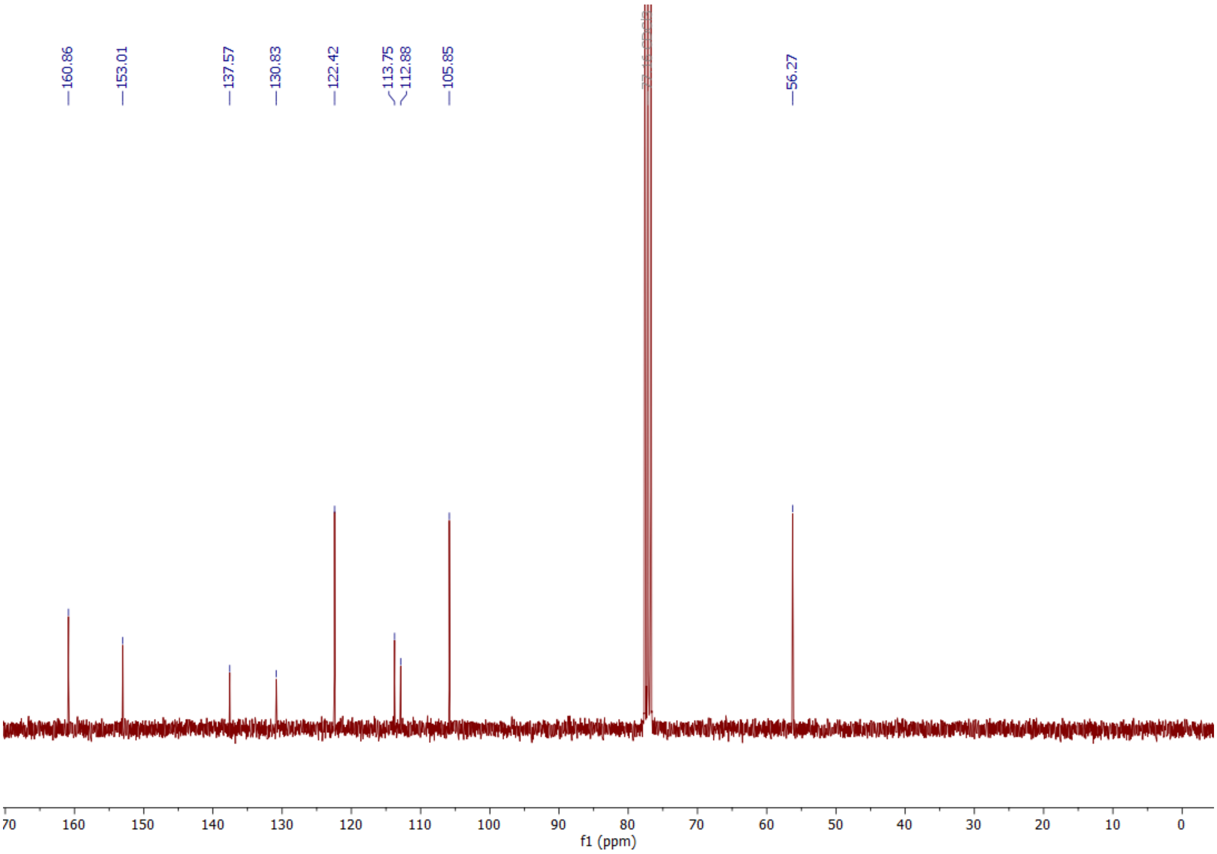
160.86
153.01
137.57
130.83
122.42
113.75
112.88
105.85
56.27
f1 (ppm)
